# Associations Between Biomarkers of Alzheimer's Disease and Sleep in Cognitively Unimpaired Older Adults

**DOI:** 10.1002/acn3.70562

**Published:** 2026-10-21

**Authors:** Cornelia Sauter, Hans Dorn, Julian Hellmann‐Regen, Ana Bueno‐Lopez, Verena van Ginneken, Heidi Danker‐Hopfe

**Affiliations:** ^1^ Department of Psychiatry and Neurosciences, Competence Center of Sleep Medicine Charité ‐ Universitätsmedizin Berlin, Corporate Member of the Freie Universität at Berlin and Humboldt Universität zu Berlin Berlin Germany; ^2^ Department of Psychiatry and Neurosciences, Clinical Neurobiology Lab Charité ‐ Universitätsmedizin Berlin, Corporate Member of the Freie Universität at Berlin and Humboldt Universität zu Berlin Berlin Germany; ^3^ Sant Pau Memory Unit Institut de Recerca Sant Pau, Hospital de la Santa Creu i Sant Pau Barcelona Spain

**Keywords:** EEG spectral analysis, sleep quality, sleep spindles

## Abstract

**Objective:**

Sleep disturbances have been implicated as a risk factor for developing Alzheimer's disease (AD), but evidence linking plasma AD biomarkers to sleep in cognitively unimpaired individuals remains limited. The present study examined associations between evening, morning, and overnight changes in plasma AD biomarkers and measures of sleep macro‐ and microstructure and subjective sleep quality in older adults.

**Methods:**

Forty cognitively unimpaired adults aged 55 to 75 years (20 females) without sleep or mental disorders underwent overnight polysomnography. Plasma AD biomarkers p‐tau181, p‐tau217, t‐tau, Aβ42, Aβ40, and Aβ42/40 were assessed in the evening and in the morning and related to sleep macro‐ and microstructure (sleep spindles, EEG spectral power), and subjective sleep measures by using correlational analyzes.

**Results:**

Higher evening t‐tau and p‐tau181 were associated with reduced N3 sleep, whereas higher morning t‐tau and greater overnight increases in t‐tau were related to longer nocturnal wakefulness. Amyloid‐β measures showed the most consistent associations with sleep microstructure. Higher Aβ42 and Aβ40 concentrations were associated with shorter duration and lower density of sleep spindles, particularly at frontal sites. Higher evening and morning amyloid‐β concentrations were also associated with lower alpha, beta, and spindle‐frequency EEG power, mainly during non‐rapid eye movement sleep. Tau associations with EEG spectral power were less consistent.

**Interpretation:**

In cognitively unimpaired older adults, plasma AD biomarkers were associated with PSG sleep features, especially amyloid‐β markers with sleep microstructure. These exploratory, correlational findings suggest that sleep spindles and spectral power may reflect early AD‐related biological processes but require replication in larger cohorts.

## 1 Introduction

According to the World Health Organization (WHO, 2025) [1], approximately 57 million people worldwide were living with dementia in 2021, with nearly 10 million new cases diagnosed annually. Alzheimer disease (AD), the most common form, accounts for approximately 60%–70% of cases. Emerging evidence suggests a bidirectional relationship between disturbed sleep and AD, indicating that sleep‐promoting interventions that reduce the risk of sleep disturbances may play a role in AD prevention [2, 3, 4, 5].

While most of the studies relied on self‐reported or actigraphy data, Zhang et al. [6] analyzed polysomnographic findings from 28 studies. AD patients showed significantly reduced total sleep time (TST), sleep efficiency, slow wave sleep (SWS), and rapid eye movement (REM) sleep, alongside increased sleep latency, wake after sleep onset (WASO), REM latency, and awakenings compared with healthy controls. Reduced SWS and REM sleep correlated with greater cognitive impairment. The authors also reported alterations in sleep microstructure, including sleep spindles and spectral components of electroencephalography (EEG), suggesting their potential as biomarkers for AD diagnosis.

Beyond the high prevalence of sleep disturbances in AD, evidence suggests that impaired sleep is a risk factor for AD development. A meta‐analysis of 27 observational studies found that individuals with sleep problems had a higher risk of AD (relative risk, RR 1.55), cognitive impairment (RR 1.65), and preclinical AD (RR 3.78) [7]. Another systematic review of 35 longitudinal studies showed that insomnia, sleep‐disordered breathing, and other sleep disturbances were associated with an increased AD risk [8]. Insomnia has also been identified as a risk factor for AD in a systematic review of 18 longitudinal studies including 246,768 participants, which reported 25,847 dementia cases after a mean follow‐up period of 9.5 years [9].

Wang and Holtzman [10] proposed that sleep influences the accumulation of amyloid‐beta (Aβ) and tau proteins, key neuropathological hallmarks of AD. Their experimental studies showed that sleep disturbance and increased wakefulness raise Aβ production, reduce its clearance, and promote aggregation, while Aβ accumulation in turn disrupts sleep in both animals and humans. Additionally, acute sleep deprivation increases cerebrospinal fluid tau levels in humans, and chronic sleep deprivation accelerates tau spread in neural networks, correlating with decreased non‐rapid eye movement (NREM) slow wave activity (SWA). Moreover, a study that specifically disrupted SWA showed that this disruption was associated with increased Aβ levels, suggesting a link between sleep disturbance and specific changes in neuronal activity [11]. Despite strong evidence for this bidirectional relationship, data in humans remain limited [3].

A recent systematic review and meta‐analysis of 30 human studies examined associations between sleep quality or sleep duration and Aβ and tau levels in plasma, cerebrospinal fluid (CSF) and brain positron emission tomography (PET) in non‐demented populations [12]. Poor sleep quality was statistically significantly associated with higher Aβ burden in PET imaging, but not with tau. Sleep quality was not associated with plasma concentrations of most biomarkers, although a small but statistically significant correlation with Aβ42 was observed.

The present study addresses the limited evidence on potential associations between plasma Aβ and tau biomarkers and polysomnographic measures of macro‐ and microstructure of sleep in non‐demented individuals. The objectives are to examine: (1) associations between evening and morning plasma AD biomarkers levels and subjective and objective sleep measures, and (2) relationships between sleep parameters and overnight changes in AD biomarker concentrations.

## 2 Methods

### 2.1 Participants

Data presented here is part of a randomized double‐blind, sham‐controlled, cross‐over study investigating possible effects of low frequency magnetic fields on sleep, sleep related changes in the concentration of AD biomarkers and sleep dependent memory consolidation in adults aged 55 to 75 years. Participants underwent a comprehensive medical screening, did not suffer from any sleep disorder, physical or mental illness, and were cognitively unimpaired. Further details on inclusion and exclusion criteria are described in Table S1.

Participants were paid for their participation. The protocol for the study has received prior approval by the ethic committee of the Charité (EA1/036/22) and was registered at the German Clinical Trials Register (DRKS00029254). The course of an experimental night is shown in Figure 1.

**FIGURE 1:**
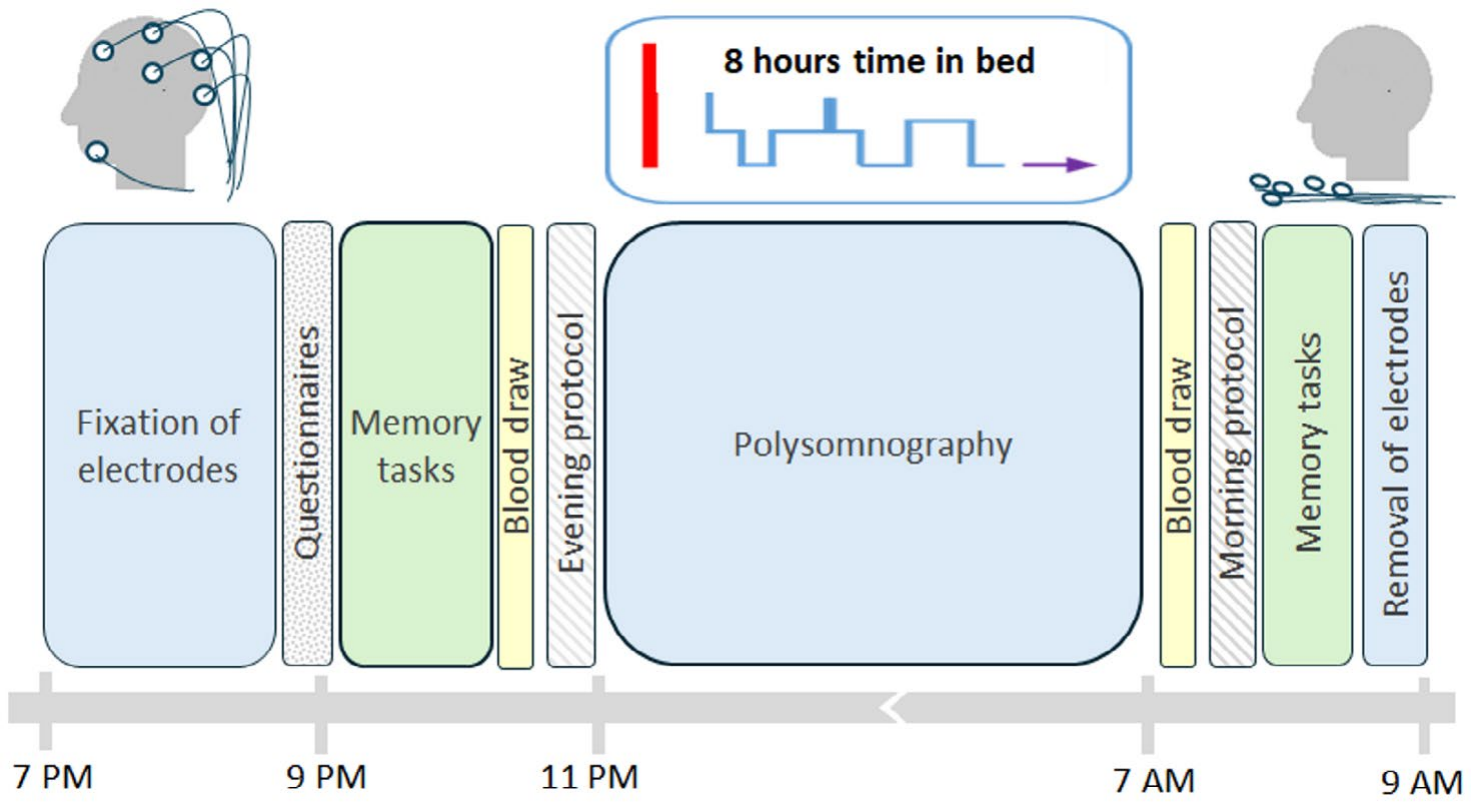
Timeline of the sleep laboratory nights: Included participants came to the laboratory in the evening, and electrodes were fixed according to the standard criteria of the American Academy of Sleep Medicine. After filling in questionnaires on daytime events (e.g., meals, activities; mental state) participants were trained on two different memory tasks. Polysomnography was recorded during an eight‐hour sleep opportunity (= time in bed). Before bedtime and after awakening, blood samples for biomarkers of Alzheimer's disease were collected, and evening and morning protocols, respectively, were filled in by participants. After morning retrieval of the memory tasks, the electrodes were removed and participants left the laboratory.

### 2.2 Sleep Data

A polysomnographically recorded adaptation and screening night was followed by three experimental nights, each 1 week apart (details in Methods S1). Only data from non‐exposed sleep was considered here. In total, 25 outcome parameters addressing the macrostructure of sleep (i.e., sleep continuity, sleep latencies, distribution of sleep stages, awakenings and stage shifts) were considered in this work (Table S2). Furthermore, sleep spindles and sleep stage specific EEG‐power were considered as aspects of the microstructure of sleep.

Based on their known topographical distribution, slow spindles (11–13 Hz) were analyzed frontally and fast spindles (13–16 Hz) parietally [13, 14]. Mean spindle duration, amplitude, frequency, and density (spindles per 30‐s epoch) were calculated for slow spindles at F3‐A2 and F4‐A1 and for fast spindles at P3‐A2 and P4‐A1.

Following the guidelines of the International Pharmaco‐EEG Society (IPEG) [15], power spectra were calculated from 0.5 to 19.75 Hz at 0.25‐Hz resolution and summarized into the following frequency bands (average power in μV^2^): delta (0.50–3.75 Hz), theta (4.00–7.75 Hz), alpha (8.00–11.75 Hz), beta1 (12.00–15.75 Hz), and beta2 (16.00–19.75 Hz), with additional analyzes of spindle frequency ranges per criteria of the American Academy of Sleep Medicine [16] (wider sleep spindle frequency range: 11.00 to 16.00 Hz; narrower sleep spindle frequency range: 12.00 to 14.00 Hz). Mean spectral power density was computed for all 19 EEG derivations separately by sleep stage and for combined NREM sleep stages (N1‐N3).

Standardized protocols [17] were used for the assessment of the subjective sleep latency, the subjective total sleep time in minutes, the subjective duration of wake in minutes, and the estimated number of awakenings during the night. To investigate whether AD biomarkers affect the subjective perception of sleep and its restorative functions, correlations were calculated between the evening, morning and overnight changes in plasma concentrations and subjective measures.

### 2.3 Assessment of AD Biomarkers

Blood samples were collected at predefined time points before and after each study night, via peripheral venipuncture using a Vacutainer blood collection system. Sampling occurred at participant's usual bedtime and within the first hour after awakening. At each time point, two tubes (4.5 mL of lithium heparin, 5 mL potassium EDTA; BD Biosciences) were collected via two venipunctures (< 10 mL per draw).

AD associated biomarkers—beta‐amyloid (Aβ40, Aβ42 and their ratio) and tau proteins (total tau, p‐tau181, p‐tau217) were quantified using SIMOA technology with validated assays (see Supporting Information S1). Due to technical issues, the number of participants with available AD biomarkers analyzes ranged between 35 and 40. Findings obtained in this experimental setting do not allow conclusions regarding the presence of dementia or the individual risk of developing dementia.

### 2.4 Statistical Methods

Pearson's correlation coefficients were used to examine associations between evening AD biomarker levels (prior to nights without intervention) and sleep macro‐ and microstructure during the following night, as well as between sleep parameters and morning levels and overnight changes in plasma AD biomarkers. With a sample size of *n* = 40 correlation coefficients of approximately |r| ≥ 0.31 can be detected to deviate significantly from 0 at a significance level of *p* < 0.05, and correlation coefficients of |*r*| ≥ 0.40 can be detected at the 0.01 significance level. For a sample size of *n* = 35, the corresponding correlation coefficients are |*r*| ≥ 0.34 for the 0.05 significance level and |*r*| ≥ 0.43. Because the analyzes were exploratory, no adjustments for multiple comparisons were performed. This approach was chosen to avoid an undue increase in Type II error. The findings were therefore interpreted accordingly. To investigate whether AD biomarker levels were associated with sleep outcomes, while accounting for potential confounding factors, multiple linear regression models were applied to all significant correlations. For analyzes of evening AD biomarkers, the sleep outcome parameters (subjective and objective sleep measures, spindle parameter, and EEG power) were specified as dependent variable, with age, sex, and body mass index (BMI) included as covariates. For models examining AD biomarkers from morning samples and overnight changes, evening AD biomarkers were additionally included as potential confounders. All statistical analyzes were performed using SAS 9.4.

## 3 Results

### 3.1 Sample Characteristics

The sample consisted of 40 adults (20 women, 20 males), aged between 55 and 75 years. Nine of the 40 participants had pre‐existing medical conditions; all conditions were well controlled under stable medical treatment. Potential confounding medication use was considered unlikely. The sample characteristics are summarized in Table 1.

**TABLE 1:** Sample characteristics for total group and men and women separately.

Variable	Total (*n* = 40)	Men (*n* = 20)	Women (*n* = 20)	*p*
	Mean ± SD	Mean ± SD	Mean ± SD	
Age [years]	63.9 ± 5.6	65.0 ± 5.7	62.9 ± 5.6	0.2560^a^
PSQI	3.1 ± 1.7	2.8 ± 1.7	3.4 ± 1.6	0.1880^b^
ESS	5.0 ± 2.8	5.2 ± 2.9	4.7 ± 2.7	0.7242^b^
PHQ‐9	1.7 ± 1.8	1.1 ± 1.4	2.3 ± 2.0	0.0416^b^
MoCA	27.8 ± 1.3	27.8 ± 1.4	27.7 ± 1.2	0.9003^b^
Sleep latency [min]	14.6 ± 11.1	11.8 ± 7.9	17.5 ± 13.5	0.1445^b^
Sleep efficiency [%]	83.1 ± 6.5	82.2 ± 6.5	83.6 ± 6.5	0.5200^b^
AHI [per h sleep]	6.7 ± 4.4	7.0 ± 4.6	6.3 ± 4.3	0.7269^b^
PLMAI [per h sleep]	3.8 ± 5.2	3.6 ± 4.8	4.1 ± 5.7	0.1875^b^

*Note:* Total scores of screening questionnaires are presented, the sleep data originate from the screening night. All questionnaire scores were within the non‐clinical range.

Abbreviations: AHI, apnea‐hypopnea index; ESS, Epworth Sleepiness Scale; MoCA, Montreal Cognitive Assessment test; PHQ‐9, Patient Health Questionnaire Depression module; PLMAI, periodic limb movement arousal index; PSQI, Pittsburgh Sleep Quality Index.

^a^ *t*‐test for independent samples.

^b^ Wilcoxon two‐sample test.

### 3.2 Subjective Sleep Quality

At a subjective level, longer perceived wake duration was associated with higher t‐tau levels in the morning and a larger overnight increase, while an overnight increase in Aβ42 and a higher Aβ42/Aβ40 ratio in the morning was associated with less perceived wake during the night (Table S3). These associations remained statistically significant when age, sex, BMI, and the AD biomarker concentration in the evening were controlled (see Table S19). With higher levels of Aβ42 in the evening and in the morning as well as higher Aβ40 in the morning perceived sleep latency was longer. These associations, however, were not statistically significant any longer when possible confounders were controlled (Table S19). Overall, wake duration showed a greater number of statistically significant associations with Aβ biomarkers than with tau biomarkers.

### 3.3 Macrostructure—Sleep Architecture

With regard to sleep stages, statistically significant correlations were observed between tau parameters and sleep stage N3. Higher plasma concentrations of t‐tau and p‐tau181 in the evening were associated with less stage N3 sleep (min and % TST; Table S4). For t‐tau, the statistically significant association persists when age, sex, and BMI are controlled, while for p‐tau181 it did not (Table S20). In all models, sex was a statistically significant predictor of stage N3 sleep. An association was also observed between t‐tau levels in the morning and sleep stage N3 (Table S5). In the statistical model which controlled for age, sex, BMI, and the concentration of t‐tau in the evening, stage N3 sleep was not a significant predictor of t‐tau morning concentrations. The only other statistically significant association between AD biomarkers and sleep stages was observed between Aβ40 levels in the morning and the percentage of stage N2: the higher the Aβ40 level, the lower the amount of stage N2 sleep. In the model controlling for confounders, only the Aβ40 level in the evening was a statistically significant predictor of the corresponding level in the morning.

The number of stage shifts was statistically significantly associated with the ratio of Aβ42/40 in the evening and in the morning, but not with their overnight changes (Table S6). The lower the ratio of Aβ42/40, the higher the number (absolute and per hour TST) of stage shifts. When age, sex, and BMI are controlled, evening levels of the ratio of Aβ42/40 had no independent effect on the number of stage shifts the following night. The morning ratio of Aβ42/40 was statistically significantly affected by the evening ratio; stage shifts failed to reach statistical significance (*p* = 0.0605 and *p* = 0.0629, respectively, see Table S20).

The number of stage shifts was also statistically significantly associated with the t‐tau concentration in the morning with lower t‐tau concentrations being associated with a higher number of stage shifts. When confounders are considered, the number of stage shifts still was an independent statistically significant predictor of t‐tau morning levels besides the corresponding levels in the evening. With regard to overnight changes in the concentration of AD biomarkers, statistically significant correlations have been observed for p‐tau181, t‐tau, and Aβ42. In subjects with overnight increases in Aβ42, the number of stage shifts was higher than in those with a decrease in Aβ42. For the overnight changes in t‐tau, the associations with the number of stage shifts remained statistically significant, while for the other AD biomarker, the regression coefficient failed to reach statistical significance; the significance level was *p* < 0.1 (Table S20).

The number of awakenings during the night was neither associated with plasma concentrations of AD markers in the evening and in the morning, nor with their overnight changes. The only exception is a positive correlation of the overnight change in Aβ40, which reached statistical significance for the absolute number of awakenings. In the model considering confounders, the significance of the regression coefficient was *p* = 0.0514 (Table S20).

The objective wake parameters during the night show statistically significant correlations between t‐tau concentrations in the morning and their overnight changes, as well as with the overnight changes in p‐tau271. More wake is associated with higher t‐tau levels. All statistically significant associations persist in the models considering age, sex, BMI, and the evening levels of the AD biomarker as confounders (Table S20). For the morning levels of t‐tau and the overnight change of p‐tau217, the evening level of the corresponding AD biomarker were also statistically significant predictors.

### 3.4 Microstructure—Sleep Spindles

The amplitude and the frequency of the considered sleep spindles show only isolated associations with the concentration of AD biomarkers (Table 2; Tables S7–S15). With one exception (frequency of spindles at F3 during stage N2 and evening p‐tau181 levels), only Aβ biomarkers were affected. The amplitude of sleep spindles at F4 during N2 and NREM decreased with increased levels of Aβ42 in the evening. The statistically significant association persists when age, sex, and BMI are controlled for (Tables S21 and S23). Furthermore, sex is a statistically significant predictor of the amplitude of sleep spindles; females have larger amplitudes than males. The same relation was observed between the amplitude of sleep spindles at F4 during stage N3 and the morning level of Aβ42. Again, the significant association persists when confounders including the evening level of Aβ42 are controlled. Overnight changes in the concentrations of AD biomarkers were neither statistically significantly correlated with the amplitude nor with the frequency of sleep spindles.

**TABLE 2:** Statistically significant correlations between spindle amplitude and spindle frequency in stages N2, N3 and NREM sleep with AD biomarker concentrations collected in the evening and in the morning, and their overnight changes at four different electrode sites (F3, F4 = frontal; P3, P4 = parietal).

	Spindle amplitude												Spindle frequency											
	F3	F4	P3	P4	F3	F4	P3	P4	F3	F4	P3	P4	F3	F4	P3	P4	F3	F4	P3	P4	F3	F4	P3	P4
	Evening collection				Morning collection				Overnight change				Evening collection				Morning collection				Overnight change			
N2																								
p‐tau217																								
p‐tau181													*											
t‐tau																								
Aß42		*														*				*				
Aß40																								
Aß42/40																*								
N3																								
p‐tau217																								
p‐tau181																								
t‐tau																								
Aß42						*																		
Aß40																								
Aß42/40																								
NREM																								
p‐tau217																								
p‐tau181																								
t‐tau																								
Aß42		*														*			*	*				
Aß40																								
Aß42/40															*	*								

*Note:* Blue indicates negative correlations, pink indicates positive correlations.

* *p* < 0.05.

The frequency of parietal sleep spindles was partly positively correlated with Aβ42 concentrations (statistically significantly at P4 during stage N2 with Aβ42 levels in the morning and at P3 and P4 during NREM, as well as with the Aβ42 in the evening). For the morning levels of Aβ42 the statistically significant impact of the frequency at parietal sites persists when the confounders are considered (Tables S21 and S23), while the evening concentrations of this biomarker failed to reach statistical significance in the model with age, sex, and BMI as confounders with significance values of *p* < 0.07. Furthermore, a higher Aβ42/40 ratio in the evening was associated with a higher frequency of sleep spindles at P4 during stage N2 and NREM. This holds true for P3 during NREM as well. All these associations were statistically significant when age, sex, and BMI were controlled (Tables S21 and S23).

For the duration and the density of frontal sleep spindles, another picture emerges (Table 3, Tables S7–S15): Both were consistently negatively correlated with the concentration of Aβ42 and Aβ40 across all sleep stages N2, N3 as well as NREM sleep. This means that a higher concentration of these biomarkers was associated with a shorter duration and a lower density of frontal sleep spindles. With very few exceptions in stage N3, where significance levels reduced to values between *p* = 0.05 and *p* = 0.010, these associations remained statistically significant in the statistical models in which age, sex, and BMI are controlled. The significant correlations observed for the length and duration of sleep spindles and Aβ42 and Aβ40 morning levels are due to a statistically significant effect of the evening values. Parameters of frontal spindles did not significantly affect the morning levels of these two biomarkers in stage N2 and NREM sleep.

**TABLE 3:** Statistically significant correlations between spindle duration and spindle density in stages N2, N3 and NREM sleep with AD biomarker concentrations collected in the evening and in the morning, and their overnight changes at four different electrode sites (F3, F4 = frontal; P3, P4 = parietal).

	Spindle duration												Spindle density											
	F3	F4	P3	P4	F3	F4	P3	P4	F3	F4	P3	P4	F3	F4	P3	P4	F3	F4	P3	P4	F3	F4	P3	P4
	Evening collection				Morning collection				Overnight change				Evening collection				Morning collection				Overnight change			
N2																								
p‐tau217																								
p‐tau181																								
t‐tau																								
Aß42	***	***			***	***							**	***			***	***						
Aß40	**	**	*	*	**	**							***	**	*	*	***	**						
Aß42/40																								
N3																								
p‐tau217																								
p‐tau181									*	*											*			
t‐tau												*								*				
Aß42	***	**			***	**							***	***			***	***						
Aß40	*	*			*								*	**			*	*						
Aß42/40																								
NREM																								
p‐tau217																								
p‐tau181									*												*			
t‐tau																								
Aß42	***	***			***	***							***	***			***	***						
Aß40	**	**	*	*	**	**							**	**			**	**						
Aß42/40																								

*Note:* Blue indicates negative correlations, pink indicates positive correlations.

* *p* < 0.05.

** *p* < 0.01.

*** *p* < 0.001.

For parietal spindles, results are not consistent. During stage N2, a negative association was also observed between evening Aβ40 levels and duration and density of parietal spindles. The negative correlation between duration of parietal spindles at P3 and evening Aβ40 levels was also seen during NREM sleep. The statistically significant correlations between Aβ40 levels in the evening and length and density of parietal sleep spindles during sleep stage N2 and NREM sleep did not remain statistically significant when age, sex, and BMI were controlled (Tables S21 and S23). An isolated finding was a statistically significant correlation between the concentration of t‐tau in the morning and density of sleep spindles in N3 at P4 (Table S22). This statistically significant association persisted in the model with confounders, in which the evening level was also a significant predictor.

Statistically significant associations between overnight changes in the concentration of AD biomarkers and the duration and density of sleep spindles have exclusively been observed for tau markers. At frontal sites, a greater change in p‐tau181 was associated with longer and more sleep spindles at F3 during stage N3 and NREM sleep, and at F4 with sleep spindle duration during N3. Finally, a more pronounced overnight change in t‐tau was associated with longer sleep spindles at P4 during stage N3. None of these associations remained statistically significant when confounders were controlled (Tables S21–S23).

### 3.5 Microstructure—EEG Power Spectra

Significant correlations between tau protein concentrations and EEG power spectra were observed only sporadically, similar to sleep spindles (summaries in Tables S16–S18 in Supporting Information S1). This holds for morning and evening concentrations, their overnight changes, and all sleep stages. The only exception was the evening plasma concentration of p‐tau181—and to a lesser extent p‐tau217—which showed a significant negative correlation with frontal theta power during REM sleep (stage R in Table S16). Higher evening tau levels were associated with lower theta power during REM sleep in the subsequent night. Analyzes with confounders indicate that sex is a significant predictor in these cases, while the tau parameter is not when sex is controlled (Table S36).

In contrast, evening and morning Aβ concentrations showed significant negative correlations with EEG spectral power in the alpha and beta bands and within spindle frequency ranges (Tables S16 and S17). These associations occurred mainly at frontal sites, but also at some central and parietal electrodes. Higher Aβ levels in the evening and morning were consistently associated with lower EEG power in these bands.

For evening Aβ concentrations, this pattern appeared across all sleep stages (example of Aβ40 and N3 in Figure 2A) and the entire night, except during REM sleep, whereas significant correlations with morning Aβ levels were primarily observed during stage N3 (example of Aβ40 and N3 in Figure 2B). In addition, during stage N2 and NREM sleep, spindle‐band power—mainly at frontal sites—showed several significant correlations with morning Aβ42 concentrations. While sex is a statistically significant predictor for the alpha and beta1 as well as the power in the spindle frequency ranges, the Aβ level remained a significant predictor in approximately 50% of the models for the whole night and stage N2. In the other 50%, the significance level of the Aβ level increased to values of usually *p* < 0.01. For stage N1, evening Aβ40 levels remained a statistically significant predictor of alpha and spindle frequency ranges together with sex. For stages N3 and NREM, a significant predictor is Aβ42 together with sex, while Aβ40 seems to be less relevant than Aβ42 (Tables S24–S37). In all sleep stages, the statistically significant correlations between Aβ morning levels and EEG power are mainly due to the evening levels of the corresponding AD biomarker. There are very few exceptions (see e.g., stage N3, Table S32) where EEG power also remained a statistically significant predictor.

**FIGURE 2:**
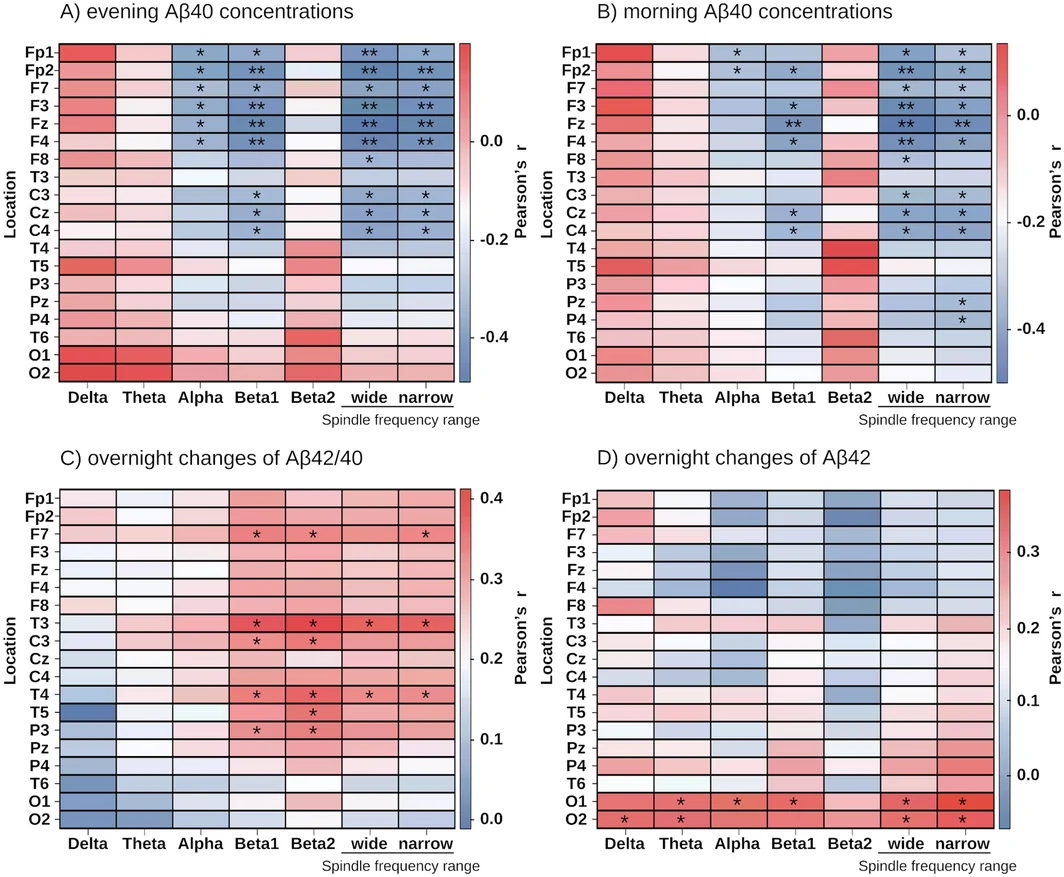
Heatmap of correlations between (A) evening plasma concentration of Aβ40 and EEG power in all considered frequency bands during stage N3; (B) morning plasma concentration of Aβ40 and EEG power in all considered frequency bands during stage N3; (C) overnight changes of the ratio of beta amyloid proteins (Aβ42/40) and EEG power in all considered frequency bands during stage N2; (D) the overnight changes of the plasma concentration of Aβ42 and EEG power in all considered frequency bands during stage N3; * *p* < 0.05, **p < 0.01.

The ratio of evening Aβ concentrations showed almost no association with EEG power, apart from two isolated correlations with theta power at the occipital site O2 during stages N1 and R. In both cases, the ratio of Aβ42/40 remained a statistically significant predictor besides sex, when confounders are controlled (Tables S36 and S26).

In contrast, the morning Aβ42/40 ratio was significantly correlated with theta power during stages N1, R and NREM sleep, and across the entire night: a higher ratio (i.e., relatively higher Aβ42) was associated with lower theta power. In the models which controlled for confounders, in most cases the ratio observed in the evening was the only statistically significant predictor (Tables S25, S26 and S35).

Regarding overnight biomarker changes, notable associations emerged during stages N2 and N3. During stage N2, higher beta and spindle‐band power were associated with a larger overnight increase in the Aβ42/40 ratio (Figure 2C). During stage N3, overnight changes in Aβ42 showed positive correlations with power across all frequency bands except beta2, primarily at occipital sites (Figure 2D).

## 4 Discussion

The present study investigated the associations between AD plasma biomarkers and self‐rated sleep quality as well as objective sleep macro‐ and microstructure in cognitively unimpaired adults aged between 55 and 75 years. To our knowledge, this is the first study to directly assess (1) effects of evening plasma AD biomarker levels on subsequent sleep, (2) associations between sleep and morning plasma AD biomarkers, and (3) relationships between overnight changes in AD biomarker levels and sleep measured with the gold standard polysomnography.

With regard to findings in subjective sleep quality, perceived sleep latency increased with higher Aβ42 (evening and morning) and Aβ40 (morning), but this did not hold true for the polysomnographically measured sleep latency. In contrast, subjective wake duration aligned with objective measures: higher morning t‐tau were associated with more subjective wake duration and with objective wake after sleep onset and wake during the sleep period. Overnight changes in p‐tau showed a negative correlation with both subjective and objective wake time.

Higher evening t‐tau and p‐tau181 were associated with reduced stage N3 sleep, suggesting that elevated tau leads to poorer slow‐wave sleep. Indicators of disturbed sleep, such as increased stage shifting, were associated with lower evening and morning Aβ42/Aβ40 ratios. However, overnight biomarker changes indicated that greater tau decreases were associated with fewer stage shifts, while increases in Aβ42 and Aβ40 were linked to more disturbances and awakenings. However, the experimental design, which evaluated changes across a single night, does not allow conclusions to be drawn regarding the temporal or causal direction of the observed changes.

Finally, sleep spindle characteristics‐ especially duration and density‐ were associated with AD biomarkers, with stronger and more consistent correlations for Aβ than tau, varying by sleep stage and timing of measurement. These findings are largely consistent with previous studies, which also indicate a close relationship between sleep microstructure and neuropathological processes in AD, suggesting spindle features as potential non‐invasive biomarkers [18, 19, 20].

Weng et al. (2020) [20] reviewed 12 studies on sleep spindle microstructure in AD and related conditions, finding disruptions in density, amplitude, duration, frequency, and coupling with slow oscillations. Spindle density appeared more sensitive to AD than frequency of spindles, and fast spindles were more sensitive than slow spindles. Liu et al. (2020) [19] similarly reported reduced spindle density, amplitude, and duration in AD and mild cognitive impairment (MCI), with a graded pattern (controls > MCI > AD). Gorgoni et al. (2016) [18] observed reduced parietal fast spindle density in MCI and AD that was linked to cognitive impairment, suggesting spindle changes may represent an early marker of AD‐related neurodegeneration, though overall evidence remains limited.

In a study on cognitively unimpaired elderly individuals, a decrease of spindle density during N2 was associated with a higher burden of CSF t‐tau, p‐tau, and Aβ42 levels, and spindle duration was negatively correlated with t‐tau concentrations [21]. In the present study, spindle duration and density in N2, N3, and NREM were also negatively correlated to both evening and morning Aβ42 levels. Greater overnight changes in p‐tau181 were associated with increased spindle duration and density in N3 and NREM at frontal electrode sites. T‐tau showed increased changes in overnight levels with increased spindle duration and increased morning level, both at P4 during N3 only.

Correlations between tau protein levels and EEG power spectra were largely inconsistent in the present study, with the only notable finding being a negative association between evening p‐tau181 and frontal theta power during REM sleep. In contrast, evening and morning Aβ levels showed more consistent negative correlations with alpha, beta, and spindle‐band power, mainly at frontal sites. To our knowledge, the only other study which investigated EEG power during sleep in relation to the concentration of AD biomarker in 60 healthy individuals was Rosenblum et al. [22]. They found higher evening plasma Aβ42 and Aβ40 levels, with positive associations between Aβ and slow‐wave sleep as well as EEG power in the 0.3–4 Hz and 30–48 Hz ranges. Slow oscillation power also predicted higher future Aβ40 levels. In comparison, our findings were more limited, with regard to the delta frequency range (2–4 Hz) showing only localized correlations at O2 with overnight Aβ42 during stage N3. These differences may partly reflect methodological and sample characteristics. Rosenblum et al. collected blood every 20 min throughout sleep (23:00–07:00) and examined lagged associations with subsequent Aβ levels, whereas in the present study samples were only drawn before and after night sleep. In addition, their participants were younger and had a broader age range (20–68 vs. 55–75 years).

Overnight sleep, particularly slow wave sleep, acts as a critical, high‐efficiency cleaning period for the brain, utilizing the glymphatic system to clear neurotoxic Alzheimer's‐linked proteins, Amyloid‐beta (Aβ) and tau, from brain tissue [23]. Sleep deprivation impairs this clearance mechanism, leading to higher levels of Aβ and tau, which can accelerate neurodegeneration [10, 24]. Before overnight changes in plasma biomarkers are interpreted in terms of central clearance, several methodological constraints have to be considered, and these apply not only to the Aβ42/Aβ40 ratio but to the absolute overnight changes in all markers examined here. Evening‐to‐morning differences in peripheral concentrations represent the net result of central efflux, peripheral production, and hepatic and renal elimination, superimposed on posture‐ and hydration‐related changes in plasma volume. The transition between upright and recumbent posture alone shifts measured plasma protein concentrations, with the magnitude of the effect increasing with molecular size [25]. In addition, plasma AD biomarkers follow a diurnal profile. In a 24‐h protocol in older adults, within‐day variation reached 14.0% for Aβ40, 15.3% for Aβ42 and 15.8% for p‐tau217, with amyloid‐β peaking during nocturnal sleep and reaching its lowest values upon awakening [26]. Smaller but consistent evening‐to‐morning differences in plasma Aβ42 (4.8%) and Aβ40 (4.2%) have been reported in a clinical cohort [27]. An evening‐to‐morning decrease in absolute Aβ concentrations is therefore, at least in part, the expected physiological pattern rather than an index of nocturnal brain clearance. Notably, in both studies the Aβ42/Aβ40 ratio was considerably more stable (4.6% within‐day variation; no significant morning‐evening difference), consistent with the assumption that two peptides affected in parallel largely cancel non‐specific concentration shifts. The ratio is thus less vulnerable to volume effects than the absolute values, but remains sensitive to differential elimination kinetics and matrix effects and does not by itself imply an observed change is related to changes in the brain. As the present design included neither paired CSF sampling nor amyloid or tau PET, nor a direct physiological measure of glymphatic and meningeal lymphatic function, approaches that have recently been combined with plasma sampling to substantiate a central origin of overnight biomarker changes [23, 28, 29], the associations between sleep parameters and overnight biomarker changes reported here should be regarded as correlative and hypothesis‐generating rather than evidence of altered central clearance.

Possible effects of clearance were also observed in the present study with regard to t‐tau measured in the morning, which was negatively correlated with the amount and percentage of stage N3 sleep of the previous night, whereas increased wakefulness was correlated with a higher concentration of t‐tau in the morning. In contrast to recent findings, EEG delta power in all stages was not associated with any of the AD biomarkers. Increased theta and reduced beta power have been linked to glymphatic functions [23, 24, 28]. In the present study, increased theta power during the entire night was related to a lower Aβ42/Aβ40 ratio in the morning, which would fit other study results, interpreting similar findings as a result of glymphatic clearance during sleep [23, 28]. The observed correlation of an increase in beta power with lower Aβ40 and Aβ42 levels in the morning was only statistically significant during N3; no other stages were affected. Therefore, an assumed negative impact on clearance might not have been strong enough.

Sleep disruption may increase Aβ accumulation, and amyloid deposition may in turn impair sleep, creating a feed‐forward cycle between sleep disruption and AD pathology. Sleep and Alzheimer's pathology interact bidirectionally: sleep disruption increases Aβ and tau dynamics, whereas the accumulation of these proteins impairs slow‐wave sleep and may reduce glymphatic clearance, potentially creating a self‐reinforcing cycle [4, 10]. Interestingly, the negative effect of disturbed sleep, represented by the number and percentage of stage shifts in the present study, was negatively correlated with morning levels and overnight changes in t‐tau and in morning Aβ42/40 ratio. It might be speculated that in the healthy and good sleeper sample of the present study the number of stage shifts was not high enough to impair sleep. This hypothesis is supported by a similar observation of SWS arousals leading to higher Aβ42 plasma levels in patients with MCI symptoms but not in healthy individuals [30].

Dagum et al. [23] studied 33 cognitively healthy older adults (*n* = 38 before exclusion of five participants: mean age +/− standard deviation, SD: 59.8 ± 4.5 years) under two experimental conditions, a normal overnight sleep and an acute sleep deprivation condition. Plasma biomarkers Aβ40, Aβ42, tau (np‐tau181, np‐tau217, p‐tau181) were analyzed from blood samples collected in the evening and in the morning. Physiological markers of glymphatic activity were recorded using a device measuring EEG and brain parenchymal resistance to fluid flow. During normal sleep, morning plasma levels of Aβ and tau were higher than after sleep deprivation. Higher EEG delta power during NREM sleep was associated with greater overnight increases in plasma Aβ and tau. The authors interpreted these increases as reflecting effective brain clearance from the brain rather than pathological processes. These effects might also transfer to some part to the findings of the present study, where power in stage N3 sleep was correlated to overnight changes in Aβ (theta power) and Aβ42 (all power spectra) at occipital sites.

## 5 Conclusion

In cognitively unimpaired older adults, plasma AD biomarkers were associated with polysomnographic measures of sleep, with the most consistent relationships observed between amyloid‐β markers and sleep microstructure, particularly sleep spindle duration, spindle density, and spectral EEG power in alpha, beta, and spindle frequency bands. Tau‐related associations were less frequent and mainly involved reduced N3 sleep and measures of nocturnal wakefulness or sleep instability. These findings support the hypothesis that objective sleep microstructure may reflect early AD‐related biological processes before clinical impairment. Although a key strength of this study is the consistent collection of plasma samples at two standardized time points—before PSG in the evening and immediately after sleep in the morning—changes in AD biomarkers over the night should be interpreted with caution for the reasons detailed above, as they may reflect nonspecific shifts in plasma volume or peptide concentration rather than true changes of amyloid dynamics. The same holds true for the absolute overnight changes in all other plasma biomarkers. Given the small sample size, the cross‐sectional and correlational design and the large number of exploratory analyzes, the findings should be interpreted cautiously. Nevertheless, the persistence of the observed associations after adjustment for age, sex, and BMI suggests that they are not readily explained by these potential confounders. Sleep spindle metrics may therefore reflect early AD‐related biological processes, consistent with previous studies linking spindle characteristics and spindle‐slow‐wave coupling to CSF AD biomarkers and cerebral amyloid burden. However, the findings should be considered as hypothesis‐generating and require replication in larger longitudinal cohorts using multimodal AD biomarker assessments and repeated sleep and cognitive testing.

## Author Contributions

C.S., H.D., J.H.‐R, A.B.‐L. and H.D.‐H. contributed to the conception and design of the study. C.S., H.D., J.H.‐R, A.B.‐L., V.G. and H.D.‐H. contributed to the acquisition or analysis of data. C.S., H.D. and H.D.‐H. contributed to drafting a significant portion the manuscript or figures.

## Funding

This work was supported by the Federal Office for Radiation Protection [Bundesamt für Strahlenschutz, BfS], 38226 Salzgitter, Germany (Grant number 3621SNA403).

## Conflicts of Interest

The authors declare no conflicts of interest.

## Supporting information


**Table S1:** Inclusion and exclusion criteria.
**Table S2:** Macrostructure outcome parameters.
**Table S3:** Correlations (Pearson *r*) of subjective assessments of sleep quality with the plasma concentration of six AD‐biomarkers in the (A) evening, in (B) morning and with (C) overnight changes. Statistically significant correlations are shown in bold.
**Table S4:** Correlations (Pearson *r*) of macrostructure of sleep with the plasma concentration of six AD‐biomarkers in the evening. Definitions of the abbreviations can be found in Table S2. Statistically significant correlations are shown in bold.
**Table S5:** Correlations (Pearson *r*) of macrostructure of sleep with the plasma concentration of six AD‐biomarkers in the morning. Definitions of the abbreviations can be found in Table S2. Statistically significant correlations are shown in bold.
**Table S6:** Correlations (Pearson *r*) of macrostructure of sleep with overnight changes in plasma concentration of six AD‐biomarkers. Definitions of the abbreviations can be found in Table S2. Statistically significant correlations are shown in bold.
**Table S7:** Correlations (Pearson *r*) of sleep spindle parameters (length = duration, amplitude, frequency, density) in stage N2 at four electrode sites (F3, F4, P3, P4) with plasma concentration of six AD‐biomarkers collected in the evening. Statistically significant correlations are shown in bold.
**Table S8:** Correlations (Pearson *r*) of sleep spindle parameters (length = duration, amplitude, frequency, density) in stage N3 at four electrode sites (F3, F4, P3, P4) with plasma concentration of six AD‐biomarkers collected in the evening. Statistically significant correlations are shown in bold.
**Table S9:** Correlations (Pearson *r*) of sleep spindle parameters (length = duration, amplitude, frequency, density) in stage NREM at four electrode sites (F3, F4, P3, P4) with plasma concentration of six AD‐biomarkers collected in the evening. Statistically significant correlations are shown in bold.
**Table S10:** Correlations (Pearson *r*) of sleep spindle parameters (length = duration, amplitude, frequency, density) in stage N2 at four electrode sites (F3, F4, P3, P4) with plasma concentration of six AD‐biomarkers collected in the morning. Statistically significant correlations are shown in bold.
**Table S11:** Correlations (Pearson *r*) of sleep spindle parameters (length = duration, amplitude, frequency, density) in stage N3 at four electrode sites (F3, F4, P3, P4) with plasma concentration of six AD‐biomarkers collected in the morning. Statistically significant correlations are shown in bold.
**Table S12:** Correlations (Pearson *r*) of sleep spindle parameters (length = duration, amplitude, frequency, density) in stage NREM at four electrode sites (F3, F4, P3, P4) with plasma concentration of six AD‐biomarkers collected in the morning. Statistically significant correlations are shown in bold.
**Table S13:** Correlations (Pearson *r*) of sleep spindle parameters (length = duration, amplitude, frequency, density) in stage N2 at four electrode sites (F3, F4, P3, P4) with overnight changes in plasma concentration of six AD‐biomarkers. Statistically significant correlations are shown in bold.
**Table S14:** Correlations (Pearson *r*) of sleep spindle parameters (length = duration, amplitude, frequency, density) in stage N3 at four electrode sites (F3, F4, P3, P4) with overnight changes in plasma concentration of six AD‐biomarkers. Statistically significant correlations are shown in bold.
**Table S15:** Correlations (Pearson *r*) of sleep spindle parameters (length = duration, amplitude, frequency, density) in NREM at four electrode sites (F3, F4, P3, P4) with overnight changes in plasma concentration of six AD‐biomarkers. Statistically significant correlations are shown in bold.
**Table S16:** Statistically significant correlations between power spectra of seven different frequency bands at 19 different electrode sites and AD biomarker concentrations collected in the evening are shown for (A) entire night, (B) stage N1, (C) stage N2, (D) stage N3, (E) NREM sleep and (F) stage R. Locations of statistically significant correlations are presented. Positive correlations are shown in bold, negative correlations in regular font.
**Table S17:** Statistically significant correlations between power spectra of seven different frequency bands at 19 different electrode sites and AD biomarker concentrations collected in the morning are shown for (A) entire night, (B) stage N1, (C) stage N2, (D) stage N3, (E) NREM sleep and (F) stage R. Locations of statistically significant correlations are presented. Positive correlations are shown in bold, negative correlations in regular font.
**Table S18:** Statistically significant correlations between power spectra of seven different frequency bands at 19 different electrode sites and overnight changes in AD biomarker concentrations are shown for (A) entire night, (B) stage N1, (C) stage N2, (D) stage N3, (E) NREM sleep and (F) stage R. Locations of statistically significant correlations are presented. Positive correlations are shown in bold, negative correlations in regular font.
**Table S19:** Significant Pearson correlations and adjusted linear regression results for subjective sleep quality and AD biomarkers. Regression models were adjusted for evening AD biomarker levels (n.a. for evening biomarker correlations), sex, age, and BMI. Statistically significant results are shown in bold.
**Table S20:** Significant Pearson correlations and adjusted linear regression results for objective sleep quality and AD biomarkers. Regression models were adjusted for evening AD biomarker levels (n.a. for evening biomarker correlations), sex, age, and BMI. Statistically significant results are shown in bold.
**Table S21:** Significant Pearson correlations and adjusted linear regression results for sleep spindle variables in stage N2 and AD biomarkers. Regression models were adjusted for evening AD biomarker levels (n.a. for evening biomarker correlations), sex, age, and BMI. Statistically significant results are shown in bold.
**Table S22:** Significant Pearson correlations and adjusted linear regression results for sleep spindle variables in stage N3 and AD biomarkers. Regression models were adjusted for evening AD biomarker levels (n.a. for evening biomarker correlations), sex, age, and BMI. Statistically significant results are shown in bold.
**Table S23:** Significant Pearson correlations and adjusted linear regression results for sleep spindle variables in stage NREM and AD biomarkers. Regression models were adjusted for evening AD biomarker levels (n.a. for evening biomarker correlations), sex, age, and BMI. Statistically significant results are shown in bold.
**Table S24:** Significant Pearson correlations and adjusted linear regression results for sleep EEG power (whole night, evening AD biomarkers) and AD biomarkers. Regression models were adjusted for evening AD biomarker levels (n.a. for evening biomarker correlations), sex, age, and BMI. Statistically significant results are shown in bold.
**Table S25:** Significant Pearson correlations and adjusted linear regression results for sleep EEG power (whole night, morning AD biomarkers) and AD biomarkers. Regression models were adjusted for evening AD biomarker levels (n.a. for evening biomarker correlations), sex, age, and BMI. Statistically significant results are shown in bold.
**Table S26:** Significant Pearson correlations and adjusted linear regression results for sleep EEG power (Stage N1, evening AD biomarkers) and AD biomarkers. Regression models were adjusted for evening AD biomarker levels (n.a. for evening biomarker correlations), sex, age, and BMI. Statistically significant results are shown in bold.
**Table S27:** Significant Pearson correlations and adjusted linear regression results for sleep EEG power (Stage N1, morning AD biomarkers) and AD biomarkers. Regression models were adjusted for evening AD biomarker levels (n.a. for evening biomarker correlations), sex, age, and BMI. Statistically significant results are shown in bold.
**Table S28:** Significant Pearson correlations and adjusted linear regression results for sleep EEG power (Stage N2, evening AD biomarkers) and AD biomarkers. Regression models were adjusted for evening AD biomarker levels (n.a. for evening biomarker correlations), sex, age, and BMI. Statistically significant results are shown in bold.
**Table S29:** Significant Pearson correlations and adjusted linear regression results for sleep EEG power (Stage N2, morning AD biomarkers) and AD biomarkers. Regression models were adjusted for evening AD biomarker levels (n.a. for evening biomarker correlations), sex, age, and BMI. Statistically significant results are shown in bold.
**Table S30:** Significant Pearson correlations and adjusted linear regression results for sleep EEG power (Stage N2, overnight change of AD biomarkers) and AD biomarkers. Regression models were adjusted for evening AD biomarker levels (n.a. for evening biomarker correlations), sex, age, and BMI. Statistically significant results are shown in bold.
**Table S31:** Significant Pearson correlations and adjusted linear regression results for sleep EEG power (Stage N3, evening AD biomarkers) and AD biomarkers. Regression models were adjusted for evening AD biomarker levels (n.a. for evening biomarker correlations), sex, age, and BMI. Statistically significant results are shown in bold.
**Table S32:** Significant Pearson correlations and adjusted linear regression results for sleep EEG power (Stage N3, morning AD biomarkers) and AD biomarkers. Regression models were adjusted for evening AD biomarker levels (n.a. for evening biomarker correlations), sex, age, and BMI.
**Table S33:** Significant Pearson correlations and adjusted linear regression results for sleep EEG power (Stage N3, overnight change of AD biomarkers) and AD biomarkers. Regression models were adjusted for evening AD biomarker levels (n.a. for evening biomarker correlations), sex, age, and BMI. Statistically significant results are shown in bold.
**Table S34:** Significant Pearson correlations and adjusted linear regression results for sleep EEG power (NREM, evening AD biomarkers) and AD biomarkers. Regression models were adjusted for evening AD biomarker levels (n.a. for evening biomarker correlations), sex, age, and BMI. Statistically significant results are shown in bold.
**Table S35:** Significant Pearson correlations and adjusted linear regression results for sleep EEG power (NREM, morning AD biomarkers) and AD biomarkers. Regression models were adjusted for evening AD biomarker levels (n.a. for evening biomarker correlations), sex, age, and BMI. Statistically significant results are shown in bold.
**Table S36:** Significant Pearson correlations and adjusted linear regression results for sleep EEG power (Stage R, evening AD biomarkers) and AD biomarkers. Regression models were adjusted for evening AD biomarker levels (n.a. for evening biomarker correlations), sex, age, and BMI. Statistically significant results are shown in bold.
**Table S37:** Significant Pearson correlations and adjusted linear regression results for sleep EEG power (Stage R, morning AD biomarkers) and AD biomarkers. Regression models were adjusted for evening AD biomarker levels (n.a. for evening biomarker correlations), sex, age, and BMI. Statistically significant results are shown in bold.
**Table S38:** Significant Pearson correlations and adjusted linear regression results for sleep EEG power (Stage R, overnight change of AD biomarkers) and AD biomarkers. Regression models were adjusted for evening AD biomarker levels (n.a. for evening biomarker correlations), sex, age, and BMI. Statistically significant results are shown in bold.

## Data Availability

Detailed tables with data are available from the authors upon request. Anonymized participant data and supporting documentation will be made available upon reasonable request following publication. Access will be granted to qualified investigators, contingent upon approval by the lead investigator. Requests should be directed to heidi.danker-hopfe@charite.de. Data may be used for any purpose deemed scientifically sound and ethically approved.
