# Supplementary material for: Associations Between Biomarkers of Alzheimer's Disease and Sleep in Cognitively Unimpaired Older Adults
Source: Ann Clin Transl Neurol. 2026 Oct 21:10.1002/acn3.70562. Online ahead of print. doi: 10.1002/acn3.70562 (PMC13685406; doi:10.1002/acn3.70562)
Supplement: Supplementary file 1 [file ACN3-9999-0-s001.docx]

**Supplemental material**

[**Table S1.** Inclusion and exclusion criteria	3](#_Toc240966388)

[**Methods** Supplemental Material	4](#_Toc240966389)

[**Table S2.** Macrostructure outcome parameters	5](#_Toc240966390)

[**Table S3.** Correlations (Pearson r) of subjective assessments of sleep quality with the plasma concentration of six AD-biomarkers in the A) evening, in B) morning and with C) overnight changes.	6](#_Toc240966391)

[**Table S4.** Correlations (Pearson r) of macrostructure of sleep with the plasma concentration of six AD-biomarkers in the evening.	7](#_Toc240966392)

[**Table S5.** Correlations (Pearson r) of macrostructure of sleep with the plasma concentration of six AD-biomarkers in the morning..	9](#_Toc240966393)

[**Table S6.** Correlations (Pearson r) of macrostructure of sleep with overnight changes in plasma concentration of six AD-biomarkers.	11](#_Toc240966394)

[**Table S7.** Correlations (Pearson r) of sleep spindle parameters (length = duration, amplitude, frequency, density) in stage N2 at four electrode sites (F3, F4, P3, P4) with plasma concentration of six AD-biomarkers collected in the evening..	13](#_Toc240966395)

[**Table S8.** Correlations (Pearson r) of sleep spindle parameters (length = duration, amplitude, frequency, density) in stage N3 at four electrode sites (F3, F4, P3, P4) with plasma concentration of six AD-biomarkers collected in the evening.	14](#_Toc240966396)

[**Table S9.** Correlations (Pearson r) of sleep spindle parameters (length = duration, amplitude, frequency, density) in stage NREM at four electrode sites (F3, F4, P3, P4) with plasma concentration of six AD-biomarkers collected in the evening.	15](#_Toc240966397)

[**Table S10.** Correlations (Pearson r) of sleep spindle parameters (length = duration, amplitude, frequency, density) in stage N2 at four electrode sites (F3, F4, P3, P4) with plasma concentration of six AD-biomarkers collected in the morning.	16](#_Toc240966398)

[**Table S11.** Correlations (Pearson r) of sleep spindle parameters (length = duration, amplitude, frequency, density) in stage N3 at four electrode sites (F3, F4, P3, P4) with plasma concentration of six AD-biomarkers collected in the morning.	17](#_Toc240966399)

[**Table S12.** Correlations (Pearson r) of sleep spindle parameters (length = duration, amplitude, frequency, density) in stage NREM at four electrode sites (F3, F4, P3, P4) with plasma concentration of six AD-biomarkers collected in the morning.	18](#_Toc240966400)

[**Table S13** Correlations (Pearson r) of sleep spindle parameters (length = duration, amplitude, frequency, density) in stage N2 at four electrode sites (F3, F4, P3, P4) with overnight changes in plasma concentration of six AD-biomarkers.	19](#_Toc240966401)

[**Table S14.** Correlations (Pearson r) of sleep spindle parameters (length = duration, amplitude, frequency, density) in stage N3 at four electrode sites (F3, F4, P3, P4) with overnight changes in plasma concentration of six AD-biomarkers.	20](#_Toc240966402)

[**Table S15.** Correlations (Pearson r) of sleep spindle parameters (length = duration, amplitude, frequency, density) in NREM at four electrode sites (F3, F4, P3, P4) with overnight changes in plasma concentration of six AD-biomarkers.	21](#_Toc240966403)

[**Table S16.** Statistically significant correlations between power spectra of seven different frequency bands at 19 different electrode sites and AD biomarker concentrations collected in the evening are shown for A) entire night, B) stage N1, C) stage N2, D) stage N3, E) NREM sleep and F) stage R. Locations of statistically significant correlations are presented.	22](#_Toc240966404)

[**Table S17.** Statistically significant correlations between power spectra of seven different frequency bands at 19 different electrode sites and AD biomarker concentrations collected in the morning are shown for A) entire night, B) stage N1, C) stage N2, D) stage N3, E) NREM sleep and F) stage R.	24](#_Toc240966405)

[**Table S18.** Statistically significant correlations between power spectra of seven different frequency bands at 19 different electrode sites and overnight changes in AD biomarker concentrations are shown for A) entire night, B) stage N1, C) stage N2, D) stage N3, E) E) NREM sleep and F) stage R.	26](#_Toc240966406)

[**Table S19.** Significant Pearson correlations and adjusted linear regression results for subjective sleep quality and AD biomarkers.	28](#_Toc240966407)

[**Table S20.** Significant Pearson correlations and adjusted linear regression results for objective sleep quality and AD biomarkers.	29](#_Toc240966408)

[**Table S21.** Significant Pearson correlations and adjusted linear regression results for sleep spindle variables in stage N2 and AD biomarkers.	30](#_Toc240966409)

[**Table S22.** Significant Pearson correlations and adjusted linear regression results for sleep spindle variables in stage N3 and AD biomarkers.	31](#_Toc240966410)

[**Table S23** Significant Pearson correlations and adjusted linear regression results for sleep spindle variables in stage NREM and AD biomarkers.	32](#_Toc240966411)

[**Table S24**. Significant Pearson correlations and adjusted linear regression results for sleep EEG power (whole night, evening AD biomarkers) and AD biomarkers.	33](#_Toc240966412)

[**Table S25**. Significant Pearson correlations and adjusted linear regression results for sleep EEG power (whole night, morning AD biomarkers) and AD biomarkers.	34](#_Toc240966413)

[**Table S26.** Significant Pearson correlations and adjusted linear regression results for sleep EEG power (Stage N1, evening AD biomarkers) and AD biomarkers.	35](#_Toc240966414)

[**Table S27.** Significant Pearson correlations and adjusted linear regression results for sleep EEG power (Stage N1, morning AD biomarkers) and AD biomarkers.	36](#_Toc240966415)

[**Table S28.** Significant Pearson correlations and adjusted linear regression results for sleep EEG power (Stage N2, evening AD biomarkers) and AD biomarkers.	37](#_Toc240966416)

[**Table S29.** Significant Pearson correlations and adjusted linear regression results for sleep EEG power (Stage N2, morning AD biomarkers) and AD biomarkers.	39](#_Toc240966417)

[**Table S30.** Significant Pearson correlations and adjusted linear regression results for sleep EEG power (Stage N2, overnight change of AD biomarkers) and AD biomarkers.	40](#_Toc240966418)

[**Table S31.** Significant Pearson correlations and adjusted linear regression results for sleep EEG power (Stage N3, evening AD biomarkers) and AD biomarkers.	41](#_Toc240966419)

[**Table S32.** Significant Pearson correlations and adjusted linear regression results for sleep EEG power (Stage N3, morning AD biomarkers) and AD biomarkers.	43](#_Toc240966420)

[**Table S33.** Significant Pearson correlations and adjusted linear regression results for sleep EEG power (Stage N3, overnight change of AD biomarkers) and AD biomarkers.	45](#_Toc240966421)

[**Table S34.** Significant Pearson correlations and adjusted linear regression results for sleep EEG power (NREM, evening AD biomarkers) and AD biomarkers.	46](#_Toc240966422)

[**Table S35.** Significant Pearson correlations and adjusted linear regression results for sleep EEG power (NREM, morning AD biomarkers) and AD biomarkers.	48](#_Toc240966423)

[**Table S36.** Significant Pearson correlations and adjusted linear regression results for sleep EEG power (Stage R, evening AD biomarkers) and AD biomarkers.	49](#_Toc240966424)

[**Table S37.** Significant Pearson correlations and adjusted linear regression results for sleep EEG power (Stage R, morning AD biomarkers) and AD biomarkers.	50](#_Toc240966425)

[**Table S38.** Significant Pearson correlations and adjusted linear regression results for sleep EEG power (Stage R, overnight change of AD biomarkers) and AD biomarkers.	51](#_Toc240966426)

# **Table S1. Inclusion and exclusion criteria**

**Inclusion criteria**

• Age between 55 – 75 years

• Current physical and mental health (confirmed by comprehensive neurological, psychiatric and physical examination)

• No or stable internal medication

• For females only: postmenopausal women (last menstrual period one year ago)

• Non-smoker

• Regular sleep-wake cycle (confirmed by sleep diary)

• Mother tongue German or comparable language level

• Right-handedness

**Exclusion criteria**

• Sleep disorder according to the International Classification of Sleep Disorders (ICSD 3)

• Sleep disorder according to the International Classification of Sleep Disorders (ICSD 3)

• Apnoe-hypopnoe index ≥ 15/h (polygraphy or screening night)

• Screning night: sleep latency > 45 min, a sleep efficiency < 70% and a periodic limb movement arousal index ≥ 20/h

• Working on night shift (22:00 to 06:00)

• Use of hypnotics or other medication that may affect sleep

• Excessive consumption of substances that affect the central nervous system (including caffeinated drinks, alcohol, drugs)

• Pittsburgh Sleep Quality Index, PSQI total score ≤ 7

• Epworth Sleepiness Scale, ESS total score < 10

• Patient Health Questionnaire, Depression Module, PHQ-9 total score ≤ 10

• Montreal Cognitive Assessment, MoCA total score < 26

# **Methods Supplemental Material**

*Polysomnography*

Polysomnography was conducted according to the standards of the American Academy of Sleep Medicine (AASM) and recorded for eight hours in all nights.^1^ Nineteen EEG electrodes were placed according to the 10-20 system^2^, besides two mastoid electrodes and one ground electrode (Fpz). The computer-assisted scoring of the macrostructure of sleep (sleep continuity, sleep latencies, sleep stages, awakenings and sleep stage changes) was performed with the Somnolyzer 24x7 according to recommendations of the AASM^1, 3^ with an algorithm approved by the Food and Drug Administration.^4, 5^

*Macrostructure of sleep*

The 25 variables describing the macrostructure of sleep include total sleep time, sleep latency, latency to persistent sleep latencies to stages N2, N3, R, time spent in each sleep stage (minutes and percent of TST), wake after sleep onset, sleep efficiency, number of awakenings and stage shifts (total and per hour TST). Definitions of all macrostructure parameter are presented in Table S2.

*Microstructure of sleep: spindles*

Sleep spindles were analyzed according to Adamczyk et al.^6^ using a continuous-time wavelet transformation with high temporal resolution, which is well suited for detecting spindles due to their characteristic frequency range and morphology. Prior to analysis, muscle artifacts (EMG activity with amplitudes > 5.75 µV in the frequency range 19.8 to 45.5 Hz) and alpha-dominated EEG (alpha/delta ratios > 1.1) segments were removed. Spindles were detected in the 9 to 16 Hz range at 0.1 Hz resolution.

*Microstructure: EEG power*

After artifact removal, artifact-free 4-s EEG segments were used to compute power spectra using the Welch method^7^ with 2-s overlap, and a von-Hann window. Segment spectra were averaged to obtain 30-s epoch spectra (up to 14 segments per epoch).

*Assessment of AD biomarkers*

AD associated biomarkers - beta-amyloid (Aβ40, Aβ42 and their ratio) and tau proteins (total tau, p-tau181, p-tau217) - were quantified using SIMOA^®^ technology with validated assays (Neurology 3-Plex A Advantage Kit [AB40, AB42, Tau], #104618, ALZpath p-Tau 217 Advantage PLUS Kit, #101995, p-Tau 181 Advantage PLUS Reagent Kit, all Quanterix™).

1.	Berry R, Quan S, Abreu A, al. e. The AASM Manual for the Scoring of Sleep and Associated Events: Rules, Terminology and Technical Specifications, Version 2.6. . American Academy of Sleep Medicine, editor. Darien, IL,2020.

2.	Jasper HH. The ten twenty electrode system of the International Federation. Electroencephalography and Clinical Neurophysiology. 1958;10(2):371–5.

3.	Iber C, Ancoli-Israel S, Chesson A, Quan SF, for the American Academy of Sleep Medicine. The AASM Manual for the Scoring of Sleep and Associated Events: rules, Terminology and technical Specifications. 1st ed. ed. Westchester: Illinois: American Academy of Sleep Medicine; 2007.

4.	Anderer P, Moreau A, Woertz M, et al. Computer-assisted sleep classification according to the standard of the American Academy of Sleep Medicine: validation study of the AASM version of the Somnolyzer 24 x 7. Neuropsychobiology. 2010;62(4):250–64.

5.	Anderer P, Ross M, Cerny A, Shaw E. Automated Scoring of Sleep and Associated Events. Adv Exp Med Biol. 2022;1384:107–30.

6.	Adamczyk M, Genzel L, Dresler M, Steiger A, Friess E. Automatic Sleep Spindle Detection and Genetic Influence Estimation Using Continuous Wavelet Transform. Front Hum Neurosci. 2015;9:624.

7.	Welch PD. A Direct Digital Method of Power Spectrum Estimation. IBM Journal of Research and Development. 1961;5(2):141–56.

# **Table S2.** Macrostructure outcome parameters

| **Abbreviation** | **Sleep variable** | **Description / Definition** | **Unit** |
| --- | --- | --- | --- |
| **Sleep continuity** | | | |
| TST | Total Sleep Time | Time spent asleep in stages N1, N2, N3, and R | min |
| TSP | Total Sleep Period | Time from first to last sleep epoch (including wake) | min |
| WASO | Wake After Sleep Onset | Wake from first epoch of sleep until “lights on” | min |
| WTSP | Wake Total Sleep Period | Wake within TSP | min |
| SE | Sleep Efficiency | Percentage of time asleep (TST / time in bed × 100) | % |
| **Latencies** | | | |
| SL | Sleep Latency | Time from “lights off” to the first epoch of any sleep stage | min |
| LCONT | Latency to persistent sleep | Time from “lights off” to the first 20 consecutive epochs of any sleep stage | min |
| SLRaK | SL (R&K) | Sleep latency according to Rechtschaffen and Kales (“lights off” to the first three epoch N1 or any other stage) | min |
| RL | REM Latency | Time from sleep onset to the first epoch of stage R | min |
| N3L | N3 Latency | Time from sleep onset to the first epoch of stage N3 | min |
| LN2 | Latency to N2 | Time from “lights off” to the first epoch of stage N2 | min |
| LN3 | Latency to N3 | Time from “lights off” to the first epoch of stage N3 | min |
| LR | Latency to R | Time from “lights off” to the first epoch of stage R | min |
| **Sleep stages** | | | |
| N1 | Time in Stage N1 | Time spent in sleep stage N1 | min |
| N1P | Stage N1 Percentage | Percentage of TST spent in stage N1 | % |
| N2 | Time in Stage N2 | Time spent in sleep stage N2 | min |
| N2P | Stage N2 Percentage | Percentage of TST spent in stage N2 | % |
| N3 | Time in Stage N3 | Time spent in sleep stage N3 | min |
| N3P | Stage N3 Percentage | Percentage of TST spent in stage N3 | % |
| R | Time in Stage R | Time spent in R sleep | min |
| RP | R Percentage | Percentage of TST spent in stage R | % |
| ***Awakenings and stage shifts*** | | | |
| FW | Frequency of Awakenings | Number of awakenings in TST | # |
| FWTST | Frequency of Awakenings / hr TST | Number of awakenings per hour TST | # |
| FS | Frequency of Stage Shifts | Number of stage shifts in TST | # |
| FSTST | Frequency of Stage Shifts / hr TST | Number of stage shifts per hour TST | # |

# **Table S3.** Correlations (Pearson r) of subjective assessments of sleep quality with the plasma concentration of six AD-biomarkers in the A) evening, in B) morning and with C) overnight changes. Statistically significant correlations are shown in bold.

| **A) evening** |  | **p-tau217** | **p-tau181** | **t-tau** | **Aß42** | **Aß40** | **Aß42/40** |
| --- | --- | --- | --- | --- | --- | --- | --- |
| Restfulness | r | 0.12551 | 0.14913 | 0.02430 | 0.00964 | -0.06983 | 0.07459 |
|  | p | 0.4403 | 0.3584 | 0.8817 | 0.9529 | 0.6686 | 0.6473 |
|  | n | 40 | 40 | 40 | 40 | 40 | 40 |
| Sleep latency | r | 0.11221 | 0.00411 | -0.05140 | **0.36517** | 0.30004 | 0.10495 |
|  | p | 0.5024 | 0.9805 | 0.7593 | **0.0242** | 0.0672 | 0.5306 |
|  | n | 38 | 38 | 38 | 38 | 38 | 38 |
| Number of awakenings | r | 0.07665 | 0.04862 | 0.05134 | -0.14464 | -0.18159 | 0.00260 |
|  | p | 0.6568 | 0.7783 | 0.7662 | 0.400 | 0.2892 | 0.9880 |
|  | n | 36 | 36 | 36 | 36 | 36 | 36 |
| Wake duration | r | 0.06821 | 0.05782 | 0.00986 | -0.07768 | -0.26683 | 0.25973 |
|  | p | 0.6970 | 0.7415 | 0.9552 | 0.6574 | 0.1213 | 0.1319 |
|  | n | 35 | 35 | 35 | 35 | 35 | 35 |
| Total sleep time | r | -0.18076 | -0.08664 | 0.04322 | -0.11025 | -0.05504 | -0.10085 |
|  | p | 0.2708 | 0.6000 | 0.7939 | 0.5040 | 0.7393 | 0.5413 |
|  | n | 39 | 39 | 39 | 39 | 39 | 39 |
| **B) morning** |  | **p-tau217** | **p-tau181** | **t-tau** | **Aß42** | **Aß40** | **Aß42/40** |
| Restfulness | r | 0.04453 | 0.06839 | 0.10476 | -0.06479 | -0.01442 | -0.02071 |
|  | p | 0.7878 | 0.6750 | 0.5314 | 0.6992 | 0.9315 | 0.9018 |
|  | n | 39 | 40 | 38 | 38 | 38 | 38 |
| Sleep latency | r | 0.14321 | 0.17031 | -0.01303 | **0.39498** | **0.38112** | -0.00086 |
|  | p | 0.3978 | 0.3066 | 0.9399 | **0.0171** | **0.0218** | 0.9960 |
|  | n | 37 | 38 | 36 | 36 | 36 | 36 |
| Number of awake-nings | r | -0.06835 | 0.01355 | 0.23694 | -0.13216 | -0.19534 | 0.22797 |
|  | p | 0.6964 | 0.9375 | 0.1773 | 0.4562 | 0.2682 | 0.1947 |
|  | n | 35 | 36 | 34 | 34 | 34 | 34 |
| Wake duration | r | -0.11190 | 0.14647 | **0.44047** | -0.32701 | **-0.43214** | **0.41086** |
|  | p | 0.5353 | 0.4085 | **0.0116** | 0.0677 | **0.0135** | **0.0195** |
|  | n | 33 | 34 | 32 | 32 | 32 | 32 |
| Total sleep time | r | -0.13201 | -0.21627 | -0.12679 | -0.08625 | -0.07893 | -0.12105 |
|  | p | 0.4295 | 0.1861 | 0.4546 | 0.6117 | 0.6424 | 0.4754 |
|  | n | 38 | 39 | 37 | 37 | 37 | 37 |
| **C) overnight** |  | **p-tau217** | **p-tau181** | **t-tau** | **Aß42** | **Aß40** | **Aß42/40** |
| Restfulness | r | -0.10348 | -0.07277 | 0.11499 | -0.10232 | 0.02300 | -0.05471 |
|  | p | 0.5307 | 0.6554 | 0.4918 | 0.5410 | 0.8910 | 0.7442 |
|  | n | 39 | 40 | 38 | 38 | 38 | 38 |
| Sleep latency | r | 0.06909 | 0.13744 | 0.01965 | 0.06180 | 0.13511 | -0.10401 |
|  | p | 0.6845 | 0.4106 | 0.9094 | 0.7203 | 0.4321 | 0.5461 |
|  | n | 37 | 38 | 36 | 36 | 36 | 36 |
| Number of awake-nings | r | -0.31171 | -0.03266 | 0.24050 | 0.03915 | -0.05462 | 0.33582 |
|  | p | 0.0683 | 0.8500 | 0.1707 | 0.8260 | 0.7590 | 0.0522 |
|  | n | 35 | 36 | 34 | 34 | 34 | 34 |
| Wake duration | r | -0.23963 | 0.11216 | **0.47278** | **-0.42409** | -0.27494 | 0.26141 |
|  | p | 0.1792 | 0.5277 | **0.0063** | **0.0156** | 0.1278 | 0.1484 |
|  | n | 33 | 34 | 32 | 32 | 32 | 32 |
| Total sleep time | r | 0.00674 | -0.10232 | -0.16775 | 0.02483 | -0.01611 | -0.07170 |
|  | p | 0.9680 | 0.5354 | 0.3210 | 0.8840 | 0.9246 | 0.6732 |
|  | n | 38 | 39 | 37 | 37 | 37 | 37 |

# **Table S4.** Correlations (Pearson r) of macrostructure of sleep with the plasma concentration of six AD-biomarkers in the evening. Definitions of the abbreviations can be found in Table S2. Statistically significant correlations are shown in bold.

| ***Sleep continuity*** | | | | | | | |
| --- | --- | --- | --- | --- | --- | --- | --- |
| **evening** |  | **p-tau217** | **p-tau181** | **t-tau** | **Aß42** | **Aß40** | **Aß42/40** |
| TST | r | -0.14775 | -0.14580 | 0.01710 | 0.12984 | 0.14566 | -0.05244 |
|  | p | 0.3629 | 0.3694 | 0.9166 | 0.4246 | 0.3698 | 0.7479 |
|  | n | 40 | 40 | 40 | 40 | 40 | 40 |
| TSP | r | -0.10151 | -0.05245 | 0.03376 | 0.07251 | 0.03529 | 0.03897 |
|  | p | 0.5331 | 0.7479 | 0.8362 | 0.6566 | 0.8288 | 0.8113 |
|  | n | 40 | 40 | 40 | 40 | 40 | 40 |
| WASO | r | 0.12897 | 0.18504 | 0.10077 | -0.08706 | -0.11551 | 0.06588 |
|  | p | 0.4277 | 0.2530 | 0.5361 | 0.5932 | 0.4779 | 0.6863 |
|  | n | 40 | 40 | 40 | 40 | 40 | 40 |
| WTSP | r | 0.09585 | 0.12892 | 0.00474 | -0.09625 | -0.14111 | 0.08799 |
|  | p | 0.5563 | 0.4279 | 0.9768 | 0.5547 | 0.3851 | 0.5893 |
|  | n | 40 | 40 | 40 | 40 | 40 | 40 |
| SE | r | -0.14780 | -0.14582 | 0.01710 | 0.12976 | 0.14560 | -0.05246 |
|  | p | 0.3628 | 0.3693 | 0.9166 | 0.4249 | 0.3700 | 0.7478 |
|  | n | 40 | 40 | 40 | 40 | 40 | 40 |
| ***Latencies*** | | | | | | | |
| **evening** |  | **p-tau217** | **p-tau181** | **t-tau** | **Aß42** | **Aß40** | **Aß42/40** |
| SL | r | 0.06421 | -0.02764 | -0.18965 | -0.09781 | -0.08164 | -0.00888 |
|  | p | 0.6939 | 0.8655 | 0.2412 | 0.5482 | 0.6165 | 0.9566 |
|  | n | 40 | 40 | 40 | 40 | 40 | 40 |
| LCONT | r | 0.12016 | 0.01866 | -0.13295 | -0.01464 | 0.08192 | -0.11971 |
|  | p | 0.4602 | 0.9090 | 0.4134 | 0.9285 | 0.6153 | 0.4619 |
|  | n | 40 | 40 | 40 | 40 | 40 | 40 |
| SLRaK | r | 0.06962 | -0.02803 | -0.19040 | -0.09661 | -0.07735 | -0.01284 |
|  | p | 0.6695 | 0.8637 | 0.2393 | 0.5532 | 0.6352 | 0.9373 |
|  | n | 40 | 40 | 40 | 40 | 40 | 40 |
| RL | r | -0.19959 | -0.15630 | -0.13750 | 0.03165 | -0.09608 | 0.10896 |
|  | p | 0.2169 | 0.3355 | 0.3975 | 0.8463 | 0.5553 | 0.5033 |
|  | n | 40 | 40 | 40 | 40 | 40 | 40 |
| N3L | r | -0.08280 | 0.04440 | 0.05635 | -0.07155 | -0.02840 | -0.06199 |
|  | p | 0.6163 | 0.7884 | 0.7333 | 0.6651 | 0.8637 | 0.7077 |
|  | n | 39 | 39 | 39 | 39 | 39 | 39 |
| LN2 | r | 0.05532 | -0.02485 | -0.18182 | -0.10339 | -0.09342 | -0.00051 |
|  | p | 0.7346 | 0.8790 | 0.2615 | 0.5255 | 0.5664 | 0.9975 |
|  | n | 40 | 40 | 40 | 40 | 40 | 40 |
| LN3 | r | -0.02361 | 0.02298 | -0.07375 | -0.12563 | -0.09288 | -0.03792 |
|  | p | 0.8866 | 0.8895 | 0.6555 | 0.4460 | 0.5739 | 0.8187 |
|  | n | 39 | 39 | 39 | 39 | 39 | 39 |
| LR | r | -0.16887 | -0.16345 | -0.21002 | -0.00832 | -0.12638 | 0.10268 |
|  | p | 0.2976 | 0.3136 | 0.1933 | 0.9593 | 0.4371 | 0.5284 |
|  | n | 40 | 40 | 40 | 40 | 40 | 40 |

**Table S4** (continued). Correlations (Pearson r) of macrostructure of sleep with the plasma concentration of six AD-biomarkers in the evening. Definitions of the abbreviations can be found in Table S2. Statistically significant correlations are shown in bold.

| ***Sleep stages*** | | | | | | | |
| --- | --- | --- | --- | --- | --- | --- | --- |
| **evening** |  | **p-tau217** | **p-tau181** | **t-tau** | **Aß42** | **Aß40** | **Aß42/40** |
| N1 | r | 0.23053 | 0.23732 | 0.09317 | -0.00390 | 0.00196 | -0.00480 |
|  | p | 0.1524 | 0.1404 | 0.5674 | 0.9809 | 0.9904 | 0.9765 |
|  | n | 40 | 40 | 40 | 40 | 40 | 40 |
| N1P | r | 0.23598 | 0.26536 | 0.11336 | -0.04790 | -0.04597 | 0.00910 |
|  | p | 0.1427 | 0.0980 | 0.4861 | 0.7691 | 0.7782 | 0.9555 |
|  | n | 40 | 40 | 40 | 40 | 40 | 40 |
| N2 | r | -0.13057 | 0.16364 | 0.28492 | -0.07111 | -0.10868 | 0.01324 |
|  | p | 0.4220 | 0.3130 | 0.0748 | 0.6628 | 0.5044 | 0.9354 |
|  | n | 40 | 40 | 40 | 40 | 40 | 40 |
| N2P | r | -0.06237 | 0.25076 | 0.30962 | -0.16692 | -0.22130 | 0.04859 |
|  | p | 0.7022 | 0.1186 | 0.0519 | 0.3033 | 0.1700 | 0.7659 |
|  | n | 40 | 40 | 40 | 40 | 40 | 40 |
| N3 | r | -0.11415 | **-0.35021** | **-0.34245** | 0.14484 | 0.19654 | -0.06407 |
|  | p | 0.4831 | **0.0267** | **0.0305** | 0.3725 | 0.2242 | 0.6945 |
|  | n | 40 | 40 | 40 | 40 | 40 | 40 |
| N3P | r | -0.06211 | **-0.32598** | **-0.38075** | 0.14147 | 0.18777 | -0.04948 |
|  | p | 0.7034 | **0.0401** | **0.0154** | 0.3839 | 0.2460 | 0.7617 |
|  | n | 40 | 40 | 40 | 40 | 40 | 40 |
| R | r | -0.09868 | -0.14922 | 0.06372 | 0.09596 | 0.09139 | -0.00504 |
|  | p | 0.5447 | 0.3581 | 0.6961 | 0.5559 | 0.5749 | 0.9754 |
|  | n | 40 | 40 | 40 | 40 | 40 | 40 |
| RP | r | -0.06544 | -0.12072 | 0.06791 | 0.06683 | 0.06721 | 0.00177 |
|  | p | 0.6883 | 0.4581 | 0.6771 | 0.6820 | 0.6803 | 0.9913 |
|  | n | 40 | 40 | 40 | 40 | 40 | 40 |
| ***Awakenings and stage shifts*** | | | | | | | |
| **evening** |  | **p-tau217** | **p-tau181** | **t-tau** | **Aß42** | **Aß40** | **Aß42/40** |
| FW | r | 0.11455 | 0.13666 | 0.03771 | -0.04748 | 0.01975 | -0.14593 |
|  | p | 0.4815 | 0.4004 | 0.8173 | 0.7711 | 0.9037 | 0.3689 |
|  | n | 40 | 40 | 40 | 40 | 40 | 40 |
| FWTST | r | 0.13625 | 0.15504 | 0.04835 | -0.06408 | -0.00537 | -0.12717 |
|  | p | 0.4019 | 0.3395 | 0.7670 | 0.6945 | 0.9738 | 0.4342 |
|  | n | 40 | 40 | 40 | 40 | 40 | 40 |
| FS | r | 0.10747 | 0.12001 | -0.09244 | -0.15300 | 0.08008 | **-0.32259** |
|  | p | 0.5092 | 0.4607 | 0.5705 | 0.3459 | 0.6233 | **0.0423** |
|  | n | 40 | 40 | 40 | 40 | 40 | 40 |
| FSTST | r | 0.15809 | 0.16508 | -0.08738 | -0.18731 | 0.05004 | **-0.31799** |
|  | p | 0.3299 | 0.3087 | 0.5918 | 0.2471 | 0.7591 | **0.0455** |
|  | n | 40 | 40 | 40 | 40 | 40 | 40 |

# **Table S5.** Correlations (Pearson r) of macrostructure of sleep with the plasma concentration of six AD-biomarkers in the morning. Definitions of the abbreviations can be found in Table S2. Statistically significant correlations are shown in bold.

| ***Sleep continuity*** | | | | | | | |
| --- | --- | --- | --- | --- | --- | --- | --- |
| **morning** |  | **p-tau217** | **p-tau181** | **t-tau** | **Aß42** | **Aß40** | **Aß42/40** |
| TST | r | -0.03797 | -0.08790 | -0.22632 | 0.15073 | 0.13157 | -0.11864 |
|  | p | 0.8185 | 0.5896 | 0.1718 | 0.3664 | 0.4310 | 0.4780 |
|  | n | 39 | 40 | 38 | 38 | 38 | 38 |
| TSP | r | -0.27806 | -0.24650 | 0.16499 | -0.09490 | -0.14302 | 0.12857 |
|  | p | 0.0865 | 0.1252 | 0.3222 | 0.5709 | 0.3917 | 0.4417 |
|  | n | 39 | 40 | 38 | 38 | 38 | 38 |
| WASO | r | -0.11972 | -0.03606 | **0.37615** | -0.20079 | -0.21204 | 0.22367 |
|  | p | 0.4679 | 0.8252 | **0.0199** | 0.2268 | 0.2012 | 0.1771 |
|  | n | 39 | 40 | 38 | 38 | 38 | 38 |
| WTSP | r | -0.15678 | -0.07689 | **0.37824** | -0.24108 | -0.25390 | 0.22867 |
|  | p | 0.3405 | 0.6372 | **0.0192** | 0.1448 | 0.1240 | 0.1673 |
|  | n | 39 | 40 | 38 | 38 | 38 | 38 |
| SE | r | -0.03804 | -0.08795 | -0.22641 | 0.15069 | 0.13157 | -0.11873 |
|  | p | 0.8181 | 0.5894 | 0.1717 | 0.3665 | 0.4311 | 0.4777 |
|  | n | 39 | 40 | 38 | 38 | 38 | 38 |
| ***Latencies*** | | | | | | | |
| **morning** |  | **p-tau217** | **p-tau181** | **t-tau** | **Aß42** | **Aß40** | **Aß42/40** |
| SL | r | 0.25714 | 0.21585 | -0.18242 | 0.04335 | 0.09563 | -0.13723 |
|  | p | 0.1140 | 0.1810 | 0.2730 | 0.7961 | 0.5679 | 0.4113 |
|  | n | 39 | 40 | 38 | 38 | 38 | 38 |
| LCONT | r | 0.30812 | 0.18063 | -0.18475 | 0.14083 | 0.19615 | -0.16780 |
|  | p | 0.0563 | 0.2647 | 0.2668 | 0.3990 | 0.2379 | 0.3139 |
|  | n | 39 | 40 | 38 | 38 | 38 | 38 |
| SLRaK | r | 0.26109 | 0.21662 | -0.18407 | 0.04747 | 0.10308 | -0.14148 |
|  | p | 0.1084 | 0.1794 | 0.2686 | 0.7772 | 0.5380 | 0.3968 |
|  | n | 39 | 40 | 38 | 38 | 38 | 38 |
| RL | r | -0.16341 | -0.13746 | 0.03047 | 0.12701 | 0.08416 | 0.05585 |
|  | p | 0.3202 | 0.3977 | 0.8559 | 0.4473 | 0.6154 | 0.7391 |
|  | n | 39 | 40 | 38 | 38 | 38 | 38 |
| N3L | r | 0.02946 | 0.01441 | -0.01428 | -0.00418 | -0.06348 | 0.10100 |
|  | p | 0.8606 | 0.9306 | 0.9331 | 0.9804 | 0.7089 | 0.5520 |
|  | n | 38 | 39 | 37 | 37 | 37 | 37 |
| LN2 | r | 0.25697 | 0.23069 | -0.16235 | 0.02968 | 0.07669 | -0.11745 |
|  | p | 0.1143 | 0.1521 | 0.3301 | 0.8596 | 0.6472 | 0.4825 |
|  | n | 39 | 40 | 38 | 38 | 38 | 38 |
| LN3 | r | 0.19522 | 0.20248 | -0.13751 | 0.00596 | -0.01040 | 0.01704 |
|  | p | 0.2402 | 0.2164 | 0.4170 | 0.9721 | 0.9513 | 0.9202 |
|  | n | 38 | 39 | 37 | 37 | 37 | 37 |
| LR | r | -0.05216 | -0.04755 | -0.04354 | 0.14203 | 0.12105 | -0.00046 |
|  | p | 0.7525 | 0.7708 | 0.7952 | 0.3950 | 0.4691 | 0.9978 |
|  | n | 39 | 40 | 38 | 38 | 38 | 38 |

**Table S5** (Continued). Correlations (Pearson r) of macrostructure of sleep with the plasma concentration of six AD-biomarkers in the morning. Definitions of the abbreviations can be found in Table S2. Statistically significant correlations are shown in bold.

| ***Sleep stages*** | | | | | | | |
| --- | --- | --- | --- | --- | --- | --- | --- |
| **morning** |  | **p-tau217** | **p-tau181** | **t-tau** | **Aß42** | **Aß40** | **Aß42/40** |
| N1 | r | 0.14201 | 0.10029 | 0.12677 | 0.10404 | 0.11173 | 0.05325 |
|  | p | 0.3885 | 0.5381 | 0.4482 | 0.5342 | 0.5042 | 0.7509 |
|  | n | 39 | 40 | 38 | 38 | 38 | 38 |
| N1P | r | 0.11958 | 0.11981 | 0.21529 | 0.02957 | 0.04363 | 0.09768 |
|  | p | 0.4684 | 0.4615 | 0.1943 | 0.8601 | 0.7948 | 0.5596 |
|  | n | 39 | 40 | 38 | 38 | 38 | 38 |
| N2 | r | -0.14982 | 0.05264 | 0.12476 | -0.10558 | -0.19159 | 0.13848 |
|  | p | 0.3626 | 0.7470 | 0.4555 | 0.5281 | 0.2492 | 0.4070 |
|  | n | 39 | 40 | 38 | 38 | 38 | 38 |
| N2P | r | -0.14632 | 0.10915 | 0.31590 | -0.23405 | **-0.32707** | 0.25756 |
|  | p | 0.3741 | 0.5026 | 0.0533 | 0.1573 | **0.0450** | 0.1185 |
|  | n | 39 | 40 | 38 | 38 | 38 | 38 |
| N3 | r | 0.00884 | -0.18209 | **-0.35426** | 0.17629 | 0.24127 | -0.24913 |
|  | p | 0.9574 | 0.2608 | **0.0291** | 0.2897 | 0.1445 | 0.1315 |
|  | n | 39 | 40 | 38 | 38 | 38 | 38 |
| N3P | r | 0.03750 | -0.17299 | **-0.36753** | 0.18058 | 0.25857 | -0.26500 |
|  | p | 0.8207 | 0.2858 | **0.0232** | 0.2780 | 0.1170 | 0.1079 |
|  | n | 39 | 40 | 38 | 38 | 38 | 38 |
| R | r | 0.00211 | -0.03450 | -0.12196 | 0.03447 | 0.01511 | -0.05600 |
|  | p | 0.9898 | 0.8326 | 0.4657 | 0.8372 | 0.9282 | 0.7384 |
|  | n | 39 | 40 | 38 | 38 | 38 | 38 |
| RP | r | 0.02692 | 0.00080 | -0.07190 | -0.00334 | -0.01952 | -0.02937 |
|  | p | 0.8708 | 0.9961 | 0.6680 | 0.9841 | 0.9074 | 0.8611 |
|  | n | 39 | 40 | 38 | 38 | 38 | 38 |
| ***Awakenings and stage shifts*** | | | | | | | |
| **morning** |  | **p-tau217** | **p-tau181** | **t-tau** | **Aß42** | **Aß40** | **Aß42/40** |
| FW | r | -0.01524 | -0.19117 | -0.16274 | 0.04949 | 0.21474 | -0.31008 |
|  | p | 0.9266 | 0.2373 | 0.3290 | 0.7680 | 0.1954 | 0.0581 |
|  | n | 39 | 40 | 38 | 38 | 38 | 38 |
| FWTST | r | -0.01361 | -0.18545 | -0.11438 | 0.01554 | 0.17785 | -0.27777 |
|  | p | 0.9345 | 0.2519 | 0.4941 | 0.9262 | 0.2854 | 0.0913 |
|  | n | 39 | 40 | 38 | 38 | 38 | 38 |
| FS | r | 0.06996 | -0.23723 | **-0.42045** | 0.07274 | 0.23097 | **-0.42868** |
|  | p | 0.6721 | 0.1405 | **0.0086** | 0.6643 | 0.1630 | **0.00072** |
|  | n | 39 | 40 | 38 | 38 | 38 | 38 |
| FSTST | r | 0.07689 | -0.24516 | **-0.38969** | 0.03222 | 0.20722 | **-0.42789** |
|  | p | 0.6418 | 0.1273 | **0.0156** | 0.8477 | 0.2119 | **0.0074** |
|  | n | 39 | 40 | 38 | 38 | 38 | 38 |

# **Table S6.** Correlations (Pearson r) of macrostructure of sleep with overnight changes in plasma concentration of six AD-biomarkers. Definitions of the abbreviations can be found in Table S2. Statistically significant correlations are shown in bold.

| ***Sleep continuity*** | | | | | | | |
| --- | --- | --- | --- | --- | --- | --- | --- |
| **Overnight changes** |  | **p-tau217** | **p-tau181** | **t-tau** | **Aß42** | **Aß40** | **Aß42/40** |
| TST | r | 0.12625 | 0.05360 | -0.24992 | 0.03059 | -0.01414 | -0.08757 |
|  | p | 0.4438 | 0.7426 | 0.1302 | 0.8553 | 0.9328 | 0.6011 |
|  | n | 39 | 40 | 38 | 38 | 38 | 38 |
| TSP | r | -0.24066 | -0.15998 | 0.15655 | -0.28648 | -0.23338 | 0.09226 |
|  | p | 0.1400 | 0.3241 | 0.3479 | 0.0812 | 0.1585 | 0.5817 |
|  | n | 39 | 40 | 38 | 38 | 38 | 38 |
| WASO | r | -0.31324 | -0.19118 | **0.33192** | -0.18982 | -0.12934 | 0.19235 |
|  | p | 0.0522 | 0.2373 | **0.0418** | 0.2537 | 0.4390 | 0.2473 |
|  | n | 39 | 40 | 38 | 38 | 38 | 38 |
| WTSP | r | **-0.31760** | -0.17639 | **0.39916** | -0.24191 | -0.15234 | 0.16687 |
|  | p | **0.0488** | 0.2763 | **0.0130** | 0.1434 | 0.3612 | 0.3167 |
|  | n | 39 | 40 | 38 | 38 | 38 | 38 |
| SE | r | 0.12620 | 0.05359 | -0.25002 | 0.03067 | -0.01408 | -0.08764 |
|  | p | 0.4439 | 0.7426 | 0.1301 | 0.8549 | 0.9332 | 0.6008 |
|  | n | 39 | 40 | 38 | 38 | 38 | 38 |
| ***Latencies*** | | | | | | | |
| **Overnight changes** |  | **p-tau217** | **p-tau181** | **t-tau** | **Aß42** | **Aß40** | **Aß42/40** |
| SL | r | 0.26451 | 0.20415 | -0.07031 | 0.24300 | 0.22874 | -0.14412 |
|  | p | 0.1037 | 0.2064 | 0.6749 | 0.1415 | 0.1672 | 0.3880 |
|  | n | 39 | 40 | 38 | 38 | 38 | 38 |
| LCONT | r | 0.27863 | 0.13445 | -0.10921 | 0.26260 | 0.15748 | -0.04965 |
|  | p | 0.0859 | 0.4082 | 0.5139 | 0.1112 | 0.3450 | 0.7672 |
|  | n | 39 | 40 | 38 | 38 | 38 | 38 |
| SLRuK | r | 0.26381 | 0.20512 | -0.07116 | 0.24704 | 0.23337 | -0.14536 |
|  | p | 0.1046 | 0.2042 | 0.6712 | 0.1349 | 0.1585 | 0.3839 |
|  | n | 39 | 40 | 38 | 38 | 38 | 38 |
| RL | r | -0.04114 | 0.02141 | 0.12141 | 0.16288 | 0.24448 | -0.05549 |
|  | p | 0.8036 | 0.8957 | 0.4678 | 0.3285 | 0.1391 | 0.7407 |
|  | n | 39 | 40 | 38 | 38 | 38 | 38 |
| N3L | r | 0.16572 | -0.02803 | -0.09007 | 0.10682 | -0.01620 | 0.17060 |
|  | p | 0.3201 | 0.8655 | 0.5960 | 0.5292 | 0.9242 | 0.3127 |
|  | n | 38 | 39 | 37 | 37 | 37 | 37 |
| LN2 | r | 0.27730 | 0.21409 | -0.05393 | 0.22963 | 0.21776 | -0.12893 |
|  | p | 0.0874 | 0.1847 | 0.7478 | 0.1655 | 0.1891 | 0.4405 |
|  | n | 39 | 40 | 38 | 38 | 38 | 38 |
| LN3 | r | 0.30803 | 0.13963 | -0.04769 | 0.23140 | 0.12783 | 0.06222 |
|  | p | 0.0599 | 0.3966 | 0.7793 | 0.1682 | 0.4509 | 0.7145 |
|  | n | 38 | 39 | 37 | 37 | 37 | 37 |
| LR | r | 0.06941 | 0.10264 | 0.09078 | 0.25758 | **0.33189** | -0.11244 |
|  | p | 0.6746 | 0.5285 | 0.5878 | 0.1185 | **0.0418** | 0.5015 |
|  | n | 39 | 40 | 38 | 38 | 38 | 38 |

**Table S6** (Continued). Correlations (Pearson r) of macrostructure of sleep with overnight changes in plasma concentration of six AD-biomarkers. Definitions of the abbreviations can be found in Table S2. Statistically significant correlations are shown in bold.

| ***Sleep stages*** | | | | | | | |
| --- | --- | --- | --- | --- | --- | --- | --- |
| **Overnight changes** |  | **p-tau217** | **p-tau181** | **t-tau** | **Aß42** | **Aß40** | **Aß42/40** |
| N1 | r | -0.05479 | -0.12294 | 0.08184 | 0.16842 | 0.15552 | 0.06504 |
|  | p | 0.7404 | 0.4498 | 0.6252 | 0.3121 | 0.3512 | 0.6981 |
|  | n | 39 | 40 | 38 | 38 | 38 | 38 |
| N1P | r | -0.09132 | -0.13107 | 0.16183 | 0.12084 | 0.12363 | 0.10862 |
|  | p | 0.5803 | 0.4202 | 0.3317 | 0.4699 | 0.4596 | 0.5163 |
|  | n | 39 | 40 | 38 | 38 | 38 | 38 |
| N2 | r | -0.04321 | -0.09854 | -0.06866 | -0.04655 | -0.12430 | 0.17695 |
|  | p | 0.7939 | 0.5452 | 0.6821 | 0.7814 | 0.4571 | 0.2879 |
|  | n | 39 | 40 | 38 | 38 | 38 | 38 |
| N2P | r | -0.11944 | -0.12724 | 0.11385 | -0.09182 | -0.15564 | 0.28595 |
|  | p | 0.4689 | 0.4340 | 0.4961 | 0.5835 | 0.3508 | 0.0818 |
|  | n | 39 | 40 | 38 | 38 | 38 | 38 |
| N3 | r | 0.13566 | 0.15299 | -0.15038 | 0.05252 | 0.07864 | -0.24547 |
|  | p | 0.4102 | 0.3460 | 0.3675 | 0.7541 | 0.6389 | 0.1374 |
|  | n | 39 | 40 | 38 | 38 | 38 | 38 |
| N3P | r | 0.11120 | 0.13947 | -0.14114 | 0.06621 | 0.11661 | -0.28562 |
|  | p | 0.5003 | 0.3907 | 0.3980 | 0.6929 | 0.4857 | 0.0822 |
|  | n | 39 | 40 | 38 | 38 | 38 | 38 |
| R | r | 0.11023 | 0.10112 | -0.16459 | -0.11266 | -0.10939 | -0.07221 |
|  | p | 0.5041 | 0.5347 | 0.3234 | 0.5007 | 0.5133 | 0.6666 |
|  | n | 39 | 40 | 38 | 38 | 38 | 38 |
| RP | r | 0.09743 | 0.10576 | -0.11316 | -0.12751 | -0.12419 | -0.04925 |
|  | p | 0.5552 | 0.5160 | 0.4988 | 0.4455 | 0.4576 | 0.7691 |
|  | n | 39 | 40 | 38 | 38 | 38 | 38 |
| ***Awakenings and stage shifts*** | | | | | | | |
| **Overnight changes** |  | **p-tau217** | **p-tau181** | **t-tau** | **Aß42** | **Aß40** | **Aß42/40** |
| FW | r | -0.12901 | -0.27846 | -0.20811 | 0.13046 | 0.32321 | -0.30079 |
|  | p | 0.4338 | 0.0819 | 0.2099 | 0.4350 | **0.0478** | 0.0665 |
|  | n | 39 | 40 | 38 | 38 | 38 | 38 |
| FWTST | r | -0.15312 | -0.28969 | -0.16560 | 0.10573 | 0.30590 | -0.28047 |
|  | p | 0.3520 | 0.0698 | 0.3204 | 0.5276 | 0.0618 | 0.0881 |
|  | n | 39 | 40 | 38 | 38 | 38 | 38 |
| FS | r | -0.01479 | -0.30239 | **-0.39091** | **0.36053** | 0.22815 | -0.17773 |
|  | p | 0.9288 | 0.0579 | **0.0152** | **0.0262** | 0.1683 | 0.2857 |
|  | n | 39 | 40 | 38 | 38 | 38 | 38 |
| FSTST | r | -0.06558 | -0.34825 | **-0.36490** | **0.35048** | 0.23765 | -0.18525 |
|  | p | 0.6916 | **0.0277** | **0.0243** | **0.0310** | 0.1508 | 0.2655 |
|  | n | 39 | 40 | 38 | 38 | 38 | 38 |

# **Table S7.** Correlations (Pearson r) of sleep spindle parameters (length = duration, amplitude, frequency, density) in stage N2 at four electrode sites (F3, F4, P3, P4) with plasma concentration of six AD-biomarkers collected in the evening. Statistically significant correlations are shown in bold.

| **evening** |  | **p-tau217** | **p-tau181** | **t-tau** | **Aß42** | **Aß40** | **Aß42/40** |
| --- | --- | --- | --- | --- | --- | --- | --- |
| avL_F3 | r | -0.17083 | -0.09481 | 0.04527 | **-0.53051** | **-0.41208** | -0.15730 |
|  | p | 0.2984 | 0.5659 | 0.7843 | **0.0005** | **0.0091** | 0.3389 |
|  | n | 39 | 39 | 39 | 39 | 39 | 39 |
| avA_F3 | r | -0.08229 | -0.11945 | -0.09394 | -0.26743 | -0.27876 | 0.00523 |
|  | p | 0.6184 | 0.4689 | 0.5695 | 0.0998 | 0.0857 | 0.9748 |
|  | n | 39 | 39 | 39 | 39 | 39 | 39 |
| avF_F3 | r | -0.30082 | **-0.32232** | -0.19753 | -0.16968 | -0.27796 | 0.17160 |
|  | p | 0.0628 | **0.0454** | 0.2281 | 0.3018 | 0.0866 | 0.2962 |
|  | n | 39 | 39 | 39 | 39 | 39 | 39 |
| avD_F3 | r | -0.16883 | -0.08405 | 0.03240 | **-0.50320** | **-0.53557** | 0.04393 |
|  | p | 0.3042 | 0.6109 | 0.8448 | **0.0011** | **0.0004** | 0.7906 |
|  | n | 39 | 39 | 39 | 39 | 39 | 39 |
| avL_F4 | r | -0.28034 | -0.21945 | -0.02849 | **-0.56815** | **-0.42941** | -0.15961 |
|  | p | 0.0798 | 0.1737 | 0.8615 | **0.0001** | **0.0057** | 0.3252 |
|  | n | 40 | 40 | 40 | 40 | 40 | 40 |
| avA_F4 | r | -0.15112 | -0.17316 | -0.10506 | **-0.31804** | -0.30108 | -0.03630 |
|  | p | 0.3519 | 0.2853 | 0.5188 | **0.0455** | 0.0590 | 0.8240 |
|  | n | 40 | 40 | 40 | 40 | 40 | 40 |
| avF_F4 | r | -0.28064 | -0.27044 | -0.12989 | -0.12501 | -0.24457 | 0.18207 |
|  | p | 0.0794 | 0.0915 | 0.4244 | 0.4421 | 0.1283 | 0.2608 |
|  | n | 40 | 40 | 40 | 40 | 40 | 40 |
| avD_F4 | r | -0.22304 | -0.18186 | -0.07735 | **-0.54583** | **-0.48577** | -0.07006 |
|  | p | 0.1665 | 0.2614 | 0.6352 | **0.0003** | **0.0015** | 0.6675 |
|  | n | 40 | 40 | 40 | 40 | 40 | 40 |
| avL_P3 | r | -0.17654 | -0.26470 | -0.11570 | -0.22914 | **-0.39497** | 0.19543 |
|  | p | 0.2823 | 0.1034 | 0.4831 | 0.1606 | **0.0128** | 0.2331 |
|  | n | 39 | 39 | 39 | 39 | 39 | 39 |
| avA_P3 | r | -0.03273 | -0.02552 | -0.08391 | -0.08168 | -0.16310 | 0.08859 |
|  | p | 0.8432 | 0.8774 | 0.6116 | 0.6211 | 0.3212 | 0.5917 |
|  | n | 39 | 39 | 39 | 39 | 39 | 39 |
| avF_P3 | r | 0.01543 | -0.12899 | -0.15093 | 0.27791 | 0.06200 | 0.28126 |
|  | p | 0.9257 | 0.4338 | 0.3591 | 0.0867 | 0.7077 | 0.0828 |
|  | n | 39 | 39 | 39 | 39 | 39 | 39 |
| avD_P3 | r | -0.16418 | -0.23097 | -0.10428 | -0.16929 | **-0.32998** | 0.20102 |
|  | p | 0.3114 | 0.1516 | 0.5219 | 0.2964 | **0.0376** | 0.2136 |
|  | n | 40 | 40 | 40 | 40 | 40 | 40 |
| avL_P4 | r | -0.17970 | -0.20779 | -0.03778 | -0.23380 | **-0.40255** | 0.17135 |
|  | p | 0.2672 | 0.1982 | 0.8169 | 0.1465 | **0.0100** | 0.2904 |
|  | n | 40 | 40 | 40 | 40 | 40 | 40 |
| avA_P4 | r | 0.01905 | 0.01147 | -0.04032 | -0.08052 | -0.12197 | 0.01317 |
|  | p | 0.9071 | 0.9440 | 0.8049 | 0.6214 | 0.4534 | 0.9357 |
|  | n | 40 | 40 | 40 | 40 | 40 | 40 |
| avF_P4 | r | 0.05007 | -0.06863 | -0.09513 | **0.31213** | 0.05639 | **0.31393** |
|  | p | 0.7590 | 0.6739 | 0.5593 | **0.0499** | 0.7296 | **0.0485** |
|  | n | 40 | 40 | 40 | 40 | 40 | 40 |
| avD_P4 | r | -0.12439 | -0.15695 | 0.00239 | -0.16056 | **-0.32285** | 0.19158 |
|  | p | 0.4444 | 0.3335 | 0.9883 | 0.3223 | **0.0422** | 0.2363 |
|  | n | 40 | 40 | 40 | 40 | 40 | 40 |

avL = averaged spindle length; avA = averaged spindle amplitude; avF = averaged spindle frequency; avD = averaged spindle density

# **Table S8.** Correlations (Pearson r) of sleep spindle parameters (length = duration, amplitude, frequency, density) in stage N3 at four electrode sites (F3, F4, P3, P4) with plasma concentration of six AD-biomarkers collected in the evening. Statistically significant correlations are shown in bold.

| **evening** |  | **p-tau217** | **p-tau181** | **t-tau** | **Aß42** | **Aß40** | **Aß42/40** |
| --- | --- | --- | --- | --- | --- | --- | --- |
| avL_F3 | r | -0.23572 | -0.18674 | -0.13139 | **-0.53204** | **-0.41043** | -0.17518 |
|  | p | 0.1542 | 0.2616 | 0.4317 | **0.0006** | **0.0105** | 0.2928 |
|  | n | 38 | 38 | 38 | 38 | 38 | 38 |
| avA_F3 | r | -0.03056 | -0.06336 | -0.02478 | -0.23459 | -0.24480 | -0.00910 |
|  | p | 0.8555 | 0.7055 | 0.8826 | 0.1563 | 0.1385 | 0.9568 |
|  | n | 38 | 38 | 38 | 38 | 38 | 38 |
| avF_F3 | r | -0.02252 | -0.02256 | -0.05264 | 0.17917 | 0.09331 | 0.11906 |
|  | p | 0.8932 | 0.8931 | 0.7536 | 0.2818 | 0.5774 | 0.4765 |
|  | n | 38 | 38 | 38 | 38 | 38 | 38 |
| avD_F3 | r | -0.19821 | -0.19288 | -0.17681 | **-0.51967** | **-0.39378** | -0.15711 |
|  | p | 0.2264 | 0.2394 | 0.2816 | **0.0007** | **0.0131** | 0.3395 |
|  | n | 39 | 39 | 39 | 39 | 39 | 39 |
| avL_F4 | r | -0.23110 | -0.18254 | -0.08722 | **-0.46159** | **-0.39731** | -0.06594 |
|  | p | 0.1569 | 0.2660 | 0.5975 | **0.0031** | **0.0123** | 0.6900 |
|  | n | 39 | 39 | 39 | 39 | 39 | 39 |
| avA_F4 | r | -0.07813 | -0.08616 | -0.04118 | -0.29927 | -0.28783 | -0.03504 |
|  | p | 0.6364 | 0.6020 | 0.8034 | 0.0642 | 0.0756 | 0.8323 |
|  | n | 39 | 39 | 39 | 39 | 39 | 39 |
| avF_F4 | r | -0.13171 | -0.12161 | -0.00538 | 0.08884 | -0.01425 | 0.10419 |
|  | p | 0.4241 | 0.4608 | 0.9741 | 0.5907 | 0.9314 | 0.5279 |
|  | n | 39 | 39 | 39 | 39 | 39 | 39 |
| avD_F4 | r | -0.20668 | -0.15412 | -0.09476 | **-0.53581** | **-0.42846** | -0.09558 |
|  | p | 0.2068 | 0.3489 | 0.5661 | **0.0004** | **0.0065** | 0.5627 |
|  | n | 39 | 39 | 39 | 39 | 39 | 39 |
| avL_P3 | r | -0.04671 | -0.15924 | -0.12156 | -0.21506 | -0.28913 | 0.07526 |
|  | p | 0.7806 | 0.3396 | 0.4672 | 0.1948 | 0.0783 | 0.6534 |
|  | n | 38 | 38 | 38 | 38 | 38 | 38 |
| avA_P3 | r | -0.03390 | -0.01369 | -0.00852 | -0.12151 | -0.17196 | 0.06999 |
|  | p | 0.8399 | 0.9350 | 0.9595 | 0.4674 | 0.3019 | 0.6763 |
|  | n | 38 | 38 | 38 | 38 | 38 | 38 |
| avF_P3 | r | 0.00936 | -0.13759 | -0.16209 | 0.28307 | 0.09370 | 0.25093 |
|  | p | 0.9555 | 0.4101 | 0.3309 | 0.0851 | 0.5758 | 0.1286 |
|  | n | 38 | 38 | 38 | 38 | 38 | 38 |
| avD_P3 | r | -0.12235 | -0.21416 | -0.04624 | -0.07774 | -0.17179 | 0.11277 |
|  | p | 0.4581 | 0.1905 | 0.7798 | 0.6381 | 0.2957 | 0.4943 |
|  | n | 39 | 39 | 39 | 39 | 39 | 39 |
| avL_P4 | r | -0.11073 | -0.24947 | -0.12175 | -0.27038 | -0.21516 | -0.08452 |
|  | p | 0.5022 | 0.1256 | 0.4603 | 0.0960 | 0.1884 | 0.6089 |
|  | n | 39 | 39 | 39 | 39 | 39 | 39 |
| avA_P4 | r | 0.00836 | 0.05029 | 0.04391 | -0.06297 | -0.12282 | 0.04280 |
|  | p | 0.9597 | 0.7611 | 0.7907 | 0.7033 | 0.4563 | 0.7959 |
|  | n | 39 | 39 | 39 | 39 | 39 | 39 |
| avF_P4 | r | 0.15679 | 0.05188 | -0.21595 | 0.28006 | 0.19959 | 0.08092 |
|  | p | 0.3405 | 0.7538 | 0.1867 | 0.0842 | 0.2232 | 0.6243 |
|  | n | 39 | 39 | 39 | 39 | 39 | 39 |
| avD_P4 | r | -0.12166 | -0.15930 | 0.09481 | -0.08290 | -0.19042 | 0.11708 |
|  | p | 0.4606 | 0.3327 | 0.5659 | 0.6158 | 0.2456 | 0.4778 |
|  | n | 39 | 39 | 39 | 39 | 39 | 39 |

avL = averaged spindle length; avA = averaged spindle amplitude; avF = averaged spindle frequency; avD = averaged spindle density

# **Table S9.** Correlations (Pearson r) of sleep spindle parameters (length = duration, amplitude, frequency, density) in stage NREM at four electrode sites (F3, F4, P3, P4) with plasma concentration of six AD-biomarkers collected in the evening. Statistically significant correlations are shown in bold.

| **evening** |  | **p-tau217** | **p-tau181** | **t-tau** | **Aß42** | **Aß40** | **Aß42/40** |
| --- | --- | --- | --- | --- | --- | --- | --- |
| avL_F3 | r | -0.24141 | -0.14604 | -0.00565 | **-0.57314** | **-0.44774** | -0.16889 |
|  | p | 0.1387 | 0.3750 | 0.9728 | **0.0001** | **0.0043** | 0.3040 |
|  | n | 39 | 39 | 39 | 39 | 39 | 39 |
| avA_F3 | r | -0.07216 | -0.11836 | -0.09607 | -0.26299 | -0.25744 | -0.02835 |
|  | p | 0.6625 | 0.4730 | 0.5607 | 0.1058 | 0.1136 | 0.8640 |
|  | n | 39 | 39 | 39 | 39 | 39 | 39 |
| avF_F3 | r | -0.17874 | -0.19764 | -0.12808 | -0.01213 | -0.10410 | 0.14046 |
|  | p | 0.2763 | 0.2278 | 0.4371 | 0.9416 | 0.5282 | 0.3937 |
|  | n | 39 | 39 | 39 | 39 | 39 | 39 |
| avD_F3 | r | -0.19250 | -0.18927 | -0.11763 | **-0.54801** | **-0.43964** | -0.15432 |
|  | p | 0.2340 | 0.2421 | 0.4698 | **0.0003** | **0.0045** | 0.3417 |
|  | n | 40 | 40 | 40 | 40 | 40 | 40 |
| avL_F4 | r | -0.29159 | -0.21731 | -0.03333 | **-0.57529** | **-0.46358** | -0.12322 |
|  | p | 0.0679 | 0.1780 | 0.8382 | **0.0001** | **0.0026** | 0.4488 |
|  | n | 40 | 40 | 40 | 40 | 40 | 40 |
| avA_F4 | r | -0.13377 | -0.16164 | -0.10995 | **-0.31304** | -0.28177 | -0.05564 |
|  | p | 0.4105 | 0.3190 | 0.4994 | **0.0492** | 0.0782 | 0.7331 |
|  | n | 40 | 40 | 40 | 40 | 40 | 40 |
| avF_F4 | r | -0.23027 | -0.20444 | -0.06254 | -0.05296 | -0.15503 | 0.13909 |
|  | p | 0.1529 | 0.2057 | 0.7015 | 0.7455 | 0.3395 | 0.3920 |
|  | n | 40 | 40 | 40 | 40 | 40 | 40 |
| avD_F4 | r | -0.23650 | -0.20162 | -0.08180 | **-0.58769** | **-0.48972** | -0.11780 |
|  | p | 0.1418 | 0.2122 | 0.6158 | **<0.0001** | **0.0013** | 0.4691 |
|  | n | 40 | 40 | 40 | 40 | 40 | 40 |
| avL_P3 | r | -0.16017 | -0.21425 | -0.08996 | -0.26206 | **-0.40170** | 0.16069 |
|  | p | 0.3300 | 0.1903 | 0.5860 | 0.1070 | **0.0113** | 0.3285 |
|  | n | 39 | 39 | 39 | 39 | 39 | 39 |
| avA_P3 | r | -0.02916 | -0.01855 | -0.08415 | -0.08293 | -0.15919 | 0.08072 |
|  | p | 0.8601 | 0.9107 | 0.6105 | 0.6157 | 0.3330 | 0.6252 |
|  | n | 39 | 39 | 39 | 39 | 39 | 39 |
| avF_P3 | r | 0.00216 | -0.13229 | -0.12603 | 0.30140 | 0.05017 | **0.32884** |
|  | p | 0.9896 | 0.4221 | 0.4446 | 0.0622 | 0.7616 | **0.0410** |
|  | n | 39 | 39 | 39 | 39 | 39 | 39 |
| avD_P3 | r | -0.14347 | -0.21995 | -0.09793 | -0.16072 | -0.30075 | 0.17436 |
|  | p | 0.3771 | 0.1727 | 0.5477 | 0.3218 | 0.0593 | 0.2819 |
|  | n | 40 | 40 | 40 | 40 | 40 | 40 |
| avL_P4 | r | -0.18672 | -0.20121 | -0.03778 | -0.27663 | **-0.44430** | 0.16991 |
|  | p | 0.2486 | 0.2131 | 0.8170 | 0.0840 | **0.0041** | 0.2946 |
|  | n | 40 | 40 | 40 | 40 | 40 | 40 |
| avA_P4 | r | 0.01413 | 0.01188 | -0.03493 | -0.08114 | -0.12232 | 0.01190 |
|  | p | 0.9311 | 0.9420 | 0.8306 | 0.6187 | 0.4521 | 0.9419 |
|  | n | 40 | 40 | 40 | 40 | 40 | 40 |
| avF_P4 | r | 0.08063 | -0.02569 | -0.06390 | **0.32261** | 0.05157 | **0.33376** |
|  | p | 0.6209 | 0.8750 | 0.6953 | **0.0423** | 0.7520 | **0.0353** |
|  | n | 40 | 40 | 40 | 40 | 40 | 40 |
| avD_P4 | r | -0.11292 | -0.14209 | 0.02257 | -0.15579 | -0.30480 | 0.17361 |
|  | p | 0.4878 | 0.3818 | 0.8901 | 0.3371 | 0.0558 | 0.2840 |
|  | n | 40 | 40 | 40 | 40 | 40 | 40 |

avL = averaged spindle length; avA = averaged spindle amplitude; avF = averaged spindle frequency; avD = averaged spindle density

# **Table S10.** Correlations (Pearson r) of sleep spindle parameters (length = duration, amplitude, frequency, density) in stage N2 at four electrode sites (F3, F4, P3, P4) with plasma concentration of six AD-biomarkers collected in the morning. Statistically significant correlations are shown in bold.

| **morning** |  | **p-tau217** | **p-tau181** | **t-tau** | **Aß42** | **Aß40** | **Aß42/40** |
| --- | --- | --- | --- | --- | --- | --- | --- |
| avL_F3 | r | -0.08383 | 0.21789 | 0.23899 | **-0.61228** | **-0.51532** | 0.01457 |
|  | p | 0.6168 | 0.1827 | 0.1543 | **<0.0001** | **0.0011** | 0.9318 |
|  | n | 38 | 39 | 37 | 37 | 37 | 37 |
| avA_F3 | r | -0.03929 | -0.10267 | -0.13948 | -0.28190 | -0.21586 | -0.18912 |
|  | p | 0.8148 | 0.5340 | 0.4103 | 0.0910 | 0.1994 | 0.2623 |
|  | n | 38 | 39 | 37 | 37 | 37 | 37 |
| avF_F3 | r | -0.24204 | -0.16958 | -0.19884 | -0.18502 | -0.23620 | -0.01596 |
|  | p | 0.1432 | 0.3020 | 0.2381 | 0.2729 | 0.1593 | 0.9253 |
|  | n | 38 | 39 | 37 | 37 | 37 | 37 |
| avD_F3 | r | -0.09718 | 0.24596 | 0.27040 | **-0.59400** | **-0.56717** | 0.14056 |
|  | p | 0.5616 | 0.1312 | 0.1055 | **0.0001** | **0.0003** | 0.4067 |
|  | n | 38 | 39 | 37 | 37 | 37 | 37 |
| avL_F4 | r | -0.15390 | 0.10971 | 0.07296 | **-0.58520** | **-0.43620** | -0.15316 |
|  | p | 0.3496 | 0.5004 | 0.6633 | **0.0001** | **0.0062** | 0.3586 |
|  | n | 39 | 40 | 38 | 38 | 38 | 38 |
| avA_F4 | r | -0.07278 | -0.12800 | -0.14490 | -0.31691 | -0.25169 | -0.18092 |
|  | p | 0.6597 | 0.4312 | 0.3854 | 0.0525 | 0.1274 | 0.2770 |
|  | n | 39 | 40 | 38 | 38 | 38 | 38 |
| avF_F4 | r | -0.30924 | -0.18410 | -0.06912 | -0.20476 | -0.24768 | 0.03920 |
|  | p | 0.0554 | 0.2555 | 0.6801 | 0.2175 | 0.1338 | 0.8152 |
|  | n | 39 | 40 | 38 | 38 | 38 | 38 |
| avD_F4 | r | -0.13995 | 0.09946 | 0.09175 | **-0.59140** | **-0.49305** | -0.03509 |
|  | p | 0.3955 | 0.5415 | 0.5838 | **<0.0001** | **0.0017** | 0.8344 |
|  | n | 39 | 40 | 38 | 38 | 38 | 38 |
| avL_P3 | r | -0.06922 | 0.03812 | -0.03626 | -0.21532 | -0.21070 | -0.05742 |
|  | p | 0.6797 | 0.8178 | 0.8313 | 0.2006 | 0.2107 | 0.7357 |
|  | n | 38 | 39 | 37 | 37 | 37 | 37 |
| avA_P3 | r | -0.00133 | -0.07044 | -0.15486 | -0.13783 | -0.12646 | -0.13351 |
|  | p | 0.9937 | 0.6700 | 0.3601 | 0.4159 | 0.4558 | 0.4308 |
|  | n | 38 | 39 | 37 | 37 | 37 | 37 |
| avF_P3 | r | 0.08290 | 0.15826 | 0.05453 | 0.31419 | 0.17794 | 0.23738 |
|  | p | 0.6207 | 0.3359 | 0.7485 | 0.0583 | 0.2920 | 0.1572 |
|  | n | 38 | 39 | 37 | 37 | 37 | 37 |
| avD_P3 | r | -0.04425 | 0.06207 | -0.00771 | -0.19666 | -0.21713 | 0.00893 |
|  | p | 0.7891 | 0.7036 | 0.9634 | 0.2366 | 0.1904 | 0.9576 |
|  | n | 39 | 40 | 38 | 38 | 38 | 38 |
| avL_P4 | r | -0.08883 | 0.04674 | -0.00354 | -0.22726 | -0.24324 | -0.05190 |
|  | p | 0.5907 | 0.7746 | 0.9832 | 0.1700 | 0.1411 | 0.7570 |
|  | n | 39 | 40 | 38 | 38 | 38 | 38 |
| avA_P4 | r | 0.00800 | -0.10509 | -0.12952 | -0.15048 | -0.15250 | -0.12926 |
|  | p | 0.9614 | 0.5187 | 0.4383 | 0.3672 | 0.3607 | 0.4392 |
|  | n | 39 | 40 | 38 | 38 | 38 | 38 |
| avF_P4 | r | 0.11872 | 0.20205 | 0.03702 | **0.36947** | 0.22530 | 0.22363 |
|  | p | 0.4716 | 0.2112 | 0.8254 | **0.0224** | 0.1738 | 0.1771 |
|  | n | 39 | 40 | 38 | 38 | 38 | 38 |
| avD_P4 | r | -0.10143 | 0.01226 | -0.02392 | -0.21228 | -0.25028 | -0.02841 |
|  | p | 0.5389 | 0.9402 | 0.8866 | 0.2007 | 0.1296 | 0.8656 |
|  | n | 39 | 40 | 38 | 38 | 38 | 38 |

avL = averaged spindle length; avA = averaged spindle amplitude; avF = averaged spindle frequency; avD = averaged spindle density

# **Table S11.** Correlations (Pearson r) of sleep spindle parameters (length = duration, amplitude, frequency, density) in stage N3 at four electrode sites (F3, F4, P3, P4) with plasma concentration of six AD-biomarkers collected in the morning. Statistically significant correlations are shown in bold.

| **morning** |  | **p-tau217** | **p-tau181** | **t-tau** | **Aß42** | **Aß40** | **Aß42/40** |
| --- | --- | --- | --- | --- | --- | --- | --- |
| avL_F3 | r | -0.04877 | 0.24312 | 0.09193 | **-0.55141** | **-0.40774** | -0.18536 |
|  | p | 0.7744 | 0.1413 | 0.5939 | **0.0005** | **0.0136** | 0.2791 |
|  | n | 37 | 38 | 36 | 36 | 36 | 36 |
| avA_F3 | r | 0.00339 | 0.02276 | 0.12656 | -0.30415 | -0.25695 | -0.12059 |
|  | p | 0.9841 | 0.8921 | 0.4620 | 0.0713 | 0.1303 | 0.4836 |
|  | n | 37 | 38 | 36 | 36 | 36 | 36 |
| avF_F3 | r | -0.06108 | -0.08772 | 0.12766 | 0.08437 | 0.02833 | 0.06705 |
|  | p | 0.7195 | 0.6005 | 0.4581 | 0.6247 | 0.8697 | 0.6976 |
|  | n | 37 | 38 | 36 | 36 | 36 | 36 |
| avD_F3 | r | 0.01835 | 0.18614 | 0.11684 | **-0.52568** | **-0.39547** | -0.14155 |
|  | p | 0.9129 | 0.2566 | 0.4910 | **0.0008** | **0.0154** | 0.4033 |
|  | n | 38 | 39 | 37 | 37 | 37 | 37 |
| avL_F4 | r | -0.05216 | 0.22731 | 0.14721 | **-0.45735** | -0.32457 | -0.17169 |
|  | p | 0.7558 | 0.1640 | 0.3846 | **0.0044** | 0.0500 | 0.3096 |
|  | n | 38 | 39 | 37 | 37 | 37 | 37 |
| avA_F4 | r | -0.02431 | 0.00275 | 0.09581 | **-0.33705** | -0.28858 | -0.13120 |
|  | p | 0.8848 | 0.9867 | 0.5727 | **0.0414** | 0.0832 | 0.4389 |
|  | n | 38 | 39 | 37 | 37 | 37 | 37 |
| avF_F4 | r | -0.12715 | -0.15666 | 0.21758 | 0.01757 | -0.06497 | 0.10415 |
|  | p | 0.4468 | 0.3409 | 0.1958 | 0.9178 | 0.7024 | 0.5396 |
|  | n | 38 | 39 | 37 | 37 | 37 | 37 |
| avD_F4 | r | -0.04680 | 0.16395 | 0.09045 | **-0.54879** | **-0.39708** | -0.18621 |
|  | p | 0.7802 | 0.3186 | 0.5945 | **0.0004** | **0.0150** | 0.2698 |
|  | n | 38 | 39 | 37 | 37 | 37 | 37 |
| avL_P3 | r | 0.06839 | 0.10705 | 0.12058 | -0.26762 | -0.27714 | -0.01715 |
|  | p | 0.6875 | 0.5223 | 0.4836 | 0.1146 | 0.1018 | 0.9209 |
|  | n | 37 | 38 | 36 | 36 | 36 | 36 |
| avA_P3 | r | -0.00128 | 0.01016 | 0.15515 | -0.22530 | -0.18877 | -0.08219 |
|  | p | 0.9940 | 0.9517 | 0.3663 | 0.1865 | 0.2702 | 0.6337 |
|  | n | 37 | 38 | 36 | 36 | 36 | 36 |
| avF_P3 | r | 0.02845 | 0.05048 | -0.07261 | 0.31351 | 0.16943 | 0.25293 |
|  | p | 0.8672 | 0.7634 | 0.6739 | 0.0626 | 0.3232 | 0.1367 |
|  | n | 37 | 38 | 36 | 36 | 36 | 36 |
| avD_P3 | r | 0.02313 | 0.15702 | 0.21446 | -0.08836 | -0.06303 | -0.03636 |
|  | p | 0.8904 | 0.3398 | 0.2024 | 0.6030 | 0.7109 | 0.8308 |
|  | n | 38 | 39 | 37 | 37 | 37 | 37 |
| avL_P4 | r | 0.00392 | 0.10521 | 0.21674 | -0.30220 | -0.17308 | -0.24849 |
|  | p | 0.9814 | 0.5239 | 0.1976 | 0.0691 | 0.3056 | 0.1381 |
|  | n | 38 | 39 | 37 | 37 | 37 | 37 |
| avA_P4 | r | 0.00675 | -0.01502 | 0.22487 | -0.19300 | -0.22409 | 0.00756 |
|  | p | 0.9679 | 0.9277 | 0.1809 | 0.2524 | 0.1824 | 0.9646 |
|  | n | 38 | 39 | 37 | 37 | 37 | 37 |
| avF_P4 | r | 0.10847 | 0.12399 | -0.03678 | 0.26481 | 0.16596 | 0.15988 |
|  | p | 0.5168 | 0.4520 | 0.8289 | 0.1132 | 0.3263 | 0.3445 |
|  | n | 38 | 39 | 37 | 37 | 37 | 37 |
| avD_P4 | r | -0.04337 | 0.16066 | **0.36608** | -0.11551 | -0.16490 | 0.04956 |
|  | p | 0.7960 | 0.3285 | **0.0259** | 0.4960 | 0.3294 | 0.7708 |
|  | n | 38 | 39 | 37 | 37 | 37 | 37 |

avL = averaged spindle length; avA = averaged spindle amplitude; avF = averaged spindle frequency; avD = averaged spindle density

# **Table S12.** Correlations (Pearson r) of sleep spindle parameters (length = duration, amplitude, frequency, density) in stage NREM at four electrode sites (F3, F4, P3, P4) with plasma concentration of six AD-biomarkers collected in the morning. Statistically significant correlations are shown in bold.

| **morning** |  | **p-tau217** | **p-tau181** | **t-tau** | **Aß42** | **Aß40** | **Aß42/40** |
| --- | --- | --- | --- | --- | --- | --- | --- |
| avL_F3 | r | -0.09168 | 0.23246 | 0.21343 | **-0.61968** | **-0.51225** | -0.00970 |
|  | p | 0.5841 | 0.1544 | 0.2047 | **<0.0001** | **0.0012** | 0.9546 |
|  | n | 38 | 39 | 37 | 37 | 37 | 37 |
| avA_F3 | r | -0.01911 | -0.09175 | -0.14610 | -0.26877 | -0.19400 | -0.20619 |
|  | p | 0.9093 | 0.5785 | 0.3882 | 0.1077 | 0.2499 | 0.2208 |
|  | n | 38 | 39 | 37 | 37 | 37 | 37 |
| avF_F3 | r | -0.16109 | -0.13731 | -0.12799 | -0.06050 | -0.13921 | 0.03535 |
|  | p | 0.3340 | 0.4045 | 0.4503 | 0.7220 | 0.4112 | 0.8355 |
|  | n | 38 | 39 | 37 | 37 | 37 | 37 |
| avD_F3 | r | -0.01166 | 0.19618 | 0.16094 | **-0.57742** | **-0.49459** | 0.00875 |
|  | p | 0.9438 | 0.2250 | 0.3344 | **0.0001** | **0.0016** | 0.9584 |
|  | n | 39 | 40 | 38 | 38 | 38 | 38 |
| avL_F4 | r | -0.13442 | 0.14525 | 0.07943 | **-0.56138** | **-0.42452** | -0.13141 |
|  | p | 0.4146 | 0.3712 | 0.6355 | **0.0002** | **0.0079** | 0.4316 |
|  | n | 39 | 40 | 38 | 38 | 38 | 38 |
| avA_F4 | r | -0.05206 | -0.10920 | -0.14722 | -0.30292 | -0.22643 | -0.20029 |
|  | p | 0.7529 | 0.5024 | 0.3778 | 0.0645 | 0.1716 | 0.2279 |
|  | n | 39 | 40 | 38 | 38 | 38 | 38 |
| avF_F4 | r | -0.24530 | -0.15955 | 0.00627 | -0.14617 | -0.22239 | 0.09436 |
|  | p | 0.1323 | 0.3254 | 0.9702 | 0.3812 | 0.1796 | 0.5731 |
|  | n | 39 | 40 | 38 | 38 | 38 | 38 |
| avD_F4 | r | -0.10826 | 0.11730 | 0.04376 | **-0.61076** | **-0.47758** | -0.12075 |
|  | p | 0.5118 | 0.4710 | 0.7942 | **<0.0001** | **0.0024** | 0.4702 |
|  | n | 39 | 40 | 38 | 38 | 38 | 38 |
| avL_P3 | r | -0.04610 | 0.03830 | -0.01642 | -0.24921 | -0.24505 | -0.04654 |
|  | p | 0.7834 | 0.8170 | 0.9231 | 0.1369 | 0.1438 | 0.7844 |
|  | n | 38 | 39 | 37 | 37 | 37 | 37 |
| avA_P3 | r | 0.00110 | -0.07101 | -0.15556 | -0.13844 | -0.12246 | -0.14010 |
|  | p | 0.9948 | 0.6675 | 0.3579 | 0.4139 | 0.4703 | 0.4082 |
|  | n | 38 | 39 | 37 | 37 | 37 | 37 |
| avF_P3 | r | 0.05970 | 0.14959 | 0.08537 | **0.33861** | 0.17731 | 0.28319 |
|  | p | 0.7218 | 0.3634 | 0.6154 | **0.0404** | 0.2938 | 0.0894 |
|  | n | 38 | 39 | 37 | 37 | 37 | 37 |
| avD_P3 | r | -0.01697 | 0.08790 | 0.00393 | -0.17547 | -0.19311 | 0.00306 |
|  | p | 0.9183 | 0.5896 | 0.9813 | 0.2920 | 0.2454 | 0.9855 |
|  | n | 39 | 40 | 38 | 38 | 38 | 38 |
| avL_P4 | r | -0.11018 | 0.01927 | -0.01445 | -0.26936 | -0.29033 | -0.04842 |
|  | p | 0.5043 | 0.9061 | 0.9314 | 0.1020 | 0.0770 | 0.7728 |
|  | n | 39 | 40 | 38 | 38 | 38 | 38 |
| avA_P4 | r | 0.00509 | -0.10600 | -0.12625 | -0.14952 | -0.14924 | -0.13139 |
|  | p | 0.9755 | 0.5151 | 0.4501 | 0.3703 | 0.3712 | 0.4317 |
|  | n | 39 | 40 | 38 | 38 | 38 | 38 |
| avF_P4 | r | 0.11966 | 0.21120 | 0.08982 | **0.38287** | 0.21473 | 0.27170 |
|  | p | 0.4681 | 0.1908 | 0.5918 | **0.0177** | 0.1954 | 0.0989 |
|  | n | 39 | 40 | 38 | 38 | 38 | 38 |
| avD_P4 | r | -0.07433 | 0.04250 | -0.00451 | -0.19230 | -0.23773 | -0.01706 |
|  | p | 0.6529 | 0.7946 | 0.9786 | 0.2474 | 0.1507 | 0.9190 |
|  | n | 39 | 40 | 38 | 38 | 38 | 38 |

avL = averaged spindle length; avA = averaged spindle amplitude; avF = averaged spindle frequency; avD = averaged spindle density

# **Table S13** Correlations (Pearson r) of sleep spindle parameters (length = duration, amplitude, frequency, density) in stage N2 at four electrode sites (F3, F4, P3, P4) with overnight changes in plasma concentration of six AD-biomarkers. Statistically significant correlations are shown in bold.

| **Overnight changes** |  | **p-tau217** | **p-tau181** | **t-tau** | **Aß42** | **Aß40** | **Aß42/40** |
| --- | --- | --- | --- | --- | --- | --- | --- |
| avL_F3 | r | 0.06281 | 0.26421 | 0.24655 | -0.12540 | -0.19084 | 0.22140 |
|  | p | 0.7079 | 0.1041 | 0.1413 | 0.4596 | 0.2579 | 0.1879 |
|  | n | 38 | 39 | 37 | 37 | 37 | 37 |
| avA_F3 | r | 0.01239 | 0.01679 | -0.08882 | -0.01095 | 0.03709 | -0.22371 |
|  | p | 0.9412 | 0.9192 | 0.6011 | 0.9487 | 0.8275 | 0.1832 |
|  | n | 38 | 39 | 37 | 37 | 37 | 37 |
| avF_F3 | r | 0.01923 | 0.13533 | -0.09425 | 0.01329 | 0.03498 | -0.21228 |
|  | p | 0.9088 | 0.4114 | 0.5790 | 0.9378 | 0.8372 | 0.2072 |
|  | n | 38 | 39 | 37 | 37 | 37 | 37 |
| avD_F3 | r | -0.00154 | 0.27848 | 0.28935 | -0.12800 | -0.12848 | 0.18225 |
|  | p | 0.9927 | 0.0860 | 0.0824 | 0.4502 | 0.4486 | 0.2803 |
|  | n | 38 | 39 | 37 | 37 | 37 | 37 |
| avL_F4 | r | 0.09706 | 0.28258 | 0.10745 | -0.01837 | -0.07356 | 0.02834 |
|  | p | 0.5566 | 0.0773 | 0.5208 | 0.9128 | 0.6607 | 0.8659 |
|  | n | 39 | 40 | 38 | 38 | 38 | 38 |
| avA_F4 | r | 0.05297 | 0.04398 | -0.08453 | 0.01496 | 0.02117 | -0.16197 |
|  | p | 0.7488 | 0.7876 | 0.6139 | 0.9289 | 0.8996 | 0.3313 |
|  | n | 39 | 40 | 38 | 38 | 38 | 38 |
| avF_F4 | r | -0.07389 | 0.08186 | 0.00557 | -0.10406 | -0.02478 | -0.15696 |
|  | p | 0.6548 | 0.6156 | 0.9735 | 0.5341 | 0.8826 | 0.3467 |
|  | n | 39 | 40 | 38 | 38 | 38 | 38 |
| avD_F4 | r | 0.01161 | 0.24130 | 0.16563 | -0.05211 | -0.08161 | 0.08350 |
|  | p | 0.9441 | 0.1336 | 0.3203 | 0.7560 | 0.6262 | 0.6182 |
|  | n | 39 | 40 | 38 | 38 | 38 | 38 |
| avL_P3 | r | 0.09994 | 0.25980 | 0.04195 | 0.02317 | 0.20303 | -0.31764 |
|  | p | 0.5505 | 0.1102 | 0.8053 | 0.8917 | 0.2281 | 0.0554 |
|  | n | 38 | 39 | 37 | 37 | 37 | 37 |
| avA_P3 | r | 0.01578 | -0.03705 | -0.11335 | -0.07381 | 0.01028 | -0.25685 |
|  | p | 0.9251 | 0.8228 | 0.5042 | 0.6642 | 0.9518 | 0.1249 |
|  | n | 38 | 39 | 37 | 37 | 37 | 37 |
| avF_P3 | r | 0.05560 | 0.24366 | 0.17236 | 0.06832 | 0.13398 | 0.02201 |
|  | p | 0.7402 | 0.1350 | 0.3077 | 0.6878 | 0.4292 | 0.8971 |
|  | n | 38 | 39 | 37 | 37 | 37 | 37 |
| avD_P3 | r | 0.12527 | 0.25286 | 0.06832 | -0.03035 | 0.10288 | -0.20660 |
|  | p | 0.4474 | 0.1154 | 0.6836 | 0.8565 | 0.5388 | 0.2133 |
|  | n | 39 | 40 | 38 | 38 | 38 | 38 |
| avL_P4 | r | 0.06855 | 0.21990 | 0.02805 | 0.01461 | 0.16747 | -0.26767 |
|  | p | 0.6784 | 0.1727 | 0.8673 | 0.9306 | 0.3149 | 0.1042 |
|  | n | 39 | 40 | 38 | 38 | 38 | 38 |
| avA_P4 | r | -0.02887 | -0.09764 | -0.11153 | -0.09774 | -0.07744 | -0.14773 |
|  | p | 0.8615 | 0.5489 | 0.5050 | 0.5594 | 0.6440 | 0.3761 |
|  | n | 39 | 40 | 38 | 38 | 38 | 38 |
| avF_P4 | r | 0.06856 | 0.22831 | 0.11392 | 0.10401 | 0.21031 | -0.05702 |
|  | p | 0.6784 | 0.1565 | 0.4959 | 0.5343 | 0.2050 | 0.7338 |
|  | n | 39 | 40 | 38 | 38 | 38 | 38 |
| avD_P4 | r | 0.01687 | 0.14687 | -0.02290 | -0.06878 | 0.04678 | -0.24252 |
|  | p | 0.9188 | 0.3658 | 0.8915 | 0.6816 | 0.7803 | 0.1424 |
|  | n | 39 | 40 | 38 | 38 | 38 | 38 |

avL = averaged spindle length; avA = averaged spindle amplitude; avF = averaged spindle frequency; avD = averaged spindle density

# **Table S14.** Correlations (Pearson r) of sleep spindle parameters (length = duration, amplitude, frequency, density) in stage N3 at four electrode sites (F3, F4, P3, P4) with overnight changes in plasma concentration of six AD-biomarkers. Statistically significant correlations are shown in bold.

| **Overnight changes** |  | **p-tau217** | **p-tau181** | **t-tau** | **Aß42** | **Aß40** | **Aß42/40** |
| --- | --- | --- | --- | --- | --- | --- | --- |
| avL_F3 | r | 0.14357 | **0.35654** | 0.25117 | 0.03833 | -0.02722 | 0.09315 |
|  | p | 0.3966 | **0.0280** | 0.1395 | 0.8243 | 0.8748 | 0.5889 |
|  | n | 37 | 38 | 36 | 36 | 36 | 36 |
| avA_F3 | r | 0.01426 | 0.07363 | 0.14520 | -0.08510 | -0.04465 | -0.09699 |
|  | p | 0.9332 | 0.6604 | 0.3981 | 0.6217 | 0.7960 | 0.5736 |
|  | n | 37 | 38 | 36 | 36 | 36 | 36 |
| avF_F3 | r | -0.05939 | -0.04976 | 0.15918 | -0.15938 | -0.11898 | -0.03427 |
|  | p | 0.7270 | 0.7667 | 0.3538 | 0.3532 | 0.4895 | 0.8427 |
|  | n | 37 | 38 | 36 | 36 | 36 | 36 |
| avD_F3 | r | 0.15512 | **0.31840** | 0.31752 | 0.05947 | -0.02933 | 0.12980 |
|  | p | 0.3524 | **0.0482** | 0.0555 | 0.7266 | 0.8632 | 0.4439 |
|  | n | 38 | 39 | 37 | 37 | 37 | 37 |
| avL_F4 | r | 0.14715 | **0.34176** | 0.24046 | 0.05366 | 0.07882 | -0.07648 |
|  | p | 0.3780 | **0.0332** | 0.1517 | 0.7524 | 0.6429 | 0.6528 |
|  | n | 38 | 39 | 37 | 37 | 37 | 37 |
| avA_F4 | r | 0.04137 | 0.07870 | 0.13390 | -0.02215 | -0.02458 | -0.05823 |
|  | p | 0.8052 | 0.6339 | 0.4295 | 0.8965 | 0.8852 | 0.7321 |
|  | n | 38 | 39 | 37 | 37 | 37 | 37 |
| avF_F4 | r | -0.02753 | -0.01582 | 0.18162 | -0.10410 | -0.09704 | 0.03737 |
|  | p | 0.8697 | 0.9239 | 0.2820 | 0.5398 | 0.5678 | 0.8262 |
|  | n | 38 | 39 | 37 | 37 | 37 | 37 |
| avD_F4 | r | 0.11614 | 0.26644 | 0.20250 | 0.05799 | 0.01614 | -0.01258 |
|  | p | 0.4874 | 0.1011 | 0.2294 | 0.7332 | 0.9244 | 0.9411 |
|  | n | 38 | 39 | 37 | 37 | 37 | 37 |
| avL_P3 | r | 0.10440 | 0.22453 | 0.25754 | -0.08806 | 0.04145 | -0.20003 |
|  | p | 0.5386 | 0.1753 | 0.1294 | 0.6095 | 0.8103 | 0.2421 |
|  | n | 37 | 38 | 36 | 36 | 36 | 36 |
| avA_P3 | r | 0.02069 | 0.02007 | 0.14576 | -0.14653 | -0.04933 | -0.16385 |
|  | p | 0.9032 | 0.9048 | 0.3963 | 0.3938 | 0.7751 | 0.3396 |
|  | n | 37 | 38 | 36 | 36 | 36 | 36 |
| avF_P3 | r | 0.00950 | 0.16069 | 0.12615 | 0.04444 | 0.07706 | -0.00244 |
|  | p | 0.9555 | 0.3352 | 0.4635 | 0.7969 | 0.6551 | 0.9887 |
|  | n | 37 | 38 | 36 | 36 | 36 | 36 |
| avD_P3 | r | 0.17204 | 0.31429 | 0.23625 | 0.00315 | 0.12970 | -0.17654 |
|  | p | 0.3017 | 0.0513 | 0.1592 | 0.9853 | 0.4442 | 0.2959 |
|  | n | 38 | 39 | 37 | 37 | 37 | 37 |
| avL_P4 | r | 0.07845 | 0.30469 | **0.34258** | -0.02173 | 0.03356 | -0.14193 |
|  | p | 0.6397 | 0.0593 | **0.0379** | 0.8984 | 0.8437 | 0.4021 |
|  | n | 38 | 39 | 37 | 37 | 37 | 37 |
| avA_P4 | r | -0.01560 | -0.05652 | 0.13992 | -0.19781 | -0.15801 | -0.00844 |
|  | p | 0.9259 | 0.7326 | 0.4088 | 0.2406 | 0.3503 | 0.9604 |
|  | n | 38 | 39 | 37 | 37 | 37 | 37 |
| avF_P4 | r | -0.06272 | 0.05193 | 0.22663 | -0.03405 | -0.08877 | 0.14151 |
|  | p | 0.7084 | 0.7536 | 0.1774 | 0.8414 | 0.6013 | 0.4035 |
|  | n | 38 | 39 | 37 | 37 | 37 | 37 |
| avD_P4 | r | 0.08898 | 0.26846 | 0.20620 | -0.03286 | 0.01637 | -0.07435 |
|  | p | 0.5952 | 0.0984 | 0.2208 | 0.8469 | 0.9234 | 0.6619 |
|  | n | 38 | 39 | 37 | 37 | 37 | 37 |

avL = averaged spindle length; avA = averaged spindle amplitude; avF = averaged spindle frequency; avD = averaged spindle density

# **Table S15.** Correlations (Pearson r) of sleep spindle parameters (length = duration, amplitude, frequency, density) in NREM at four electrode sites (F3, F4, P3, P4) with overnight changes in plasma concentration of six AD-biomarkers. Statistically significant correlations are shown in bold.

| **Overnight changes** |  | **p-tau217** | **p-tau181** | **t-tau** | **Aß42** | **Aß40** | **Aß42/40** |
| --- | --- | --- | --- | --- | --- | --- | --- |
| avL_F3 | r | 0.10863 | **0.32052** | 0.24839 | -0.07844 | -0.15081 | 0.20683 |
|  | p | 0.5162 | **0.0467** | 0.1382 | 0.6445 | 0.3730 | 0.2194 |
|  | n | 38 | 39 | 37 | 37 | 37 | 37 |
| avA_F3 | r | 0.02871 | 0.02500 | -0.09475 | 0.00219 | 0.04083 | -0.21973 |
|  | p | 0.8641 | 0.8799 | 0.5770 | 0.9898 | 0.8104 | 0.1913 |
|  | n | 38 | 39 | 37 | 37 | 37 | 37 |
| avF_F3 | r | -0.01229 | 0.05505 | -0.06715 | -0.05845 | -0.05893 | -0.10958 |
|  | p | 0.9416 | 0.7392 | 0.6929 | 0.7311 | 0.7290 | 0.5185 |
|  | n | 38 | 39 | 37 | 37 | 37 | 37 |
| avD_F3 | r | 0.11778 | **0.32846** | 0.26829 | -0.02653 | -0.14525 | 0.25030 |
|  | p | 0.4752 | **0.0385** | 0.1034 | 0.8744 | 0.3842 | 0.1296 |
|  | n | 39 | 40 | 38 | 38 | 38 | 38 |
| avL_F4 | r | 0.12432 | 0.31036 | 0.11510 | 0.02211 | -0.00631 | -0.00451 |
|  | p | 0.4508 | 0.0513 | 0.4914 | 0.8952 | 0.9700 | 0.9786 |
|  | n | 39 | 40 | 38 | 38 | 38 | 38 |
| avA_F4 | r | 0.06211 | 0.04964 | -0.08338 | 0.02994 | 0.03094 | -0.16225 |
|  | p | 0.7072 | 0.7610 | 0.6187 | 0.8584 | 0.8537 | 0.3304 |
|  | n | 39 | 40 | 38 | 38 | 38 | 38 |
| avF_F4 | r | -0.05712 | 0.04488 | 0.03712 | -0.13141 | -0.10167 | -0.03674 |
|  | p | 0.7298 | 0.7833 | 0.8249 | 0.4316 | 0.5436 | 0.8266 |
|  | n | 39 | 40 | 38 | 38 | 38 | 38 |
| avD_F4 | r | 0.06048 | 0.27339 | 0.11687 | -0.00700 | -0.05660 | 0.04052 |
|  | p | 0.7146 | 0.0878 | 0.4847 | 0.9667 | 0.7357 | 0.8091 |
|  | n | 39 | 40 | 38 | 38 | 38 | 38 |
| avL_P3 | r | 0.11022 | 0.21651 | 0.04666 | 0.01468 | 0.17354 | -0.27703 |
|  | p | 0.5101 | 0.1855 | 0.7839 | 0.9313 | 0.3043 | 0.0969 |
|  | n | 38 | 39 | 37 | 37 | 37 | 37 |
| avA_P3 | r | 0.01535 | -0.04353 | -0.11409 | -0.07335 | 0.01134 | -0.25711 |
|  | p | 0.9271 | 0.7924 | 0.5014 | 0.6661 | 0.9469 | 0.1245 |
|  | n | 38 | 39 | 37 | 37 | 37 | 37 |
| avF_P3 | r | 0.04494 | 0.23923 | 0.18913 | 0.06905 | 0.15228 | 0.02000 |
|  | p | 0.7888 | 0.1424 | 0.2623 | 0.6847 | 0.3682 | 0.9065 |
|  | n | 38 | 39 | 37 | 37 | 37 | 37 |
| avD_P3 | r | 0.14005 | 0.26482 | 0.07574 | -0.00882 | 0.09890 | -0.17958 |
|  | p | 0.3951 | 0.0987 | 0.6513 | 0.9581 | 0.5547 | 0.2807 |
|  | n | 39 | 40 | 38 | 38 | 38 | 38 |
| avL_P4 | r | 0.04788 | 0.19126 | 0.01835 | 0.01785 | 0.15420 | -0.25843 |
|  | p | 0.7722 | 0.2371 | 0.9129 | 0.9153 | 0.3553 | 0.1172 |
|  | n | 39 | 40 | 38 | 38 | 38 | 38 |
| avA_P4 | r | -0.02496 | -0.09876 | -0.11198 | -0.09553 | -0.07162 | -0.15005 |
|  | p | 0.8801 | 0.5443 | 0.5033 | 0.5683 | 0.6692 | 0.3685 |
|  | n | 39 | 40 | 38 | 38 | 38 | 38 |
| avF_P4 | r | 0.03665 | 0.19856 | 0.15236 | 0.10619 | 0.19954 | -0.01940 |
|  | p | 0.8247 | 0.2194 | 0.3611 | 0.5257 | 0.2297 | 0.9080 |
|  | n | 39 | 40 | 38 | 38 | 38 | 38 |
| avD_P4 | r | 0.03736 | 0.15916 | -0.01617 | -0.04509 | 0.04201 | -0.20672 |
|  | p | 0.8213 | 0.3266 | 0.9232 | 0.7881 | 0.8022 | 0.2130 |
|  | n | 39 | 40 | 38 | 38 | 38 | 38 |

avL = averaged spindle length; avA = averaged spindle amplitude; avF = averaged spindle frequency; avD = averaged spindle density

# **Table S16.** Statistically significant correlations between power spectra of seven different frequency bands at 19 different electrode sites and AD biomarker concentrations collected in the evening are shown for A) entire night, B) stage N1, C) stage N2, D) stage N3, E) NREM sleep and F) stage R. Locations of statistically significant correlations are presented. Positive correlations are shown in bold, negative correlations in regular font.

|  |  | **Locations with statistically significant correlations** | | | | | |
| --- | --- | --- | --- | --- | --- | --- | --- |
|  |  | **p-tau217** | **p-tau181** | **t-tau** | **Aβ42** | **Aβ40** | **Aβ42/Aβ40** |
| **evening** |  |  |  |  |  |  |  |
| **A) entire night** | delta |  | Pz |  |  |  |  |
|  | theta |  |  |  |  |  |  |
|  | alpha |  |  |  | Fz, F4 | Fp1, Fp2  F3, Fz, F4, |  |
|  | beta1 |  |  |  |  | Fp1, Fp2  F3, Fz, F4 |  |
|  | beta2 |  |  |  |  | Fz, Fp2 |  |
|  | wide spindle frequency range |  |  |  | F4 | Fp1, Fp2  F3, Fz, F4 |  |
|  | narrow spindle frequency range |  |  |  |  | Fp1, Fp2  F7, F3, Fz, F4 |  |
| **B) stage N1** | delta |  | F7 |  |  |  |  |
|  | theta |  |  |  |  |  | O2 |
|  | alpha |  |  |  |  |  |  |
|  | beta1 |  |  |  |  | Fp1, Fp2  F3, Fz, F4 |  |
|  | beta2 |  |  |  |  | Fp2 |  |
|  | wide spindle frequency range |  |  |  |  | Fp1, Fp2  F3, Fz, F4 |  |
|  | narrow spindle frequency range |  |  |  |  | Fp1, Fp2  F3, Fz, F4 |  |
| **C) stage N2** | delta |  |  |  |  |  |  |
|  | theta |  |  |  |  |  |  |
|  | alpha |  |  |  | F3, Fz, F4 | Fz |  |
|  | beta1 |  |  |  |  | Fp1, Fp2  F7, F3, Fz, F4, F8 C3, Cz, C4 |  |
|  | beta2 |  |  |  |  | Fp2  Fz |  |
|  | wide spindle frequency range |  |  |  | F3, Fz, F4 | Fp1, Fp2  F7, F3, Fz, F4, F8 C3, Cz, C4 |  |
|  | narrow spindle frequency range |  |  |  | Fz, F4 | Fp1, Fp2  F7, F3, Fz, F4, F8 C3, C4 |  |

**Table S16 (continued)**. Statistically significant correlations between power spectra of seven different frequency bands at 19 different electrode sites and AD biomarker concentrations collected in the evening are shown for A) entire night, B) stage N1, C) stage N2, D) stage N3, E) NREM sleep and F) stage R. Locations of statistically significant correlations are presented. Positive correlations are shown in bold, negative correlations in regular font.

|  |  | **Locations with statistically significant correlations** | | | | | |
| --- | --- | --- | --- | --- | --- | --- | --- |
| **evening** |  | **p-tau217** | **p-tau181** | **t-tau** | **Aβ42** | **Aβ40** | **Aβ42/Aβ40** |
| **D) stage N3** | delta |  |  |  |  |  |  |
|  | theta |  |  |  |  |  |  |
|  | alpha |  |  |  | Fp1, Fp2  F3, Fz, F4 | Fp1, Fp2  F7, F3, Fz, F4 |  |
|  | beta1 |  |  |  | Fz, F4 | Fp1, Fp2  F7, F3, Fz, F4  C3, Cz, C4 |  |
|  | beta2 |  |  |  |  |  |  |
|  | wide spindle frequency range |  |  |  | Fp2  F3, Fz, F4  Cz | Fp1, Fp2  F7, F3, Fz, F4, F8 C3, Cz, C4 |  |
|  | narrow spindle frequency range |  |  |  | Fz, F4 | Fp1, Fp2  F7, F3, Fz, F4  C3, Cz, C4 |  |
| **E) NREM** | delta |  | Pz |  |  |  |  |
|  | theta |  |  |  |  |  |  |
|  | alpha |  |  |  | F3, Fz, F4 | Fp2  Fz |  |
|  | beta1 |  |  |  | F4 | Fp1, Fp2  F7, F3, Fz, F4  Cz, C4 |  |
|  | beta2 |  |  |  |  | Fp2  Fz |  |
|  | wide spindle frequency range |  |  |  | Fp2  F3, Fz, F4 | Fp1, Fp2  F7, F3, Fz, F4  Cz, C4 |  |
|  | narrow spindle frequency range |  |  |  | Fz, F4 | Fp1, Fp2  F7, F3, Fz, F4  C4 |  |
| **F) stage R** | delta |  |  |  |  |  |  |
|  | theta | Fz, F4 | Fp1, Fp2  F7, F3, Fz, F4 |  |  |  | O2 |
|  | alpha |  |  |  |  |  |  |
|  | beta1 |  |  |  |  |  |  |
|  | beta2 |  |  |  |  | Fz |  |
|  | wide spindle frequency range |  |  |  |  |  |  |
|  | narrow spindle frequency range |  |  |  |  |  |  |

# **Table S17.** Statistically significant correlations between power spectra of seven different frequency bands at 19 different electrode sites and AD biomarker concentrations collected in the morning are shown for A) entire night, B) stage N1, C) stage N2, D) stage N3, E) NREM sleep and F) stage R. Locations of statistically significant correlations are presented. Positive correlations are shown in bold, negative correlations in regular font.

|  |  | **Locations with statistically significant correlations** | | | | | |
| --- | --- | --- | --- | --- | --- | --- | --- |
| **morning** |  | **p-tau217** | **p-tau181** | **t-tau** | **Aβ42** | **Aβ40** | **Aβ42/Aβ40** |
| **A) Entire night** | delta |  |  |  |  |  |  |
|  | theta |  |  |  |  |  | F3, Fz, F4, F8  Cz  Pz, P4 |
|  | alpha |  |  |  |  |  |  |
|  | beta1 |  |  |  |  |  |  |
|  | beta2 |  |  |  |  |  |  |
|  | wide spindle frequency range |  |  |  |  |  |  |
|  | narrow spindle frequency range |  |  |  |  |  |  |
| **B) stage N1** | delta |  | Fp2  F8 |  |  |  | Fz |
|  | theta |  |  |  |  |  | F3, Fz, F4  Cz  Pz  O1, O2 |
|  | alpha |  |  |  |  |  |  |
|  | beta1 |  |  |  |  |  |  |
|  | beta2 |  |  |  |  |  |  |
|  | wide spindle frequency range |  |  |  |  |  |  |
|  | narrow spindle frequency range |  |  |  |  |  |  |
| **C) stage N2** | delta |  |  |  |  |  |  |
|  | theta |  |  |  |  |  |  |
|  | alpha |  |  |  | Fz, F4 |  |  |
|  | beta1 |  |  |  |  |  |  |
|  | beta2 |  |  |  |  |  |  |
|  | wide spindle frequency range |  |  |  | F3, Fz, F4 | Fz |  |
|  | narrow spindle frequency range |  |  |  | Fz,F4  Cz  Pz | Fz |  |

**Table S17 (continued)**. Statistically significant correlations between power spectra of seven different frequency bands at 19 different electrode sites and AD biomarker concentrations collected in the morning are shown for A) entire night, B) stage N1, C) stage N2, D) stage N3, E) NREM sleep and F) stage R. Locations of statistically significant correlations are presented. Positive correlations are shown in bold, negative correlations in regular font.

|  |  | **Locations with statistically significant correlations** | | | | | |
| --- | --- | --- | --- | --- | --- | --- | --- |
| **morning** |  | **p-tau217** | **p-tau181** | **t-tau** | **Aβ42** | **Aβ40** | **Aβ42/Aβ40** |
| **D) stage N3** | delta |  |  |  |  |  |  |
|  | theta |  |  |  |  |  |  |
|  | alpha |  |  |  | Fp1, Fp2  F7, F3, Fz, F4, F8 Cz | Fp1, Fp2 |  |
|  | beta1 |  |  |  | Fp2  F3, Fz, F4  Cz  Pz | Fp2  F3, Fz, F4  Cz, C4 |  |
|  | beta2 |  |  |  |  |  |  |
|  | wide spindle frequency range |  |  |  | Fp1, Fp2  F7, F3, Fz, F4, F8 T3, C3, Cz, C4, T4  Pz | Fp1, Fp2  F7, F3, Fz, F4, F8  C3, Cz, C4 |  |
|  | narrow spindle frequency range |  |  |  | Fp2  F3, Fz, F4  T3, C3, Cz, C4  Pz, P4 | Fp1, Fp2  F7, F3, Fz, F4 C3, Cz, C4  Pz, P4 |  |
| **E) NREM** | delta |  |  |  |  |  |  |
|  | theta |  |  |  |  |  | F3, Fz, F4 Cz  Pz |
|  | alpha |  |  |  | Fz |  |  |
|  | beta1 |  |  |  |  |  |  |
|  | beta2 |  |  |  |  |  |  |
|  | wide spindle frequency range |  |  |  | Fp1, Fp2  F3, Fz, F4 | Fz |  |
|  | narrow spindle frequency range |  |  |  | F3, Fz, F4  Cz  Pz | Fz |  |
| **F) stage R** | delta |  |  |  |  |  |  |
|  | theta |  |  |  |  |  | Pz |
|  | alpha |  |  |  |  |  |  |
|  | beta1 |  |  |  |  |  |  |
|  | beta2 |  |  |  |  |  |  |
|  | wide spindle frequency range |  |  |  |  |  |  |
|  | narrow spindle frequency range |  |  |  |  |  |  |

# **Table S18.** Statistically significant correlations between power spectra of seven different frequency bands at 19 different electrode sites and overnight changes in AD biomarker concentrations are shown for A) entire night, B) stage N1, C) stage N2, D) stage N3, E) E) NREM sleep and F) stage R. Locations of statistically significant correlations are presented. Positive correlations are shown in bold, negative correlations in regular font.

|  |  | **Locations with statistically significant correlations** | | | | | |
| --- | --- | --- | --- | --- | --- | --- | --- |
| **Overnight change** |  | **p-tau217** | **p-tau181** | **t-tau** | **Aβ42** | **Aβ40** | **Aβ42/Aβ40** |
| **A) Entire night** | delta |  |  |  |  |  |  |
|  | theta |  |  |  |  |  |  |
|  | alpha |  |  |  |  |  |  |
|  | beta1 |  |  |  |  |  |  |
|  | beta2 |  |  |  |  |  |  |
|  | wide spindle frequency range |  |  |  |  |  |  |
|  | narrow spindle frequency range |  |  |  |  |  |  |
| **B) stage N1** | delta |  |  |  |  |  |  |
|  | theta |  |  |  |  |  |  |
|  | alpha |  |  |  |  |  |  |
|  | beta1 |  |  |  |  |  |  |
|  | beta2 |  |  |  |  |  |  |
|  | wide spindle frequency range |  |  |  |  |  |  |
|  | narrow spindle frequency range |  |  |  |  |  |  |
| **C) stage N2** | delta |  |  |  |  |  |  |
|  | theta |  |  |  |  |  |  |
|  | alpha |  |  |  |  |  |  |
|  | beta1 |  |  |  |  |  | **F7**  **T3, C3,** **T4**  **P3** |
|  | beta2 |  |  |  |  |  | **F7**  **T3, C3, T4**  **T5, P3** |
|  | wide spindle frequency range |  |  |  |  |  | **T3, T4** |
|  | narrow spindle frequency range |  |  |  | **O1** |  | **F7**  **T3, T4** |

**Table S18 (continued)**. Statistically significant correlations between power spectra of seven different frequency bands at 19 different electrode sites and overnight changes in AD biomarker concentrations are shown for A) entire night, B) stage N1, C) stage N2, D) stage N3, E) NREM sleep and F) stage R sleep. Locations of statistically significant correlations are presented. Positive correlations are shown in bold, negative correlations in regular font.

|  |  | **Locations with statistically significant correlations** | | | | | | |
| --- | --- | --- | --- | --- | --- | --- | --- | --- |
| **Overnight change** |  | **p-tau217** | **p-tau181** | **t-tau** | **Aβ42** | **Aβ40** | **Aβ42/Aβ40** |  |
| **D) stage N3** | delta |  |  |  | **O2** |  |  |  |
|  | theta |  |  |  | **O1, O2** | **O1, O2** |  |  |
|  | alpha |  |  |  | **O1** |  |  |  |
|  | beta1 |  |  |  | **O1** |  |  |  |
|  | beta2 |  |  | **Fp2** |  |  |  |  |
|  | wide spindle frequency range |  |  | **F3** | **O1, O2** |  |  |  |
|  | narrow spindle frequency range |  |  |  | **O1, O2** |  |  |  |
| **E) NREM** | delta |  |  |  |  |  |  |  |
|  | theta |  |  |  |  |  |  |  |
|  | alpha |  |  |  |  |  |  |  |
|  | beta1 |  |  |  |  |  |  |  |
|  | beta2 |  |  |  |  |  |  |  |
|  | wide spindle frequency range |  |  |  |  |  |  |  |
|  | narrow spindle frequency range |  |  |  |  |  |  |  |
| **F) stage R** | delta |  |  |  |  |  |  |  |
|  | theta | O2 |  |  |  |  |  |  |
|  | alpha |  |  |  |  |  |  |  |
|  | beta1 |  |  |  |  |  |  |  |
|  | beta2 |  |  |  |  |  |  |  |
|  | wide spindle frequency range |  |  |  |  |  |  |  |
|  | narrow spindle frequency range |  |  |  |  |  | T6 |  |

**Table S19.** Significant Pearson correlations and adjusted linear regression results for subjective sleep quality and AD biomarkers. Regression models were adjusted for evening AD biomarker levels (n.a. for evening biomarker correlations), sex, age, and BMI. Statistically significant results are shown in bold.

| **Subjective sleep quality** | **AD biomarker** | **Sampling time** | **Pearson r** | **p** | **independent variable** | **n** | **DF model** | **DF error** | **F-value** | **model *p*** | **adjusted R-Square** | **Parameter estimate intercept** | **Intercept *p*** | **Parameter estimate subjective sleep quality *p*** | **Parameter estimate AD-Biomarker (evening) *p*** | **Parameter estimate Sex *p*** | **Parameter estimate Age *p*** | **Parameter estimate BMI *p*** |
| --- | --- | --- | --- | --- | --- | --- | --- | --- | --- | --- | --- | --- | --- | --- | --- | --- | --- | --- |
| Subjective sleep latency | Aβ42 | evening | 0.3652 | **0.0242** | SL | 38 | 4 | 33 | 3.57 | **0.0158** | 0.2177 | -31.4826 | 0.5606 | n.a. | 0.0699 | 0.2588 | **0.0488** | 0.1350 |
| (SL) | Aβ42 | morning | 0.3950 | **0.0171** | Aβ42 | 36 | 5 | 30 | 12.66 | **<0.0001** | 0.6249 | 1.3407 | 0.7011 | 0.5520 | **<0.0001** | 0.9498 | 0.6968 | 0.5313 |
|  | Aβ40 | morning | 0.3811 | **0.0218** | Aβ40 | 36 | 5 | 30 | 9.46 | **<0.0001** | 0.5471 | 179.9622 | 0.0625 | 0.3253 | **<0.0001** | 0.4074 | 0.7362 | 0.0801 |
| Subjective wake duration | t-tau | morning | 0.4405 | **0.0116** | t-tau | 32 | 5 | 26 | 3.38 | **0.0175** | 0.2772 | -2.6332 | 0.6379 | **0.0089** | **0.0157** | 0.7460 | 0.8851 | 0.4549 |
|  | Aβ40 | morning | -0.4321 | **0.0135** | Aβ40 | 32 | 5 | 26 | 14.44 | **<0.0001** | 0.6843 | 230.6865 | **0.0092** | **0.0249** | **<0.0001** | 0.8575 | 0.6536 | **0.0184** |
|  | Aβ42/40 | morning | 0.4109 | **0.0195** | Aβ42/40 | 32 | 5 | 26 | 7.93 | **0.0001** | 0.5277 | -0.0201 | 0.3803 | **0.0418** | **0.0002** | 0.4455 | 0.7296 | 0.0794 |
|  | t-tau | overnight change | 0.4728 | **0.0063** | t-tau | 32 | 5 | 26 | 2.04 | 0.1064 | 0.1433 | -2.6344 | 0.6377 | **0.0089** | 0.2013 | 0.7459 | 0.8852 | 0.4549 |
|  | Aβ42 | overnight change | -0.4241 | **0.0156** | Aβ42 | 32 | 5 | 26 | 5.07 | **0.0022** | 0.3964 | 4.1342 | 0.1171 | **0.0031** | **0.0047** | 0.1538 | 0.7429 | 0.0899 |

**Table S20.** Significant Pearson correlations and adjusted linear regression results for objective sleep quality and AD biomarkers. Regression models were adjusted for evening AD biomarker levels (n.a. for evening biomarker correlations), sex, age, and BMI. Statistically significant results are shown in bold.

| **Objective sleep quality** | **AD biomarker** | **Sampling time** | **Pearson r** | **p** | **Dependent variable** | **n** | **DF model** | **DF error** | **F-value** | **model *p*** | **adjusted R-Square** | **Parameter estimate intercept** | **Intercept *p*** | **Parameter estimate objective sleep quality *p*** | **Parameter estimate AD-Biomarker (evening) *p*** | **Parameter estimate Sex *p*** | **Parameter estimate Age *p*** | **Parameter estimate BMI *p*** |
| --- | --- | --- | --- | --- | --- | --- | --- | --- | --- | --- | --- | --- | --- | --- | --- | --- | --- | --- |
| ***Sleep continuity*** |  |  |  |  |  |  |  |  |  |  |  |  |  |  |  |  |  |  |
| WASO | t-tau | morning | 0.3762 | **0.0199** | t-tau | 38 | 5 | 32 | 3.06 | **0.0228** | 0.2178 | -1.1522 | 0.8097 | **0.0213** | **0.0157** | 0.6474 | 0.5218 | 0.3552 |
|  | t-tau | overnight change | 0.3319 | **0.0418** | t-tau | 38 | 5 | 32 | 1.57 | 0.1957 | 0.0719 | -1.1531 | 0.8095 | **0.0213** | 0.1175 | 0.6473 | 0.5219 | 0.3552 |
| WTSP | t-tau | morning | 0.3782 | **0.0192** | t-tau | 38 | 5 | 32 | 3.44 | **0.0134** | 0.2478 | -1.4336 | 0.7601 | **0.0106** | **0.0074** | 0.7270 | 0.5622 | 0.3639 |
|  | p-tau217 | overnight change | -0.3176 | **0.0488** | p-tau217 | 39 | 5 | 33 | 2.12 | 0.0878 | 0.1283 | -0.2268 | 0.5314 | **0.0484** | **0.0361** | 0.4696 | 0.1029 | 0.7057 |
|  | t-tau | overnight change | 0.3992 | **0.0130** | t-tau | 38 | 5 | 32 | 1.89 | 0.1234 | 0.1075 | -1.4344 | 0.7600 | **0.0106** | 0.1669 | 0.7268 | 0.5623 | 0.3639 |
| ***Latencies*** |  |  |  |  |  |  |  |  |  |  |  |  |  |  |  |  |  |  |
| LR | Aβ40 | overnight change | 0.3319 | **0.0418** | Aβ40 | 38 | 5 | 32 | 2.24 | 0.0743 | 0.1435 | 142.1501 | 0.1153 | 0.0596 | 0.2779 | 0.1818 | 0.7081 | 0.0745 |
| ***Sleep stages*** |  |  |  |  |  |  |  |  |  |  |  |  |  |  |  |  |  |  |
| stage N2 (%) | Aβ40 | morning | -0.3271 | **0.0450** | Aβ40 | 38 | 5 | 32 | 9.52 | **<0.0001** | 0.5351 | 195.8869 | **0.0483** | 0.3280 | **<0.0001** | 0.2720 | 0.8650 | 0.1307 |
| stage N3 (min) | p-tau181 | evening | -0.3502 | **0.0267** | stage N3 (min) | 40 | 4 | 35 | 5.07 | **0.0025** | 0.2947 | 104.2766 | 0.1978 | n.a. | 0.0915 | **0.0086** | 0.5873 | 0.2571 |
|  | t-tau | evening | -0.3425 | **0.0305** | stage N3 (min) | 40 | 4 | 35 | 5.89 | **0.0010** | 0.3340 | 153.7230 | **0.0478** | n.a. | **0.0280** | **0.0040** | 0.7981 | 0.2373 |
|  | t-tau | morning | -0.3543 | **0.0291** | t-tau | 38 | 5 | 32 | 2.23 | 0.0755 | 0.1424 | 1.8649 | 0.7247 | 0.1211 | 0.0523 | 0.3552 | 0.8267 | 0.7557 |
|  |  |  |  |  |  |  |  |  |  |  |  |  |  |  |  |  |  |  |
| stage N3 (%) | p-tau181 | evening | -0.3260 | **0.0401** | stage N3 (%) | 40 | 4 | 35 | 4.74 | **0.0037** | 0.2772 | 23.4185 | 0.2463 | n.a. | 0.0999 | **0.0142** | 0.3952 | 0.2026 |
|  | t-tau | evening | -0.3808 | **0.0154** | stage N3 (%) | 40 | 4 | 35 | 6.11 | **0.0008** | 0.3438 | 35.9464 | 0.0585 | n.a. | **0.0140** | **0.0061** | 0.9142 | 0.1852 |
|  | t-tau | morning | -0.3675 | **0.0232** | t-tau | 38 | 5 | 32 | 2.21 | 0.0781 | 0.1401 | 1.6164 | 0.7587 | 0.1280 | 0.0631 | 0.3723 | 0.9201 | 0.7874 |
| ***Awakenings and stage shifts*** |  |  |  |  |  |  |  |  |  |  |  |  |  |  |  |  |  |  |
| FW | Aβ40 | overnight change | 0.3232 | **0.0478** | Aβ40 | 38 | 5 | 32 | 2.31 | 0.0673 | 0.1502 | 105.0979 | 0.2604 | 0.0514 | 0.1015 | 0.7669 | 0.8377 | 0.0547 |
| FS | Aβ42/40 | evening | -0.3226 | **0.0423** | FS | 40 | 4 | 35 | 1.07 | 0.3837 | 0.0076 | 263.7625 | **0.0353** | n.a. | 0.0525 | 0.9332 | 0.6657 | 0.8573 |
|  | t-tau | morning | -0.4205 | **0.0086** | t-tau | 38 | 5 | 32 | 3.33 | **0.0157** | 0.2392 | 2.2876 | 0.6387 | **0.0129** | **0.0121** | 0.8842 | 0.9286 | 0.5210 |
|  | Aβ42/40 | morning | -0.4287 | **0.0072** | Aβ42/40 | 38 | 5 | 32 | 9.47 | **<0.0001** | 0.5337 | -0.0059 | 0.7704 | 0.0605 | **<0.0001** | 0.5119 | 0.6961 | **0.0492** |
|  | t-tau | overnight change | -0.3909 | **0.0152** | t-tau | 38 | 5 | 32 | 1.80 | 0.1415 | 0.0973 | 2.2864 | 0.6389 | **0.0129** | 0.1255 | 0.8841 | 0.9286 | 0.5210 |
|  | Aβ42 | overnight change | 0.3605 | **0.0262** | Aβ42 | 38 | 5 | 32 | 1.82 | 0.1371 | 0.0997 | -0.6971 | 0.8305 | 0.0542 | 0.0995 | 0.8519 | 0.5018 | 0.3902 |
| FSTST | Aβ42/40 | evening | -0.3180 | **0.0455** | FSTST | 40 | 4 | 35 | 0.99 | 0.4237 | -0.0006 | 38.0624 | **0.0458** | n.a. | 0.0795 | 0.9036 | 0.8916 | 0.9399 |
|  | t-tau | morning | -0.3897 | **0.0156** | t-tau | 38 | 5 | 32 | 3.02 | **0.0243** | 0.2141 | 1.8244 | 0.7112 | **0.0232** | **0.0114** | 0.9077 | 0.9605 | 0.5949 |
|  | Aβ42/40 | morning | -0.4279 | **0.0074** | Aβ42/40 | 38 | 5 | 32 | 9.44 | **<0.0001** | 0.5327 | -0.0076 | 0.7059 | 0.0629 | **<0.0001** | 0.5359 | 0.5895 | 0.0607 |
|  | p-tau181 | overnight change | -0.3483 | **0.0277** | p-tau181 | 40 | 5 | 34 | 6.72 | **0.0002** | 0.4231 | -28.8047 | 0.3375 | 0.0565 | **<0.0001** | 0.4883 | 0.2343 | 0.1047 |
|  | t-tau | overnight change | -0.3649 | **0.0243** | t-tau | 38 | 5 | 32 | 1.54 | 0.2065 | 0.0675 | 1.8232 | 0.7114 | **0.0232** | 0.1464 | 0.9075 | 0.9604 | 0.5948 |
|  | Aβ42 | overnight change | 0.3505 | **0.0310** | Aβ42 | 38 | 5 | 32 | 1.64 | 0.1791 | 0.0791 | -0.4862 | 0.8824 | 0.0836 | 0.1192 | 0.8403 | 0.5832 | 0.4377 |

**Table S21.** Significant Pearson correlations and adjusted linear regression results for sleep spindle variables in stage N2 and AD biomarkers. Regression models were adjusted for evening AD biomarker levels (n.a. for evening biomarker correlations), sex, age, and BMI. Statistically significant results are shown in bold.

| **Spindle parameter Stage N2** | | **AD biomarker** | **Sampling time** | **Pearson r** | **p** | **Dependent variable** | **n** | **DF model** | **DF error** | **F-value** | **model *p*** | **adjusted R-Square** | **Parameter estimate intercept** | **Intercept *p*** | **Parameter estimate spindle parameter *p*** | **Parameter estimate AD-Biomarker (evening) *p*** | **Parameter estimate Sex *p*** | **Parameter estimate Age *p*** | **Parameter estimate BMI *p*** |
| --- | --- | --- | --- | --- | --- | --- | --- | --- | --- | --- | --- | --- | --- | --- | --- | --- | --- | --- | --- |
| **F3** |  |  |  |  |  |  |  |  |  |  |  |  |  |  |  |  |  |  |  |
| length | avL_F3 | Aβ42 | evening | -0.5305 | **0.0005** | avL_F3 | 39 | 4 | 34 | 4.41 | **0.0056** | 0.2639 | 0.8498 | **<0.0001** | n.a. | **0.0009** | 0.8980 | 0.9027 | 0.1022 |
|  | avL_F3 | Aβ40 | evening | -0.4121 | **0.0091** | avL_F3 | 39 | 4 | 34 | 2.86 | **0.0380** | 0.1639 | 0.7651 | **<0.0001** | n.a. | **0.0091** | 0.9339 | 0.3263 | 0.1940 |
|  | avL_F3 | Aβ42 | morning | -0.6123 | **<0.0001** | Aβ42 | 37 | 5 | 31 | 14.99 | **<0.0001** | 0.6603 | 7.9372 | 0.1155 | 0.0699 | **<0.0001** | 0.7966 | 0.5217 | 0.8584 |
|  | avL_F3 | Aβ40 | morning | -0.5153 | **0.0011** | Aβ40 | 37 | 5 | 31 | 10.37 | **<0.0001** | 0.5655 | 294.6017 | **0.0237** | 0.1484 | **0.0002** | 0.4797 | 0.8873 | 0.0950 |
| frequency | avF_F3 | p-tau181 | evening | -0.3223 | **0.0454** | avF_F3 | 39 | 4 | 34 | 9.27 | **<0.0001** | 0.4654 | 11.5845 | **<0.0001** | n.a. | 0.6629 | 0.1374 | **<0.0001** | **0.0058** |
| density | avD_F3 | Aβ42 | evening | -0.5032 | **0.0011** | avD_F3 | 39 | 4 | 34 | 4.72 | **0.0039** | 0.2816 | 3.4309 | 0.1086 | n.a. | **0.0020** | 0.2792 | 0.2551 | 0.0610 |
|  | avD_F3 | Aβ40 | evening | -0.5345 | **0.0004** | avD_F3 | 39 | 4 | 34 | 4.41 | **0.0056** | 0.2642 | 2.1440 | 0.3140 | n.a. | **0.0031** | 0.3595 | 0.8733 | 0.1186 |
|  | avD_F3 | Aβ42 | morning | -0.5940 | **0.0001** | Aβ42 | 37 | 5 | 31 | 14.8 | **<0.0001** | 0.6571 | 2.6433 | 0.4348 | 0.0831 | **<0.0001** | 0.6274 | 0.7489 | 0.8764 |
|  | avD_F3 | Aβ40 | morning | -0.5672 | **0.0003** | Aβ40 | 37 | 5 | 31 | 10.12 | **<0.0001** | 0.5588 | 187.7136 | **0.0473** | 0.2034 | **0.0003** | 0.6027 | 0.9971 | 0.0980 |
| **F4** |  |  |  |  |  |  |  |  |  |  |  |  |  |  |  |  |  |  |  |
| length | avL_F4 | Aβ42 | evening | -0.5682 | **0.0001** | avL_F4 | 40 | 4 | 35 | 5.65 | **0.0013** | 0.3231 | 0.9020 | **<0.0001** | n.a. | **0.0002** | 0.3371 | 0.5734 | 0.0699 |
|  | avL_F4 | Aβ40 | evening | -0.4294 | **0.0057** | avL_F4 | 40 | 4 | 35 | 2.59 | 0.0532 | 0.1405 | 0.8086 | **<0.0001** | n.a. | **0.0195** | 0.5105 | 0.7357 | 0.1899 |
|  | avL_F4 | Aβ42 | morning | -0.5852 | **0.0001** | Aβ42 | 38 | 5 | 32 | 14.06 | **<0.0001** | 0.6383 | 5.1637 | 0.2987 | 0.2575 | **<0.0001** | 0.7468 | 0.5282 | 0.6483 |
|  | avL_F4 | Aβ40 | morning | -0.4362 | **0.0062** | Aβ40 | 38 | 5 | 32 | 9.34 | **<0.0001** | 0.5298 | 229.5718 | 0.0683 | 0.4380 | **<0.0001** | 0.4586 | 0.9260 | 0.0924 |
| amplitude | avAF4 | Aβ42 | evening | -0.3180 | **0.0455** | avAF4 | 40 | 4 | 35 | 7.41 | **0.0002** | 0.3966 | 11.5812 | **0.0143** | n.a. | **0.0132** | **0.0001** | 0.2167 | 0.2047 |
| density | avD_F4 | Aβ42 | evening | -0.5458 | **0.0003** | avD_F4 | 40 | 4 | 35 | 5.18 | **0.0022** | 0.3003 | 4.1785 | **0.0435** | n.a. | **0.0004** | 0.1057 | 0.4395 | 0.3185 |
|  | avD_F4 | Aβ40 | evening | -0.4858 | **0.0015** | avD_F4 | 40 | 4 | 35 | 3.35 | **0.0201** | 0.1943 | 2.8099 | 0.1960 | n.a. | **0.0049** | 0.1735 | 0.6552 | 0.5863 |
|  | avD_F4 | Aβ42 | morning | -0.5914 | **<0.0001** | Aβ42 | 38 | 5 | 32 | 14.72 | **<0.0001** | 0.6496 | 2.7629 | 0.4226 | 0.1308 | **<0.0001** | 0.5720 | 0.6039 | 0.5819 |
|  | avD_F4 | Aβ40 | morning | -0.4931 | **0.0017** | Aβ40 | 38 | 5 | 32 | 9.78 | **<0.0001** | 0.5426 | 191.5269 | **0.0458** | 0.2255 | **0.0002** | 0.5886 | 0.9400 | 0.0752 |
| **P3** |  |  |  |  |  |  |  |  |  |  |  |  |  |  |  |  |  |  |  |
| length | avL_P3 | Aβ40 | evening | -0.3950 | **0.0128** | avL_P3 | 37 | 4 | 32 | 1.94 | 0.1270 | 0.0950 | 0.9047 | **<0.0001** | n.a. | 0.9848 | 0.6463 | **0.0250** | 0.3203 |
| density | avD_P3 | Aβ40 | evening | -0.3300 | **0.0376** | avD_P3 | 40 | 4 | 35 | 1.86 | 0.1399 | 0.0809 | 3.1088 | 0.1859 | n.a. | 0.2188 | 0.2381 | 0.3648 | 0.6822 |
| **P4** |  |  |  |  |  |  |  |  |  |  |  |  |  |  |  |  |  |  |  |
| length | avL_P4 | Aβ40 | evening | -0.4026 | **0.0100** | avL_P4 | 40 | 4 | 35 | 3.16 | **0.0257** | 0.1811 | 0.8538 | **<0.0001** | n.a. | 0.2433 | 0.6033 | 0.0781 | 0.1946 |
| frequency | avF_P4 | Aβ42 | evening | 0.3121 | **0.0499** | avF_P4 | 40 | 4 | 35 | 0.95 | 0.4476 | -0.0053 | 13.7905 | **<0.0001** | n.a. | 0.0630 | 0.9807 | 0.9181 | 0.9474 |
|  | avF_P4 | Aβ42/40 | evening | 0.3139 | **0.0485** | avF_P4 | 40 | 4 | 35 | 1.43 | 0.2433 | 0.0426 | 13.0807 | **<0.0001** | n.a. | **0.0234** | 0.9916 | 0.2206 | 0.6195 |
|  | avF_P4 | Aβ42 | morning | 0.3695 | **0.0224** | Aβ42 | 38 | 5 | 32 | 14.21 | **<0.0001** | 0.6410 | -13.4902 | 0.2667 | 0.2179 | **<0.0001** | 0.8554 | 0.4525 | 0.3871 |
| density | avD_P4 | Aβ40 | evening | -0.3229 | **0.0422** | avD_P4 | 38 | 4 | 33 | 2.65 | 0.0503 | 0.1517 | 2.8581 | 0.1638 | n.a. | 0.8112 | 0.1688 | **0.0264** | 0.1947 |

**Table S22.** Significant Pearson correlations and adjusted linear regression results for sleep spindle variables in stage N3 and AD biomarkers. Regression models were adjusted for evening AD biomarker levels (n.a. for evening biomarker correlations), sex, age, and BMI. Statistically significant results are shown in bold.

| **Spindle parameter Stage N3** | | **AD biomarker** | **Sampling time** | **Pearson r** | **p** | **Dependent variable** | **n** | **DF model** | **DF error** | **F-value** | **model *p*** | **adjusted R-Square** | **Parameter estimate intercept** | **Intercept *p*** | **Parameter estimate spindle parameter *p*** | **Parameter estimate AD-Biomarker (evening) *p*** | **Parameter estimate Sex *p*** | **Parameter estimate Age *p*** | **Parameter estimate BMI *p*** |
| --- | --- | --- | --- | --- | --- | --- | --- | --- | --- | --- | --- | --- | --- | --- | --- | --- | --- | --- | --- |
| **F3** |  |  |  |  |  |  |  |  |  |  |  |  |  |  |  |  |  |  |  |
| length | avL_F3 | Aβ42 | evening | -0.5320 | **0.0006** | avL_F3 | 38 | 4 | 33 | 3.77 | **0.0124** | 0.2304 | 0.9984 | **<0.0001** | n.a. | **0.0015** | 0.7474 | 0.5872 | 0.2794 |
|  | avL_F3 | Aβ40 | evening | -0.4104 | **0.0105** | avL_F3 | 38 | 4 | 33 | 1.99 | 0.1193 | 0.0965 | 0.8663 | **0.0005** | n.a. | **0.0269** | 0.9577 | 0.7835 | 0.3678 |
|  | avL_F3 | Aβ42 | morning | -0.5514 | **0.0005** | Aβ42 | 36 | 5 | 30 | 14.95 | **<0.0001** | 0.6659 | 2.5794 | 0.5360 | 0.3599 | **<0.0001** | 0.9470 | 0.4938 | 0.8684 |
|  | avL_F3 | Aβ40 | morning | -0.4077 | **0.0136** | Aβ40 | 36 | 5 | 30 | 8.86 | **<0.0001** | 0.5290 | 185.7061 | 0.0851 | 0.4356 | **0.0002** | 0.3238 | 0.7548 | 0.0987 |
|  | avL_F3 | p-tau181 | overnight change | 0.3565 | **0.0280** | p-tau181 | 38 | 5 | 32 | 7.13 | **0.0001** | 0.4531 | -68.8918 | **0.0463** | 0.0631 | **<0.0001** | 0.4338 | 0.1593 | 0.3659 |
| density | avD_F3 | Aβ42 | evening | -0.5197 | **0.0007** | avD_F3 | 39 | 4 | 34 | 4.07 | **0.0084** | 0.2441 | 10.2310 | **0.0283** | n.a. | **0.0020** | 0.7795 | 0.1389 | 0.6578 |
|  | avD_F3 | Aβ40 | evening | -0.3938 | **0.0131** | avD_F3 | 39 | 4 | 34 | 1.72 | 0.1687 | 0.0703 | 7.7583 | 0.1258 | n.a. | 0.1036 | 0.9650 | 0.4923 | 0.8059 |
|  | avD_F3 | Aβ42 | morning | -0.5257 | **0.0008** | Aβ42 | 37 | 5 | 31 | 15.09 | **<0.0001** | 0.6618 | 0.8703 | 0.7943 | 0.5203 | **<0.0001** | 0.9570 | 0.5027 | 0.6918 |
|  | avD_F3 | Aβ40 | morning | -0.3955 | **0.0154** | Aβ40 | 37 | 5 | 31 | 9.01 | **<0.0001** | 0.5266 | 160.9879 | 0.0766 | 0.3168 | **<0.0001** | 0.2721 | 0.9522 | 0.1035 |
|  | avD_F3 | p-tau181 | overnight change | 0.3184 | **0.0482** | p-tau181 | 39 | 5 | 33 | 7.45 | **<0.0001** | 0.4592 | -48.8850 | 0.0991 | 0.0639 | **<0.0001** | 0.4444 | 0.0996 | 0.2253 |
| **F4** |  |  |  |  |  |  |  |  |  |  |  |  |  |  |  |  |  |  |  |
| length | avL_F4 | Aβ42 | evening | -0.4616 | **0.0031** | avL_F4 | 39 | 4 | 34 | 3.61 | **0.0148** | 0.2153 | 0.9485 | **0.0002** | n.a. | **0.0055** | 0.4790 | 0.3045 | 0.0980 |
|  | avL_F4 | Aβ40 | evening | -0.3973 | **0.0123** | avL_F4 | 39 | 4 | 34 | 2.18 | 0.0917 | 0.1108 | 0.8264 | **0.0018** | n.a. | 0.0603 | 0.6175 | 0.8593 | 0.1688 |
|  | avL_F4 | Aβ42 | morning | -0.4574 | **0.0044** | Aβ42 | 37 | 5 | 31 | 14.94 | **<0.0001** | 0.6594 | 1.0823 | 0.7789 | 0.6585 | **<0.0001** | 0.9881 | 0.4564 | 0.7658 |
|  | avL_F4 | p-tau181 | overnight change | 0.3418 | **0.0332** | p-tau181 | 39 | 5 | 33 | 7.33 | **0.0001** | 0.4546 | -62.8421 | 0.0568 | 0.7570 | **<0.0001** | 0.5203 | 0.1325 | 0.4055 |
| amplitude | avA_F4 | Aβ42 | morning | -0.3371 | **0.0414** | Aβ42 | 37 | 5 | 31 | 15.20 | **<0.0001** | 0.6635 | 0.8549 | 0.7931 | 0.4505 | **<0.0001** | 0.7112 | 0.4474 | 0.7988 |
| density | avD_F4 | Aβ42 | evening | -0.5358 | **0.0004** | avD_F4 | 39 | 4 | 34 | 4.66 | **0.0041** | 0.2784 | 7.3243 | 0.0666 | n.a. | **0.0011** | 0.5323 | 0.1641 | 0.2815 |
|  | avD_F4 | Aβ40 | evening | -0.4285 | **0.0065** | avD_F4 | 39 | 4 | 34 | 2.17 | 0.0930 | 0.1099 | 4.9600 | 0.2551 | n.a. | 0.0552 | 0.7230 | 0.6361 | 0.4320 |
|  | avD_F4 | Aβ42 | morning | -0.5488 | **0.0004** | Aβ42 | 37 | 5 | 31 | 15.15 | **<0.0001** | 0.6627 | 0.7926 | 0.8078 | 0.4797 | **<0.0001** | 0.9887 | 0.4996 | 0.7605 |
|  | avD_F4 | Aβ40 | morning | -0.3971 | **0.0150** | Aβ40 | 37 | 5 | 31 | 8.71 | **<0.0001** | 0.5171 | 148.3315 | 0.1005 | 0.5288 | **0.0001** | 0.2905 | 0.8903 | 0.1171 |
| **P4** |  |  |  |  |  |  |  |  |  |  |  |  |  |  |  |  |  |  |  |
| density | avD_P4 | t-tau | morning | 0.3661 | **0.0259** | t-tau | 37 | 5 | 31 | 4.70 | **0.0026** | 0.3392 | 2.8425 | 0.1793 | **0.0383** | **0.0013** | 0.2297 | 0.7464 | 0.2970 |
| length | avL_P4 | t-tau | overnight change | 0.3426 | **0.0379** | t-tau | 37 | 5 | 31 | 8.60 | **<0.0001** | 0.5136 | -2.0112 | 0.5388 | 0.0541 | **<0.0001** | 0.1495 | 0.9541 | 0.3516 |

**Table S23** Significant Pearson correlations and adjusted linear regression results for sleep spindle variables in stage NREM and AD biomarkers. Regression models were adjusted for evening AD biomarker levels (n.a. for evening biomarker correlations), sex, age, and BMI. Statistically significant results are shown in bold.

| **Spindle parameter NREM** | | **AD biomarker** | **Sampling time** | **Pearson r** | **p** | **Depndent variable** | **n** | **DF model** | **DF error** | **F-value** | **model *p*** | **adjusted R-Square** | **Parameter estimate intercept** | **Intercept *p*** | **Parameter estimate spindle parameter *p*** | **Parameter estimate AD-Biomarker (evening) *p*** | **Parameter estimate Sex *p*** | **Parameter estimate Age *p*** | **Parameter estimate BMI *p*** |
| --- | --- | --- | --- | --- | --- | --- | --- | --- | --- | --- | --- | --- | --- | --- | --- | --- | --- | --- | --- |
| **F3** |  |  |  |  |  |  |  |  |  |  |  |  |  |  |  |  |  |  |  |
| length | avL_F3 | Aβ42 | evening | -0.5731 | **0.0001** | avL_F3 | 39 | 4 | 34 | 5.43 | **0.0017** | 0.3180 | 0.8927 | **<0.0001** | n.a. | **0.0003** | 0.6715 | 0.7375 | 0.0761 |
|  | avL_F3 | Aβ40 | evening | -0.4477 | **0.0043** | avL_F3 | 39 | 4 | 34 | 3.01 | **0.0315** | 0.1746 | 0.7986 | **<0.0001** | n.a. | **0.0094** | 0.8776 | 0.5648 | 0.1641 |
|  | avL_F3 | Aβ42 | morning | -0.6197 | **<0.0001** | Aβ42 | 37 | 5 | 31 | 14.41 | **<0.0001** | 0.6507 | 6.9676 | 0.1689 | 0.1181 | **<0.0001** | 0.7672 | 0.5730 | 0.7949 |
|  | avL_F3 | Aβ40 | morning | -0.5123 | **0.0012** | Aβ40 | 37 | 5 | 31 | 10.08 | **<0.0001** | 0.5577 | 272.6547 | **0.0335** | 0.2141 | **0.0001** | 0.5030 | 0.9411 | 0.0856 |
|  | avL_F3 | p-tau181 | overnight change | 0.3205 | **0.0467** | p-tau181 | 39 | 5 | 33 | 5.76 | **0.0006** | 0.3853 | -78.5999 | 0.0510 | 0.1338 | **<0.0001** | 0.5822 | 0.2429 | 0.2478 |
| density | avD_F3 | Aβ42 | evening | -0.5480 | **0.0003** | avD_F3 | 40 | 4 | 35 | 5.01 | **0.0027** | 0.2915 | 4.4853 | **0.0485** | n.a. | **0.0004** | 0.1495 | 0.4145 | 0.3888 |
|  | avD_F3 | Aβ40 | evening | -0.4396 | **0.0045** | avD_F3 | 40 | 4 | 35 | 2.51 | 0.0594 | 0.1340 | 3.0559 | 0.2140 | n.a. | **0.0170** | 0.2575 | 0.8856 | 0.6437 |
|  | avD_F3 | Aβ42 | morning | -0.5774 | **0.0001** | Aβ42 | 38 | 5 | 32 | 14.33 | **<0.0001** | 0.6431 | 2.4772 | 0.4743 | 0.1922 | **<0.0001** | 0.6432 | 0.5875 | 0.5308 |
|  | avD_F3 | Aβ40 | morning | -0.4946 | **0.0016** | Aβ40 | 38 | 5 | 32 | 10.32 | **<0.0001** | 0.5574 | 197.7001 | **0.0363** | 0.1132 | **0.0001** | 0.5943 | 0.8938 | 0.0736 |
|  | avD_F3 | p-tau181 | overnight change | 0.3285 | **0.0385** | p-tau181 | 40 | 5 | 34 | 6.34 | **0.0003** | 0.4064 | -51.4941 | 0.0953 | 0.8865 | **<0.0001** | 0.7689 | 0.1677 | 0.1502 |
| **F4** |  |  |  |  |  |  |  |  |  |  |  |  |  |  |  |  |  |  |  |
| length | avL_F4 | Aβ42 | evening | -0.5753 | **0.0001** | avL_F4 | 40 | 4 | 35 | 6.51 | **0.0005** | 0.3609 | 0.8986 | **<0.0001** | n.a. | **0.0002** | 0.3359 | 0.4132 | 0.0315 |
|  | avL_F4 | Aβ40 | evening | -0.4636 | **0.0026** | avL_F4 | 40 | 4 | 35 | 3.30 | **0.0215** | 0.1906 | 0.7998 | **<0.0001** | n.a. | **0.0128** | 0.5075 | 0.8251 | 0.1095 |
|  | avL_F4 | Aβ42 | morning | -0.5614 | **0.0002** | Aβ42 | 38 | 5 | 32 | 13.59 | **<0.0001** | 0.6298 | 3.6772 | 0.4572 | 0.4567 | **<0.0001** | 0.7899 | 0.5365 | 0.6037 |
|  | avL_F4 | Aβ40 | morning | -0.4245 | **0.0079** | Aβ40 | 38 | 5 | 32 | 9.09 | **<0.0001** | 0.5222 | 190.8442 | 0.1262 | 0.7590 | **<0.0001** | 0.4346 | 0.9101 | 0.0825 |
| amplitude | avA_F4 | Aβ42 | evening | -0.3130 | **0.0492** | avA_F4 | 40 | 4 | 35 | 6.76 | **0.0004** | 0.3714 | 11.0130 | **0.0192** | n.a. | **0.0153** | **0.0002** | 0.3050 | 0.2278 |
| density | avD_F4 | Aβ42 | evening | -0.5877 | **<0.0001** | avD_F4 | 40 | 4 | 35 | 7.13 | **0.0003** | 0.3860 | 4.3285 | **0.0194** | n.a. | **<0.0001** | 0.0533 | 0.2506 | 0.2641 |
|  | avD_F4 | Aβ40 | evening | -0.4897 | **0.0013** | avD_F4 | 40 | 4 | 35 | 3.58 | **0.0150** | 0.2094 | 3.0039 | 0.1412 | n.a. | **0.0077** | 0.1273 | 0.9570 | 0.5300 |
|  | avD_F4 | Aβ42 | morning | -0.6108 | **<0.0001** | Aβ42 | 38 | 5 | 32 | 14.37 | **<0.0001** | 0.6437 | 2.8318 | 0.4254 | 0.1847 | **<0.0001** | 0.5663 | 0.6487 | 0.5827 |
|  | avD_F4 | Aβ40 | morning | -0.4776 | **0.0024** | Aβ40 | 38 | 5 | 32 | 9.57 | **<0.0001** | 0.5368 | 190.8944 | **0.0493** | 0.3008 | **0.0001** | 0.5841 | 0.8837 | 0.0767 |
| **P3** |  |  |  |  |  |  |  |  |  |  |  |  |  |  |  |  |  |  |  |
| length | avL_P3 | Aβ40 | evening | -0.4017 | **0.0113** | avL_P3 | 39 | 4 | 34 | 2.24 | 0.0851 | 0.1155 | 0.8352 | **<0.0001** | n.a. | 0.1470 | 0.8910 | 0.2276 | 0.4102 |
| frequency | avF_P3 | Aβ42/40 | evening | 0.3288 | **0.0410** | avF_P3 | 39 | 4 | 34 | 1.66 | 0.1824 | 0.0648 | 13.1148 | **<0.0001** | n.a. | **0.0196** | 0.8497 | 0.1861 | 0.4782 |
|  | avF_P3 | Aβ42 | morning | 0.3386 | **0.0404** | Aβ42 | 37 | 5 | 31 | 12.83 | **<0.0001** | 0.6216 | 0.8832 | 0.8642 | 0.9862 | **<0.0001** | 0.8963 | 0.5021 | 0.4179 |
| **P4** |  |  |  |  |  |  |  |  |  |  |  |  |  |  |  |  |  |  |  |
| length | avL_P4 | Aβ40 | evening | -0.4443 | **0.0041** | avL_P4 | 40 | 4 | 35 | 3.85 | **0.0107** | 0.2265 | 0.8465 | **<0.0001** | n.a. | 0.1675 | 0.6980 | 0.0570 | 0.1790 |
| frequency | avF_P4 | Aβ42 | evening | 0.3226 | **0.0423** | avF_P4 | 40 | 4 | 35 | 1.03 | 0.4040 | 0.0034 | 13.7154 | **<0.0001** | n.a. | 0.0533 | 0.8582 | 0.9762 | 0.9298 |
|  | avF_P4 | Aβ42/40 | evening | 0.3338 | **0.0353** | avF_P4 | 40 | 4 | 35 | 1.71 | 0.1705 | 0.0676 | 13.0418 | **<0.0001** | n.a. | **0.0140** | 0.8769 | 0.1630 | 0.7048 |
|  | avF_P4 | Aβ42 | morning | 0.3829 | **0.0177** | Aβ42 | 38 | 5 | 32 | 14.36 | **<0.0001** | 0.6436 | -16.7842 | 0.2237 | 0.1865 | **<0.0001** | 0.8287 | 0.4545 | 0.3621 |

**Table S24**. Significant Pearson correlations and adjusted linear regression results for sleep EEG power (whole night, evening AD biomarkers) and AD biomarkers. Regression models were adjusted for evening AD biomarker levels (n.a. for evening biomarker correlations), sex, age, and BMI. Statistically significant results are shown in bold.

| **AD biomarker** | **Sampling time** | **Power [whole night] Frequency band** | **Loc-ation** | **Pearson r  [AD biomarker x power]** | **p** | **Dependent variable** | **n** | **DF model** | **DF error** | **F-value** | **model *p*** | **adjusted R-Square** | **Parameter estimate intercept** | **Intercept *p*** | **Parameter estimate EEG power parameter *p*** | **Parameter estimate AD-Biomarker (evening) *p*** | **Parameter estimate Sex *p*** | **Parameter estimate Age *p*** | **Parameter estimate BMI *p*** |
| --- | --- | --- | --- | --- | --- | --- | --- | --- | --- | --- | --- | --- | --- | --- | --- | --- | --- | --- | --- |
| p-tau181 | evening | delta | Pz | -0.3340 | **0.0377** | delta_Pz | 39 | 4 | 34 | 6.56 | **0.0005** | 0.3694 | 90.4751 | **0.0331** | n.a. | 0.3869 | **0.0009** | 0.3935 | 0.3594 |
| Aβ42 | evening | alpha | F4 | -0.3349 | **0.0371** | alpha_F4 | 39 | 4 | 34 | 3.79 | **0.0118** | 0.2270 | 6.4977 | 0.1764 | n.a. | **0.0179** | **0.0084** | 0.7151 | 0.9175 |
| Aβ42 | evening | alpha | Fz | -0.3168 | **0.0494** | alpha_Fz | 39 | 4 | 34 | 3.50 | **0.0169** | 0.2084 | 7.6485 | 0.1832 | n.a. | **0.0253** | **0.0103** | 0.7348 | 0.9722 |
| Aβ42 | evening | spindle (wide) | F4 | -0.3250 | **0.0435** | spindle (wide)_F4 | 39 | 4 | 34 | 5.96 | **0.0010** | 0.3428 | 3.6792 | 0.0530 | n.a. | **0.0231** | **0.0020** | 0.0933 | 0.4895 |
| Aβ40 | evening | alpha | F3 | -0.3269 | **0.0422** | alpha_F3 | 39 | 4 | 34 | 3.51 | **0.0167** | 0.2091 | 4.3694 | 0.3607 | n.a. | **0.0329** | **0.0105** | 0.5372 | 0.7250 |
| Aβ40 | evening | alpha | F4 | -0.3397 | **0.0344** | alpha_F4 | 39 | 4 | 34 | 3.33 | **0.0211** | 0.1967 | 4.3984 | 0.3635 | n.a. | **0.0377** | **0.0129** | 0.6683 | 0.9260 |
| Aβ40 | evening | alpha | Fp1 | -0.3323 | **0.0388** | alpha_Fp1 | 39 | 4 | 34 | 3.09 | **0.0285** | 0.1802 | 3.0821 | 0.3980 | n.a. | **0.0301** | **0.0214** | 0.4938 | 0.6962 |
| Aβ40 | evening | alpha | Fp2 | -0.3481 | **0.0299** | alpha_Fp2 | 39 | 4 | 34 | 3.15 | **0.0262** | 0.1848 | 3.2382 | 0.4077 | n.a. | **0.0234** | **0.0237** | 0.4766 | 0.7042 |
| Aβ40 | evening | alpha | Fz | -0.3398 | **0.0343** | alpha_F4 | 39 | 4 | 34 | 3.31 | **0.0216** | 0.1955 | 5.2306 | 0.3610 | n.a. | **0.0347** | **0.0140** | 0.6254 | 0.8659 |
| Aβ40 | evening | beta1 | F3 | -0.3658 | **0.0220** | beta1_F3 | 39 | 4 | 34 | 4.18 | **0.0074** | 0.2508 | 2.4318 | 0.1805 | n.a. | 0.0667 | **0.0077** | 0.6578 | 0.8641 |
| Aβ40 | evening | beta1 | F4 | -0.3900 | **0.0141** | beta1_F4 | 39 | 4 | 34 | 5.11 | **0.0025** | 0.3022 | 3.0809 | 0.0774 | n.a. | 0.0748 | **0.0058** | 0.3400 | 0.8232 |
| Aβ40 | evening | beta1 | Fp1 | -0.3627 | **0.0233** | beta1_Fp1 | 39 | 4 | 34 | 3.67 | **0.0138** | 0.2191 | 2.0743 | 0.0704 | n.a. | 0.0754 | **0.0264** | 0.6001 | 0.6911 |
| Aβ40 | evening | beta1 | Fp2 | -0.3896 | **0.0142** | beta1_Fp2 | 39 | 4 | 34 | 3.87 | **0.0107** | 0.2319 | 2.0153 | 0.0915 | n.a. | **0.0464** | **0.0233** | 0.6864 | 0.7809 |
| Aβ40 | evening | beta1 | Fz | -0.3910 | **0.0138** | beta1_Fz | 39 | 4 | 34 | 4.52 | **0.0049** | 0.2704 | 3.0500 | 0.1332 | n.a. | 0.0581 | **0.0083** | 0.5194 | 0.8061 |
| Aβ40 | evening | beta2 | Fp2 | -0.3286 | **0.0411** | beta2_Fp2 | 39 | 4 | 34 | 3.23 | **0.0239** | 0.1898 | 0.2560 | 0.6814 | n.a. | **0.0144** | **0.0191** | 0.2010 | 0.7210 |
| Aβ40 | evening | beta2 | Fz | -0.3175 | **0.0489** | beta2_Fz | 39 | 4 | 34 | 3.78 | **0.0119** | 0.2266 | 0.8486 | 0.3718 | n.a. | **0.0375** | **0.0067** | 0.5682 | 0.7042 |
| Aβ40 | evening | spindle (wide) | F3 | -0.3758 | **0.0184** | spindle (wide)_F3 | 39 | 4 | 34 | 4.58 | **0.0046** | 0.2737 | 2.2910 | 0.2447 | n.a. | 0.0507 | **0.0041** | 0.7359 | 0.6747 |
| Aβ40 | evening | spindle (wide) | F4 | -0.4002 | **0.0116** | spindle (wide)_F4 | 39 | 4 | 34 | 5.36 | **0.0019** | 0.3144 | 2.9043 | 0.1288 | n.a. | 0.0531 | **0.0033** | 0.4433 | 0.6202 |
| Aβ40 | evening | spindle (wide) | Fp1 | -0.3730 | **0.0194** | spindle (wide)_Fp1 | 39 | 4 | 34 | 4.06 | **0.0085** | 0.2437 | 2.0874 | 0.1005 | n.a. | 0.0619 | **0.0130** | 0.6326 | 0.9086 |
| Aβ40 | evening | spindle (wide) | Fp2 | -0.3977 | **0.0122** | spindle (wide)_Fp2 | 39 | 4 | 34 | 4.36 | **0.0059** | 0.2614 | 2.0857 | 0.1186 | n.a. | **0.0378** | **0.0109** | 0.7141 | 0.9558 |
| Aβ40 | evening | spindle (wide) | Fz | -0.4002 | **0.0116** | spindle (wide)_Fz | 39 | 4 | 34 | 4.76 | **0.0037** | 0.2836 | 2.8773 | 0.1899 | n.a. | **0.0454** | **0.0053** | 0.5931 | 0.6044 |
| Aβ40 | evening | spindle (narrow) | F3 | -0.3799 | **0.0170** | spindle (narrow)_F3 | 39 | 4 | 34 | 4.73 | **0.0039** | 0.2817 | 3.0601 | 0.1647 | n.a. | 0.0745 | **0.0052** | 0.4581 | 0.6282 |
| Aβ40 | evening | spindle (narrow) | F4 | -0.4017 | **0.0113** | spindle (narrow)_F4 | 39 | 4 | 34 | 5.65 | **0.0013** | 0.3287 | 4.0687 | 0.0640 | n.a. | 0.0947 | **0.0051** | 0.1799 | 0.5777 |
| Aβ40 | evening | spindle (narrow) | F7 | -0.3292 | **0.0407** | spindle (narrow)_F7 | 39 | 4 | 34 | 3.64 | **0.0142** | 0.2177 | 2.0691 | 0.1232 | n.a. | 0.1007 | **0.0146** | 0.6782 | 0.7546 |
| Aβ40 | evening | spindle (narrow) | Fp1 | -0.3812 | **0.0167** | spindle (narrow)_Fp1 | 39 | 4 | 34 | 3.85 | **0.0110** | 0.2307 | 2.7729 | 0.0527 | n.a. | 0.0963 | **0.0321** | 0.3459 | 0.9159 |
| Aβ40 | evening | spindle (narrow) | Fp2 | -0.4047 | **0.0106** | spindle (narrow)_Fp2 | 39 | 4 | 34 | 4.38 | **0.0058** | 0.2623 | 2.9678 | **0.0461** | n.a. | 0.0689 | **0.0239** | 0.3292 | 0.9292 |
| Aβ40 | evening | spindle (narrow) | Fz | -0.4007 | **0.0115** | spindle (narrow)_Fz | 39 | 4 | 34 | 4.95 | **0.0030** | 0.2938 | 3.7317 | 0.1296 | n.a. | 0.0684 | **0.0063** | 0.3566 | 0.5618 |

**Table S25**. Significant Pearson correlations and adjusted linear regression results for sleep EEG power (whole night, morning AD biomarkers) and AD biomarkers. Regression models were adjusted for evening AD biomarker levels (n.a. for evening biomarker correlations), sex, age, and BMI. Statistically significant results are shown in bold.

| **AD biomarker** | **Sampling time** | **Power [whole night] Frequency band** | **loc-ation** | **Pearson r  [AD biomarker x power]** | **p** | **Dependent variable** | **n** | **DF model** | **DF error** | **F-value** | **model *p*** | **adjusted R-Square** | **Parameter estimate intercept** | **Intercept *p*** | **Parameter estimate EEG power parameter *p*** | **Parameter estimate AD-Biomarker (evening) *p*** | **Parameter estimate Sex *p*** | **Parameter estimate Age *p*** | **Parameter estimate BMI *p*** |
| --- | --- | --- | --- | --- | --- | --- | --- | --- | --- | --- | --- | --- | --- | --- | --- | --- | --- | --- | --- |
| Aβ42/40 | morning | theta | Cz | -0.3627 | **0.0274** | theta_Cz | 37 | 5 | 31 | 12 | **<0.0001** | 0.5937 | -0.0125 | 0.5130 | **0.0482** | **<0.0001** | 0.1485 | 0.9941 | **0.0116** |
| Aβ42/40 | morning | theta | F3 | -0.3493 | **0.0341** | theta_F3 | 37 | 5 | 31 | 11 | **<0.0001** | 0.5852 | -0.0142 | 0.4595 | 0.0705 | **<0.0001** | 0.1806 | 0.8676 | **0.0181** |
| Aβ42/40 | morning | theta | F4 | -0.3469 | **0.0354** | theta_F4 | 37 | 5 | 31 | 11 | **<0.0001** | 0.5829 | -0.0161 | 0.3993 | 0.0780 | **<0.0001** | 0.2187 | 0.8589 | **0.0150** |
| Aβ42/40 | morning | theta | F8 | -0.3271 | **0.0482** | theta_F8 | 37 | 5 | 31 | 11 | **<0.0001** | 0.5849 | -0.0185 | 0.3233 | 0.0714 | **<0.0001** | 0.1943 | 0.7077 | **0.0155** |
| Aβ42/40 | morning | theta | Fz | -0.3517 | **0.0328** | theta_Fz | 37 | 5 | 31 | 11 | **<0.0001** | 0.5860 | -0.0147 | 0.4413 | 0.0682 | **<0.0001** | 0.1773 | 0.9006 | **0.0133** |
| Aβ42/40 | morning | theta | P4 | -0.3267 | **0.0484** | theta_P4 | 37 | 5 | 31 | 10 | **<0.0001** | 0.5672 | -0.0176 | 0.3674 | 0.1596 | **<0.0001** | 0.3053 | 0.7844 | **0.0230** |
| Aβ42/40 | morning | delta | Pz | -0.3692 | **0.0245** | theta_Pz | 37 | 5 | 31 | 9.8 | **<0.0001** | 0.5486 | -0.0172 | 0.4247 | 0.4056 | **<0.0001** | 0.4114 | 0.8386 | **0.0359** |

**Table S26.** Significant Pearson correlations and adjusted linear regression results for sleep EEG power (Stage N1, evening AD biomarkers) and AD biomarkers. Regression models were adjusted for evening AD biomarker levels (n.a. for evening biomarker correlations), sex, age, and BMI. Statistically significant results are shown in bold.

| **AD biomarker** | **Sampling time** | **Power [Stage N1] Frequency band** | **loc-ation** | **Pearson r  [AD biomarker x power]** | **p** | **Dependent variable** | **n** | **DF model** | **DF error** | **F-value** | **model *p*** | **adjusted R-Square** | **Parameter estimate intercept** | **Intercept *p*** | **Parameter estimate EEG power parameter *p*** | **Parameter estimate AD-Biomarker (evening) *p*** | **Parameter estimate Sex *p*** | **Parameter estimate Age *p*** | **Parameter estimate BMI *p*** |
| --- | --- | --- | --- | --- | --- | --- | --- | --- | --- | --- | --- | --- | --- | --- | --- | --- | --- | --- | --- |
| p-tau181 | evening | delta | F7 | -0.3169 | **0.0493** | delta_F7 | 39 | 4 | 34 | 4.14 | **0.0077** | 0.2484 | 33.7963 | **0.0228** | n.a. | 0.2912 | **0.0189** | 0.8151 | 0.1740 |
| Aβ42/40 | evening | theta | O2 | -0.3422 | **0.0330** | theta_O2 | 39 | 4 | 34 | 6.21 | **0.0007** | 0.3542 | 9.9958 | 0.1071 | n.a. | **0.0073** | **0.0008** | 0.9527 | 0.5273 |
| Aβ40 | evening | beta1 | F3 | -0.3427 | **0.0327** | beta1_F3 | 39 | 4 | 34 | 4.18 | **0.0074** | 0.2505 | 1.5228 | 0.3963 | n.a. | **0.0415** | **0.0036** | 0.8137 | 0.8515 |
| Aβ40 | evening | beta1 | F4 | -0.3745 | **0.0188** | beta1_F4 | 39 | 4 | 34 | 5.43 | **0.0017** | 0.3181 | 1.9454 | 0.2135 | n.a. | **0.0362** | **0.0014** | 0.8571 | 0.7730 |
| Aβ40 | evening | beta1 | Fp1 | -0.3586 | **0.0250** | beta1_Fp1 | 39 | 4 | 34 | 4.72 | **0.0039** | 0.2815 | 1.7249 | 0.1043 | n.a. | **0.0382** | **0.0049** | 0.9642 | 0.6359 |
| Aβ40 | evening | beta1 | Fp2 | -0.3998 | **0.0117** | beta1_Fp2 | 39 | 4 | 34 | 5.05 | **0.0027** | 0.2988 | 1.5578 | 0.1417 | n.a. | **0.0174** | **0.0042** | 0.9192 | 0.7966 |
| Aβ40 | evening | beta1 | Fz | -0.3690 | **0.0208** | beta1_Fz | 39 | 4 | 34 | 4.81 | **0.0035** | 0.2860 | 1.9450 | 0.2912 | n.a. | **0.0305** | **0.0024** | 0.9141 | 0.8484 |
| Aβ40 | evening | beta2 | Fp2 | -0.3399 | **0.0342** | beta2_Fp2 | 39 | 4 | 34 | 4.32 | **0.0062** | 0.2588 | 0.3936 | 0.4858 | n.a. | **0.0202** | **0.0030** | 0.4217 | 0.9758 |
| Aβ40 | evening | spindle (wide) | F3 | -0.3264 | **0.0426** | spindle (wide)_F3 | 39 | 4 | 34 | 3.98 | **0.0094** | 0.2385 | 1.3801 | 0.4841 | n.a. | **0.0400** | **0.0036** | 0.6405 | 0.8873 |
| Aβ40 | evening | spindle (wide) | F4 | -0.3610 | **0.0239** | spindle (wide)_F4 | 39 | 4 | 34 | 5.03 | **0.0027** | 0.2980 | 1.8617 | 0.2834 | n.a. | **0.0313** | **0.0016** | 0.8873 | 0.8521 |
| Aβ40 | evening | spindle (wide) | Fp1 | -0.3416 | **0.0333** | spindle (wide)_Fp1 | 39 | 4 | 34 | 4.64 | **0.0043** | 0.2768 | 1.6814 | 0.1574 | n.a. | **0.0347** | **0.0038** | 0.8099 | 0.6244 |
| Aβ40 | evening | spindle (wide) | Fp2 | -0.3845 | **0.0157** | spindle (wide)_Fp2 | 39 | 4 | 34 | 5.05 | **0.0027** | 0.2987 | 1.5413 | 0.1969 | n.a. | **0.0145** | **0.0032** | 0.6834 | 0.7334 |
| Aβ40 | evening | spindle (wide) | Fz | -0.3575 | **0.0255** | spindle (wide)_Fz | 39 | 4 | 34 | 4.59 | **0.0045** | 0.2740 | 1.9497 | 0.3272 | n.a. | **0.0300** | **0.0026** | 0.7824 | 0.8881 |
| Aβ40 | evening | spindle (narrow) | F3 | -0.3372 | **0.0358** | spindle (narrow)_F3 | 39 | 4 | 34 | 3.98 | **0.0093** | 0.2390 | 1.6928 | 0.4265 | n.a. | **0.0451** | **0.0041** | 0.7960 | 0.8105 |
| Aβ40 | evening | spindle (narrow) | F4 | -0.3742 | **0.0189** | spindle (narrow)_F4 | 39 | 4 | 34 | 5.17 | **0.0023** | 0.3050 | 2.3148 | 0.2219 | n.a. | **0.0366** | **0.0018** | 0.8834 | 0.7744 |
| Aβ40 | evening | spindle (narrow) | Fp1 | -0.3706 | **0.0202** | spindle (narrow)_Fp1 | 39 | 4 | 34 | 4.75 | **0.0038** | 0.2828 | 2.1003 | 0.1007 | n.a. | **0.0340** | **0.0054** | 0.9354 | 0.6688 |
| Aβ40 | evening | spindle (narrow) | Fp2 | -0.4023 | **0.0111** | spindle (narrow)_Fp2 | 39 | 4 | 34 | 5.14 | **0.0024** | 0.3035 | 2.0460 | 0.1116 | n.a. | **0.0196** | **0.0044** | 0.9688 | 0.7754 |
| Aβ40 | evening | spindle (narrow) | Fz | -0.3666 | **0.0217** | spindle (narrow)_Fz | 39 | 4 | 34 | 4.60 | **0.0045** | 0.2746 | 2.2145 | 0.2968 | n.a. | **0.0305** | **0.0029** | 0.8696 | 0.8514 |

**Table S27.** Significant Pearson correlations and adjusted linear regression results for sleep EEG power (Stage N1, morning AD biomarkers) and AD biomarkers. Regression models were adjusted for evening AD biomarker levels (n.a. for evening biomarker correlations), sex, age, and BMI. Statistically significant results are shown in bold.

| **AD biomarker** | **Sampling time** | **Power [Stage N1] Frequency band** | **location** | **Pearson r  [AD biomarker x power]** | **p** | **Dependent variable** | **n** | **DF model** | **DF error** | **F-value** | **model *p*** | **adjusted R-Square** | **Parameter estimate intercept** | **Intercept *p*** | **Parameter estimate EEG power parameter *p*** | **Parameter estimate AD-Biomarker (evening) *p*** | **Parameter estimate Sex *p*** | **Parameter estimate Age *p*** | **Parameter estimate BMI *p*** |
| --- | --- | --- | --- | --- | --- | --- | --- | --- | --- | --- | --- | --- | --- | --- | --- | --- | --- | --- | --- |
| p-tau181 | morning | delta | F8 | -0.3226 | **0.0452** | delta_F8 | 39 | 5 | 33 | 2.34 | 0.0635 | 0.1499 | -23.6552 | 0.4543 | 0.0740 | 0.2422 | 0.2172 | 0.3797 | 0.1189 |
| p-tau181 | morning | delta | Fp2 | -0.3261 | **0.0428** | delta_Fp2 | 39 | 5 | 33 | 2.03 | 0.0998 | 0.1195 | -25.1119 | 0.4412 | 0.1521 | 0.4297 | 0.3402 | 0.2311 | 0.2245 |
| Aβ42/40 | morning | delta | Fz | -0.3570 | **0.0301** | delta_Fz | 37 | 5 | 31 | 10.39 | **<0.0001** | 0.5660 | -0.0120 | 0.5662 | 0.1688 | **<0.0001** | 0.2875 | 0.7629 | **0.0321** |
| Aβ42/40 | morning | theta | Cz | -0.3319 | **0.0448** | theta_Cz | 37 | 5 | 31 | 10.97 | **<0.0001** | 0.5808 | -0.0181 | 0.3368 | 0.0861 | **<0.0001** | 0.2785 | 0.7681 | **0.0174** |
| Aβ42/40 | morning | theta | F3 | -0.3349 | **0.0428** | theta_F3 | 37 | 5 | 31 | 10.94 | **<0.0001** | 0.5800 | -0.0191 | 0.3089 | 0.0891 | **<0.0001** | 0.2819 | 0.7011 | **0.0199** |
| Aβ42/40 | morning | theta | F4 | -0.3412 | **0.0388** | theta_F4 | 37 | 5 | 31 | 10.81 | **<0.0001** | 0.5766 | -0.0195 | 0.3010 | 0.1038 | **<0.0001** | 0.3185 | 0.7177 | **0.0184** |
| Aβ42/40 | morning | theta | Fz | -0.3364 | **0.0417** | theta_Fz | 37 | 5 | 31 | 10.84 | **<0.0001** | 0.5776 | -0.0189 | 0.3175 | 0.0994 | **<0.0001** | 0.2820 | 0.7385 | **0.0178** |
| Aβ42/40 | morning | theta | O1 | -0.3761 | **0.0218** | theta_O1 | 37 | 5 | 31 | 9.86 | **<0.0001** | 0.5516 | -0.0208 | 0.2919 | 0.3441 | **<0.0001** | 0.3893 | 0.5969 | **0.0359** |
| Aβ42/40 | morning | theta | O2 | -0.3803 | **0.0202** | theta_O2 | 37 | 5 | 31 | 9.62 | **<0.0001** | 0.5449 | -0.0218 | 0.2759 | 0.5078 | **<0.0001** | 0.4821 | 0.6115 | **0.0340** |
| Aβ42/40 | morning | theta | Pz | -0.3689 | **0.0247** | theta_Pz | 37 | 5 | 31 | 10.73 | **<0.0001** | 0.5747 | -0.0187 | 0.3242 | 0.1131 | **<0.0001** | 0.3592 | 0.7001 | **0.0235** |

**Table S28.** Significant Pearson correlations and adjusted linear regression results for sleep EEG power (Stage N2, evening AD biomarkers) and AD biomarkers. Regression models were adjusted for evening AD biomarker levels (n.a. for evening biomarker correlations), sex, age, and BMI. Statistically significant results are shown in bold.

| **AD biomarker** | **Sampling time** | **Power [Stage N2] Frequency band** | **location** | **Pearson r  [AD biomarker x power]** | **p** | **Dependent variable** | **n** | **DF model** | **DF error** | **F-value** | **model *p*** | **adjusted R-Square** | **Parameter estimate intercept** | **Intercept *p*** | **Parameter estimate EEG power parameter *p*** | **Parameter estimate AD-Biomarker (evening) *p*** | **Parameter estimate Sex *p*** | **Parameter estimate Age *p*** | **Parameter estimate BMI *p*** |
| --- | --- | --- | --- | --- | --- | --- | --- | --- | --- | --- | --- | --- | --- | --- | --- | --- | --- | --- | --- |
| Aβ42 | evening | alpha | F3 | -0.3207 | **0.0466** | alpha_F3 | 39 | 4 | 34 | 4.90 | **0.0031** | 0.2912 | 4.9927 | 0.1770 | n.a. | **0.0130** | **0.0015** | 0.9795 | 0.9691 |
| Aβ42 | evening | alpha | F4 | -0.3731 | **0.0193** | alpha_F4 | 39 | 4 | 34 | 5.37 | **0.0018** | 0.3149 | 6.0919 | 0.1098 | n.a. | **0.0047** | **0.0020** | 0.9118 | 0.8842 |
| Aβ42 | evening | alpha | Fz | -0.3702 | **0.0203** | alpha_Fz | 39 | 4 | 34 | 4.89 | **0.0032** | 0.2907 | 6.8744 | 0.1062 | n.a. | **0.0063** | **0.0033** | 0.8269 | 0.9651 |
| Aβ42 | evening | spindle (wide) | F3 | -0.3222 | **0.0455** | spindle (wide)_F3 | 39 | 4 | 34 | 6.26 | **0.0007** | 0.3565 | 3.4047 | 0.1157 | n.a. | **0.0230** | **0.0011** | 0.0990 | 0.2455 |
| Aβ42 | evening | spindle (wide) | F4 | -0.3634 | **0.0230** | spindle (wide)_F4 | 39 | 4 | 34 | 8.21 | **<0.0001** | 0.4314 | 4.5255 | **0.0311** | n.a. | **0.0093** | **0.0007** | **0.0245** | 0.2359 |
| Aβ42 | evening | spindle (wide) | Fz | -0.3481 | **0.0299** | spindle (wide)_Fz | 39 | 4 | 34 | 6.50 | **0.0005** | 0.3667 | 4.4923 | 0.0608 | n.a. | **0.0174** | **0.0023** | **0.0468** | 0.2307 |
| Aβ42 | evening | spindle (narrow) | F4 | -0.3224 | **0.0453** | spindle (narrow)_F4 | 39 | 4 | 34 | 8.68 | **<0.0001** | 0.4471 | 6.7134 | **0.0130** | n.a. | **0.0286** | **0.0016** | **0.0032** | 0.2084 |
| Aβ42 | evening | spindle (narrow) | Fz | -0.3194 | **0.0475** | spindle (narrow)_Fz | 39 | 4 | 34 | 7.18 | **0.0003** | 0.3941 | 6.2801 | **0.0319** | n.a. | **0.0344** | **0.0028** | **0.0101** | 0.1946 |
| Aβ40 | evening | alpha | Fz | -0.3164 | **0.0497** | alpha_Fz | 39 | 4 | 34 | 3.56 | **0.0157** | 0.2125 | 4.8295 | 0.2741 | n.a. | **0.0468** | **0.0077** | 0.6540 | 0.8339 |
| Aβ40 | evening | beta1 | C3 | -0.3291 | **0.0408** | beta1_C3 | 39 | 4 | 34 | 4.29 | **0.0064** | 0.2573 | 2.4839 | 0.3953 | n.a. | 0.1146 | **0.0032** | 0.6264 | 0.4921 |
| Aβ40 | evening | beta1 | C4 | -0.3514 | **0.0283** | beta1_C4 | 39 | 4 | 34 | 5.63 | **0.0014** | 0.3274 | 3.1101 | 0.2356 | n.a. | 0.1228 | **0.0013** | 0.3343 | 0.3946 |
| Aβ40 | evening | beta1 | Cz | -0.3382 | **0.0352** | beta1_Cz | 39 | 4 | 34 | 4.89 | **0.0032** | 0.2904 | 3.0554 | 0.3811 | n.a. | 0.1617 | **0.0024** | 0.3463 | 0.2149 |
| Aβ40 | evening | beta1 | F3 | -0.4294 | **0.0064** | beta1_F3 | 39 | 4 | 34 | 6.25 | **0.0007** | 0.3559 | 2.8763 | 0.1316 | n.a. | **0.0435** | **0.0023** | 0.2986 | 0.4194 |
| Aβ40 | evening | beta1 | F4 | -0.4406 | **0.0050** | beta1_F4 | 39 | 4 | 34 | 7.53 | **0.0002** | 0.4072 | 4.0122 | **0.0349** | n.a. | 0.0718 | **0.0024** | 0.0723 | 0.4047 |
| Aβ40 | evening | beta1 | F7 | -0.3604 | **0.0242** | beta1_F7 | 39 | 4 | 34 | 4.72 | **0.0039** | 0.2811 | 1.7883 | 0.0908 | n.a. | 0.0714 | **0.0060** | 0.5631 | 0.8155 |
| Aβ40 | evening | beta1 | F8 | -0.3170 | **0.0493** | beta1_F8 | 39 | 4 | 34 | 4.97 | **0.0029** | 0.2947 | 1.6962 | 0.1162 | n.a. | 0.2637 | **0.0033** | 0.1851 | 0.5066 |
| Aβ40 | evening | beta1 | Fp1 | -0.4125 | **0.0091** | beta1_Fp1 | 39 | 4 | 34 | 4.92 | **0.0031** | 0.2924 | 2.3121 | **0.0371** | n.a. | 0.0808 | **0.0167** | 0.2081 | 0.8711 |
| Aβ40 | evening | beta1 | Fp2 | -0.4324 | **0.0060** | beta1_Fp2 | 39 | 4 | 34 | 5.75 | **0.0012** | 0.3333 | 2.6744 | **0.0223** | n.a. | 0.0626 | **0.0120** | 0.1601 | 0.9466 |
| Aβ40 | evening | beta1 | Fz | -0.4525 | **0.0038** | beta1_Fz | 39 | 4 | 34 | 6.52 | **0.0005** | 0.3676 | 3.7992 | 0.0776 | n.a. | **0.0439** | **0.0041** | 0.1748 | 0.3852 |
| Aβ40 | evening | beta2 | Fp2 | -0.3277 | **0.0417** | beta2_Fp2 | 39 | 4 | 34 | 4.72 | **0.0039** | 0.2812 | 0.5015 | 0.1439 | n.a. | **0.0294** | **0.0035** | 0.6220 | 0.4779 |
| Aβ40 | evening | beta2 | Fz | -0.3228 | **0.0450** | beta2_Fz | 39 | 4 | 34 | 5.60 | **0.0014** | 0.3264 | 0.4046 | 0.4618 | n.a. | **0.0363** | **0.0004** | 0.6889 | 0.7777 |

Table S28 (continued)

| **AD biomarker** | **Sampling time** | **Power [Stage N2] Frequency band** | **Loc-ation** | **Pearson r  [AD biomarker x power]** | **p** | **Dependent variable** | **n** | **DF model** | **DF error** | **F-value** | **model *p*** | **adjusted R-Square** | **Parameter estimate intercept** | **Intercept *p*** | **Parameter estimate EEG power parameter *p*** | **Parameter estimate AD-Biomarker (evening) *p*** | **Parameter estimate Sex *p*** | **Parameter estimate Age *p*** | **Parameter estimate BMI *p*** |
| --- | --- | --- | --- | --- | --- | --- | --- | --- | --- | --- | --- | --- | --- | --- | --- | --- | --- | --- | --- |
| Aβ40 | evening | spindle (wide) | C3 | -0.3290 | **0.0408** | spindle (wide)_C3 | 39 | 4 | 34 | 4.43 | **0.0055** | 0.2652 | 2.4499 | 0.4019 | n.a. | 0.1027 | **0.0024** | 0.6905 | 0.4972 |
| Aβ40 | evening | spindle (wide) | C4 | -0.3531 | **0.0274** | spindle (wide)_C4 | 39 | 4 | 34 | 5.65 | **0.0013** | 0.3286 | 3.0123 | 0.2625 | n.a. | 0.0949 | **0.0010** | 0.4545 | 0.4298 |
| Aβ40 | evening | spindle (wide) | Cz | -0.3338 | **0.0379** | spindle (wide)_Cz | 39 | 4 | 34 | 4.66 | **0.0042** | 0.2781 | 2.9661 | 0.3923 | n.a. | 0.1489 | **0.0025** | 0.4312 | 0.2465 |
| Aβ40 | evening | spindle (wide) | F3 | -0.4236 | **0.0072** | spindle (wide)_F3 | 39 | 4 | 34 | 6.04 | **0.0009** | 0.3465 | 2.4809 | 0.2491 | n.a. | **0.0310** | **0.0016** | 0.5254 | 0.3471 |
| Aβ40 | evening | spindle (wide) | F4 | -0.4414 | **0.0049** | spindle (wide)_F4 | 39 | 4 | 34 | 6.96 | **0.0003** | 0.3854 | 3.5646 | 0.0961 | n.a. | **0.0416** | **0.0016** | 0.2152 | 0.3390 |
| Aβ40 | evening | spindle (wide) | F7 | -0.3499 | **0.0290** | spindle (wide)_F7 | 39 | 4 | 34 | 4.48 | **0.0051** | 0.2683 | 1.7181 | 0.1836 | n.a. | 0.0620 | **0.0044** | 0.7845 | 0.9590 |
| Aβ40 | evening | spindle (wide) | F8 | -0.3190 | **0.0478** | spindle (wide)_F8 | 39 | 4 | 34 | 5.03 | **0.0027** | 0.2981 | 1.3063 | 0.3038 | n.a. | 0.1636 | **0.0014** | 0.4259 | 0.3558 |
| Aβ40 | evening | spindle (wide) | Fp1 | -0.4126 | **0.0090** | spindle (wide)_Fp1 | 39 | 4 | 34 | 4.99 | **0.0028** | 0.2960 | 2.2381 | 0.0832 | n.a. | 0.0540 | **0.0080** | 0.3740 | 0.7315 |
| Aβ40 | evening | spindle (wide) | Fp2 | -0.4293 | **0.0064** | spindle (wide)_Fp2 | 39 | 4 | 34 | 5.67 | **0.0013** | 0.3297 | 2.6671 | 0.0547 | n.a. | **0.0403** | **0.0059** | 0.3341 | 0.8631 |
| Aβ40 | evening | spindle (wide) | Fz | -0.4475 | **0.0043** | spindle (wide)_Fz | 39 | 4 | 34 | 6.02 | **0.0009** | 0.3456 | 3.4459 | 0.1507 | n.a. | **0.0327** | **0.0036** | 0.3394 | 0.3301 |
| Aβ40 | evening | spindle (narrow) | C3 | -0.3207 | **0.0465** | spindle (narrow)_C3 | 39 | 4 | 34 | 5.00 | **0.0028** | 0.2965 | 2.6862 | 0.4284 | n.a. | 0.1458 | **0.0013** | 0.4934 | 0.3255 |
| Aβ40 | evening | spindle (narrow) | C4 | -0.3483 | **0.0298** | spindle (narrow)_C4 | 39 | 4 | 34 | 6.15 | **0.0008** | 0.3517 | 3.5504 | 0.2683 | n.a. | 0.1303 | **0.0007** | 0.3022 | 0.3017 |
| Aβ40 | evening | spindle (narrow) | F3 | -0.4361 | **0.0055** | spindle (narrow)_F3 | 39 | 4 | 34 | 6.88 | **0.0004** | 0.3822 | 3.9004 | 0.1290 | n.a. | **0.0492** | **0.0017** | 0.1985 | 0.2783 |
| Aβ40 | evening | spindle (narrow) | F4 | -0.4434 | **0.0047** | spindle (narrow)_F4 | 39 | 4 | 34 | 7.74 | **0.0002** | 0.4151 | 5.7023 | **0.0368** | n.a. | 0.0881 | **0.0028** | **0.0444** | 0.2846 |
| Aβ40 | evening | spindle (narrow) | F7 | -0.3833 | **0.0160** | spindle (narrow)_F7 | 39 | 4 | 34 | 5.27 | **0.0021** | 0.3103 | 2.5289 | 0.0756 | n.a. | 0.0729 | **0.0047** | 0.3665 | 0.9663 |
| Aβ40 | evening | spindle (narrow) | F8 | -0.3305 | **0.0399** | spindle (narrow)_F8 | 39 | 4 | 34 | 5.41 | **0.0018** | 0.3172 | 2.4623 | 0.1126 | n.a. | 0.2939 | **0.0033** | 0.1056 | 0.3467 |
| Aβ40 | evening | spindle (narrow) | Fp1 | -0.4285 | **0.0065** | spindle (narrow)_Fp1 | 39 | 4 | 34 | 5.16 | **0.0023** | 0.3048 | 3.4051 | **0.0316** | n.a. | 0.0977 | **0.0249** | 0.1066 | 0.6609 |
| Aβ40 | evening | spindle (narrow) | Fp2 | -0.4482 | **0.0042** | spindle (narrow)_Fp2 | 39 | 4 | 34 | 6.24 | **0.0007** | 0.3553 | 3.9891 | **0.0163** | n.a. | 0.0780 | **0.0159** | 0.0689 | 0.7292 |
| Aβ40 | evening | spindle (narrow) | Fz | -0.4551 | **0.0036** | spindle (narrow)_Fz | 39 | 4 | 34 | 6.84 | **0.0004** | 0.3806 | 5.1568 | 0.0767 | n.a. | 0.0532 | **0.0040** | 0.1163 | 0.2729 |

**Table S29.** Significant Pearson correlations and adjusted linear regression results for sleep EEG power (Stage N2, morning AD biomarkers) and AD biomarkers. Regression models were adjusted for evening AD biomarker levels (n.a. for evening biomarker correlations), sex, age, and BMI. Statistically significant results are shown in bold.

| **AD biomarker** | **Sampling time** | **Power [Stage N2] Frequency band** | **location** | **Pearson r  [AD biomarker x power]** | **p** | **Dependent variable** | **n** | **DF model** | **DF error** | **F-value** | **model *p*** | **adjusted R-Square** | **Parameter estimate intercept** | **Intercept *p*** | **Parameter estimate EEG power parameter *p*** | **Parameter estimate AD-Biomarker (evening) *p*** | **Parameter estimate Sex *p*** | **Parameter estimate Age *p*** | **Parameter estimate BMI *p*** |
| --- | --- | --- | --- | --- | --- | --- | --- | --- | --- | --- | --- | --- | --- | --- | --- | --- | --- | --- | --- |
| Aβ42 | morning | alpha | F4 | -0.3461 | **0.0359** | alpha_F4 | 37 | 5 | 31 | 13.04 | **<0.0001** | 0.6258 | 1.5113 | 0.6670 | 0.5590 | **<0.0001** | 0.6844 | 0.4822 | 0.4065 |
| Aβ42 | morning | alpha | Fz | -0.3500 | **0.0337** | alpha_Fz | 37 | 5 | 31 | 13.05 | **<0.0001** | 0.6260 | 1.5383 | 0.6619 | 0.5498 | **<0.0001** | 0.6849 | 0.4944 | 0.4156 |
| Aβ42 | morning | spindle (wide) | F3 | -0.3460 | **0.0359** | spindle (wide)_F3 | 37 | 5 | 31 | 13.19 | **<0.0001** | 0.6287 | 1.6688 | 0.6331 | 0.4468 | **<0.0001** | 0.6087 | 0.6329 | 0.5168 |
| Aβ42 | morning | spindle (wide) | F4 | -0.3650 | **0.0263** | spindle (wide)_F4 | 37 | 5 | 31 | 13.01 | **<0.0001** | 0.6251 | 1.6541 | 0.6486 | 0.5933 | **<0.0001** | 0.6898 | 0.6390 | 0.4894 |
| Aβ42 | morning | spindle (wide) | Fz | -0.3597 | **0.0288** | spindle (wide)_Fz | 37 | 5 | 31 | 13.06 | **<0.0001** | 0.6261 | 1.6353 | 0.6464 | 0.5476 | **<0.0001** | 0.6793 | 0.6344 | 0.5011 |
| Aβ42 | morning | spindle (narrow) | F3 | -0.3381 | **0.0407** | spindle (wide)_F3 | 37 | 5 | 31 | 13.06 | **<0.0001** | 0.6262 | 1.6485 | 0.6439 | 0.5421 | **<0.0001** | 0.6670 | 0.6628 | 0.5077 |
| Aβ42 | morning | spindle (narrow) | F4 | -0.3329 | **0.0441** | spindle (narrow)_F4 | 37 | 5 | 31 | 12.95 | **<0.0001** | 0.6241 | 1.6416 | 0.6591 | 0.6538 | **<0.0001** | 0.7313 | 0.6747 | 0.4837 |
| Aβ42 | morning | spindle (narrow) | Fz | -0.3431 | **0.0376** | spindle (narrow)_Fz | 37 | 5 | 31 | 13.06 | **<0.0001** | 0.6262 | 1.7534 | 0.6286 | 0.5413 | **<0.0001** | 0.6791 | 0.6939 | 0.5105 |
| Aβ42 | morning | spindle (narrow) | Pz | -0.3470 | **0.0354** | spindle (narrow)_Pz | 37 | 5 | 31 | 15.71 | **<0.0001** | 0.6714 | 0.9704 | 0.7591 | **0.0379** | **<0.0001** | 0.2240 | 0.6629 | 0.8649 |
| Aβ40 | morning | spindle (wide) | Fz | -0.3337 | **0.0435** | spindle (wide)_Fz | 37 | 5 | 31 | 9.29 | **<0.0001** | 0.5353 | 161.9963 | 0.0973 | 0.8470 | **<0.0001** | 0.4698 | 0.9491 | **0.0397** |
| Aβ40 | morning | spindle (narrow) | Fz | -0.3450 | **0.0365** | spindle (narrow)_Fz | 37 | 5 | 31 | 9.29 | **<0.0001** | 0.5351 | 162.3098 | 0.1020 | 0.8848 | **<0.0001** | 0.4832 | 0.9454 | **0.0410** |

**Table S30.** Significant Pearson correlations and adjusted linear regression results for sleep EEG power (Stage N2, overnight change of AD biomarkers) and AD biomarkers. Regression models were adjusted for evening AD biomarker levels (n.a. for evening biomarker correlations), sex, age, and BMI. Statistically significant results are shown in bold.

| **AD biomarker** | **Sampling time** | **Power [Stage N2] Frequency band** | **Loc-ation** | **Pearson r  [AD biomarker x power]** | **p** | **Dependent variable** | **n** | **DF model** | **DF error** | **F-value** | **model *p*** | **adjusted R-Square** | **Parameter estimate intercept** | **Intercept *p*** | **Parameter estimate EEG power parameter *p*** | **Parameter estimate AD-Biomarker (evening) *p*** | **Parameter estimate Sex *p*** | **Parameter estimate Age *p*** | **Parameter estimate BMI *p*** |
| --- | --- | --- | --- | --- | --- | --- | --- | --- | --- | --- | --- | --- | --- | --- | --- | --- | --- | --- | --- |
| Aβ42 | overnight change | spindle (narrow) | O1 | 0.3367 | **0.0416** | alpha_O1 | 37 | 5 | 31 | 1.53 | 0.2106 | 0.0680 | -2.8057 | 0.4102 | 0.0965 | 0.0475 | 0.3719 | 0.5928 | 0.2415 |
| Aβ42/40 | overnight change | beta1 | C3 | 0.3286 | **0.0471** | beta1_C3 | 37 | 5 | 31 | 1.40 | 0.2519 | 0.0525 | -164.3099 | 0.0864 | 0.8691 | 0.1941 | 0.4780 | 0.9648 | **0.0393** |
| Aβ42/40 | overnight change | beta1 | F7 | 0.3419 | **0.0383** | beta1_F7 | 37 | 5 | 31 | 1.42 | 0.2450 | 0.0550 | -157.6786 | 0.1092 | 0.7452 | 0.2203 | 0.4316 | 0.9548 | **0.0409** |
| Aβ42/40 | overnight change | beta1 | P3 | 0.3279 | **0.0476** | beta1_P3 | 37 | 5 | 31 | 1.40 | 0.2519 | 0.0525 | -169.3870 | 0.0792 | 0.8692 | 0.1652 | 0.5796 | 0.9912 | **0.0406** |
| Aβ42/40 | overnight change | beta1 | T3 | 0.3911 | **0.0167** | beta1_T3 | 37 | 5 | 31 | 1.42 | 0.2440 | 0.0553 | -161.9514 | 0.0903 | 0.7321 | 0.1975 | 0.4273 | 0.9695 | **0.0383** |
| Aβ42/40 | overnight change | beta1 | T4 | 0.3436 | **0.0373** | beta1_T4 | 37 | 5 | 31 | 1.42 | 0.2449 | 0.0550 | -158.6569 | 0.1041 | 0.7440 | 0.1988 | 0.4311 | 0.9521 | **0.0400** |
| Aβ42/40 | overnight change | beta2 | C3 | 0.3486 | **0.0345** | beta2_C3 | 37 | 5 | 31 | 1.51 | 0.2165 | 0.0657 | -159.5412 | 0.0919 | 0.5010 | 0.2349 | 0.3439 | 0.9971 | **0.0381** |
| Aβ42/40 | overnight change | beta2 | F7 | 0.3342 | **0.0432** | beta2_F7 | 37 | 5 | 31 | 1.43 | 0.2421 | 0.0560 | -160.0423 | 0.0961 | 0.7090 | 0.2193 | 0.4194 | 0.9834 | **0.0483** |
| Aβ42/40 | overnight change | beta2 | P3 | 0.3436 | **0.0374** | beta2_P3 | 37 | 5 | 31 | 1.48 | 0.2246 | 0.0625 | -155.5446 | 0.1050 | 0.5540 | 0.2040 | 0.3620 | 0.9774 | **0.0448** |
| Aβ42/40 | overnight change | beta2 | T3 | 0.4055 | **0.0128** | beta2_T3 | 37 | 5 | 31 | 1.52 | 0.2131 | 0.0670 | -160.7641 | 0.0886 | 0.4808 | 0.2298 | 0.3427 | 0.9327 | **0.0450** |
| Aβ42/40 | overnight change | beta2 | T4 | 0.3724 | **0.0232** | beta2_T4 | 37 | 5 | 31 | 1.56 | 0.2019 | 0.0716 | -154.8602 | 0.1023 | 0.4214 | 0.2617 | 0.3166 | 0.9216 | **0.0489** |
| Aβ42/40 | overnight change | beta2 | T5 | 0.3560 | **0.0306** | beta2_T5 | 37 | 5 | 31 | 1.43 | 0.2403 | 0.0567 | -158.2039 | 0.1020 | 0.6884 | 0.1886 | 0.4126 | 0.9953 | 0.0517 |
| Aβ42/40 | overnight change | spindle (wide) | T3 | 0.3766 | **0.0216** | spindle (wide)_T3 | 37 | 5 | 31 | 1.42 | 0.2452 | 0.0549 | -162.9662 | 0.0874 | 0.7476 | 0.2016 | 0.4323 | 0.9805 | **0.0385** |
| Aβ42/40 | overnight change | spindle (wide) | T4 | 0.3308 | **0.0455** | spindle (wide)_T4 | 37 | 5 | 31 | 1.40 | 0.2503 | 0.0531 | -162.0874 | 0.0951 | 0.8332 | 0.1983 | 0.4632 | 0.9700 | **0.0401** |
| Aβ42/40 | overnight change | spindle (narrow) | F7 | 0.3327 | **0.0442** | spindle (narrow)_F7 | 37 | 5 | 31 | 1.41 | 0.2475 | 0.0541 | -158.5809 | 0.1089 | 0.7820 | 0.2139 | 0.4447 | 0.9455 | **0.0394** |
| Aβ42/40 | overnight change | spindle (narrow) | T3 | 0.3773 | **0.0213** | spindle (narrow)_T3 | 37 | 5 | 31 | 1.40 | 0.2519 | 0.0525 | -164.2295 | 0.0869 | 0.8704 | 0.1863 | 0.4725 | 0.9701 | **0.0391** |
| Aβ42/40 | overnight change | spindle (narrow) | T4 | 0.3283 | **0.0473** | spindle (narrow)_T4 | 37 | 5 | 31 | 1.40 | 0.2528 | 0.0522 | -163.3003 | 0.0966 | 0.8990 | 0.1844 | 0.4839 | 0.9635 | **0.0397** |

**Table S31.** Significant Pearson correlations and adjusted linear regression results for sleep EEG power (Stage N3, evening AD biomarkers) and AD biomarkers. Regression models were adjusted for evening AD biomarker levels (n.a. for evening biomarker correlations), sex, age, and BMI. Statistically significant results are shown in bold.

| **AD biomarker** | **Sampling time** | **Power [Stage N3] Frequency band** | **location** | **Pearson r  [AD biomarker x power]** | **p** | **Dependent variable** | **n** | **DF model** | **DF error** | **F-value** | **model *p*** | **adjusted R-Square** | **Parameter estimate intercept** | **Intercept *p*** | **Parameter estimate EEG power parameter *p*** | **Parameter estimate AD-Biomarker (evening) *p*** | **Parameter estimate Sex *p*** | **Parameter estimate Age *p*** | **Parameter estimate BMI *p*** |
| --- | --- | --- | --- | --- | --- | --- | --- | --- | --- | --- | --- | --- | --- | --- | --- | --- | --- | --- | --- |
| Aβ42 | evening | alpha | F3 | -0.4115 | **0.0103** | alpha_F3 | 38 | 4 | 33 | 5.44 | **0.0018** | 0.3242 | 7.9752 | 0.0921 | n.a. | **0.0047** | **0.0043** | 0.3216 | 0.5765 |
| Aβ42 | evening | alpha | F4 | -0.4274 | **0.0074** | alpha_F4 | 38 | 4 | 33 | 4.51 | **0.0051** | 0.2752 | 6.9144 | 0.2139 | n.a. | **0.0045** | **0.0117** | 0.4941 | 0.4172 |
| Aβ42 | evening | alpha | Fp1 | -0.3653 | **0.0241** | alpha_Fp1 | 38 | 4 | 33 | 4.83 | **0.0035** | 0.2926 | 3.8833 | 0.1489 | n.a. | **0.0129** | **0.0043** | 0.3146 | 0.4475 |
| Aβ42 | evening | alpha | Fp2 | -0.3507 | **0.0309** | alpha_Fp2 | 38 | 4 | 33 | 4.78 | **0.0037** | 0.2903 | 3.6768 | 0.2008 | n.a. | **0.0157** | **0.0033** | 0.3624 | 0.4128 |
| Aβ42 | evening | alpha | Fz | -0.4275 | **0.0074** | alpha_Fz | 38 | 4 | 33 | 4.26 | **0.0068** | 0.2608 | 9.9726 | 0.1183 | n.a. | **0.0062** | **0.0262** | 0.2942 | 0.4327 |
| Aβ42 | evening | beta1 | F4 | -0.3421 | **0.0356** | beta1_F4 | 38 | 4 | 33 | 6.31 | **0.0007** | 0.3646 | 3.5943 | **0.0109** | n.a. | **0.0348** | **0.0177** | **0.0068** | 0.4274 |
| Aβ42 | evening | beta1 | Fz | -0.3385 | **0.0377** | beta1_Fz | 38 | 4 | 33 | 5.77 | **0.0012** | 0.3400 | 3.7872 | **0.0183** | n.a. | **0.0403** | **0.0236** | **0.0094** | 0.3602 |
| Aβ42 | evening | spindle (wide) | Cz | -0.3480 | **0.0323** | spindle (wide)_Cz | 38 | 4 | 33 | 5.76 | **0.0013** | 0.3397 | 3.1233 | 0.2191 | n.a. | **0.0171** | **0.0019** | 0.1594 | 0.1630 |
| Aβ42 | evening | spindle (wide) | F3 | -0.4293 | **0.0072** | spindle (wide)_F3 | 38 | 4 | 33 | 6.51 | **0.0006** | 0.3732 | 3.6692 | 0.0512 | n.a. | **0.0047** | **0.0087** | **0.0450** | 0.2484 |
| Aβ42 | evening | spindle (wide) | F4 | -0.4713 | **0.0028** | spindle (wide)_F4 | 38 | 4 | 33 | 8.01 | **0.0001** | 0.4311 | 3.8796 | **0.0353** | n.a. | **0.0015** | **0.0066** | **0.0246** | 0.1566 |
| Aβ42 | evening | spindle (wide) | Fp2 | -0.3417 | **0.0357** | spindle (wide)_Fp2 | 38 | 4 | 33 | 5.59 | **0.0015** | 0.3315 | 2.3305 | **0.0491** | n.a. | **0.0370** | **0.0183** | **0.0163** | 0.1919 |
| Aβ42 | evening | spindle (wide) | Fz | -0.4700 | **0.0029** | spindle (wide)_Fz | 38 | 4 | 33 | 7.11 | **0.0003** | 0.3977 | 4.4085 | **0.0371** | n.a. | **0.0022** | **0.0181** | **0.0252** | 0.1661 |
| Aβ42 | evening | spindle (narrow) | F4 | -0.3478 | **0.0324** | spindle (narrow)_F4 | 38 | 4 | 33 | 6.35 | **0.0007** | 0.3666 | 5.9315 | **0.0105** | n.a. | **0.0370** | **0.0331** | **0.0037** | 0.3523 |
| Aβ42 | evening | spindle (narrow) | Fz | -0.3508 | **0.0308** | spindle (narrow)_Fz | 38 | 4 | 33 | 6.04 | **0.0009** | 0.3528 | 6.1747 | **0.0147** | n.a. | **0.0364** | **0.0372** | **0.0051** | 0.3122 |
| Aβ40 | evening | alpha | F3 | -0.3677 | **0.0231** | alpha_F3 | 38 | 4 | 33 | 3.52 | **0.0168** | 0.2144 | 5.5133 | 0.2730 | n.a. | 0.0776 | **0.0129** | 0.7721 | 0.6527 |
| Aβ40 | evening | alpha | F4 | -0.3414 | **0.0360** | alpha_F4 | 38 | 4 | 33 | 2.58 | **0.0554** | 0.1459 | 4.0402 | 0.4990 | n.a. | 0.0976 | **0.0323** | 0.9196 | 0.4905 |
| Aβ40 | evening | alpha | F7 | -0.3235 | **0.0475** | alpha_F7 | 38 | 4 | 33 | 3.89 | **0.0107** | 0.2383 | 2.4079 | 0.2660 | n.a. | 0.0720 | **0.0051** | 0.8616 | 0.8488 |
| Aβ40 | evening | alpha | Fp1 | -0.3824 | **0.0178** | alpha_Fp1 | 38 | 4 | 33 | 3.84 | **0.0114** | 0.2348 | 2.5767 | 0.3515 | n.a. | 0.0568 | **0.0091** | 0.8432 | 0.5227 |
| Aβ40 | evening | alpha | Fp2 | -0.3951 | **0.0141** | alpha_Fp2 | 38 | 4 | 33 | 4.20 | **0.0073** | 0.2573 | 2.2564 | 0.4382 | n.a. | **0.0369** | **0.0059** | 0.9883 | 0.4899 |
| Aβ40 | evening | alpha | Fz | -0.3560 | **0.0282** | alpha_Fz | 38 | 4 | 33 | 2.46 | **0.0647** | 0.1362 | 6.8579 | 0.3140 | n.a. | 0.1188 | 0.0614 | 0.6639 | 0.5006 |
| Aβ40 | evening | beta1 | C3 | -0.3254 | **0.0462** | beta1_C3 | 38 | 4 | 33 | 3.42 | **0.0191** | 0.2072 | 1.1159 | 0.6068 | n.a. | 0.0954 | **0.0069** | 0.9968 | 0.5852 |
| Aβ40 | evening | beta1 | C4 | -0.3449 | **0.0340** | beta1_C4 | 38 | 4 | 33 | 4.92 | **0.0032** | 0.2979 | 1.0393 | 0.5364 | n.a. | 0.1091 | **0.0014** | 0.6351 | 0.2818 |
| Aβ40 | evening | beta1 | Cz | -0.3574 | **0.0276** | beta1_Cz | 38 | 4 | 33 | 4.72 | **0.0040** | 0.2867 | 1.7290 | 0.4862 | n.a. | 0.1131 | **0.0026** | 0.5324 | 0.2325 |
| Aβ40 | evening | beta1 | F3 | -0.4282 | **0.0073** | beta1_F3 | 38 | 4 | 33 | 4.61 | **0.0045** | 0.2810 | 2.6150 | 0.0709 | n.a. | 0.1048 | **0.0268** | 0.1670 | 0.5690 |
| Aβ40 | evening | beta1 | F4 | -0.4354 | **0.0063** | beta1_F4 | 38 | 4 | 33 | 5.29 | **0.0021** | 0.3170 | 3.0618 | **0.0333** | n.a. | 0.1465 | **0.0310** | 0.0611 | 0.4817 |
| Aβ40 | evening | beta1 | F7 | -0.3682 | **0.0229** | beta1_F7 | 38 | 4 | 33 | 3.68 | **0.0139** | 0.2244 | 1.3199 | 0.0665 | n.a. | 0.1482 | **0.0298** | 0.3278 | 0.9219 |
| Aβ40 | evening | beta1 | Fp1 | -0.3692 | **0.0226** | beta1_Fp1 | 38 | 4 | 33 | 2.80 | **0.0415** | 0.1632 | 1.8503 | 0.0548 | n.a. | 0.2933 | 0.2314 | 0.1070 | 0.6610 |
| Aβ40 | evening | beta1 | Fp2 | -0.4335 | **0.0065** | beta1_Fp2 | 38 | 4 | 33 | 4.36 | **0.0061** | 0.2662 | 1.7888 | **0.0372** | n.a. | 0.1457 | 0.0797 | 0.0841 | 0.5398 |
| Aβ40 | evening | beta1 | Fz | -0.4606 | **0.0036** | beta1_Fz | 38 | 4 | 33 | 5.22 | **0.0023** | 0.3132 | 3.1530 | 0.0504 | n.a. | 0.0879 | **0.0358** | 0.0962 | 0.4178 |

**Table S31.** (continued)

| **AD biomarker** | **Sampling time** | **Power [Stage N3] Frequency band** | **location** | **Pearson r  [AD biomarker x power]** | **p** | **Dependent variable** | **n** | **DF model** | **DF error** | **F-value** | **model *p*** | **adjusted R-Square** | **Parameter estimate intercept** | **Intercept *p*** | **Parameter estimate EEG power parameter *p*** | **Parameter estimate AD-Biomarker (evening) *p*** | **Parameter estimate Sex *p*** | **Parameter estimate Age *p*** | **Parameter estimate BMI *p*** |
| --- | --- | --- | --- | --- | --- | --- | --- | --- | --- | --- | --- | --- | --- | --- | --- | --- | --- | --- | --- |
| Aβ40 | evening | spindle (wide) | C3 | -0.3688 | **0.0227** | spindle (wide)_C3 | 38 | 4 | 33 | 4.10 | **0.0083** | 0.2509 | 1.4478 | 0.4992 | n.a. | 0.0616 | **0.0049** | 0.8837 | 0.4859 |
| Aβ40 | evening | spindle (wide) | C4 | -0.3852 | **0.0169** | spindle (wide)_C4 | 38 | 4 | 33 | 5.54 | **0.0016** | 0.3290 | 1.1665 | 0.5122 | n.a. | 0.0567 | **0.0011** | 0.6824 | 0.2389 |
| Aβ40 | evening | spindle (wide) | Cz | -0.3854 | **0.0169** | spindle (wide)_Cz | 38 | 4 | 33 | 4.67 | **0.0042** | 0.2842 | 1.9575 | 0.4552 | n.a. | 0.0798 | **0.0041** | 0.5440 | 0.2073 |
| Aβ40 | evening | spindle (wide) | F3 | -0.4677 | **0.0031** | spindle (wide)_F3 | 38 | 4 | 33 | 4.91 | **0.0032** | 0.2969 | 2.6467 | 0.1765 | n.a. | **0.0395** | **0.0207** | 0.3214 | 0.3193 |
| Aβ40 | evening | spindle (wide) | F4 | -0.4664 | **0.0032** | spindle (wide)_F4 | 38 | 4 | 33 | 5.13 | **0.0025** | 0.3084 | 2.8041 | 0.1588 | n.a. | 0.0532 | **0.0228** | 0.2105 | 0.2245 |
| Aβ40 | evening | spindle (wide) | F7 | -0.3865 | **0.0165** | spindle (wide)_F7 | 38 | 4 | 33 | 3.53 | **0.0167** | 0.2148 | 1.4175 | 0.1931 | n.a. | 0.0888 | **0.0239** | 0.5291 | 0.7446 |
| Aβ40 | evening | spindle (wide) | F8 | -0.3346 | **0.0400** | spindle (wide)_F8 | 38 | 4 | 33 | 4.56 | **0.0049** | 0.2777 | 0.3276 | 0.7569 | n.a. | 0.2000 | **0.0046** | 0.3676 | 0.0501 |
| Aβ40 | evening | spindle (wide) | Fp1 | -0.4287 | **0.0072** | spindle (wide)_Fp1 | 38 | 4 | 33 | 3.78 | **0.0122** | 0.2312 | 1.9970 | 0.1139 | n.a. | 0.1266 | 0.0974 | 0.1503 | 0.3325 |
| Aβ40 | evening | spindle (wide) | Fp2 | -0.4720 | **0.0028** | spindle (wide)_Fp2 | 38 | 4 | 33 | 5.28 | **0.0021** | 0.3164 | 1.8292 | 0.1216 | n.a. | 0.0569 | **0.0265** | 0.1613 | 0.2350 |
| Aβ40 | evening | spindle (wide) | Fz | -0.4913 | **0.0017** | spindle (wide)_Fz | 38 | 4 | 33 | 4.96 | **0.0031** | 0.2995 | 3.1775 | 0.1551 | n.a. | **0.0350** | **0.0454** | 0.2421 | 0.2324 |
| Aβ40 | evening | spindle (narrow) | C3 | -0.3365 | **0.0389** | spindle (narrow)_C3 | 38 | 4 | 33 | 3.93 | **0.0102** | 0.2407 | 1.2532 | 0.6586 | n.a. | 0.0877 | **0.0038** | 0.9321 | 0.4433 |
| Aβ40 | evening | spindle (narrow) | C4 | -0.3540 | **0.0292** | spindle (narrow)_C4 | 38 | 4 | 33 | 5.44 | **0.0018** | 0.3244 | 1.2925 | 0.5720 | n.a. | 0.1059 | **0.0009** | 0.5496 | 0.1997 |
| Aβ40 | evening | spindle (narrow) | Cz | -0.3549 | **0.0288** | spindle (narrow)_Cz | 38 | 4 | 33 | 4.80 | **0.0037** | 0.2913 | 1.7796 | 0.6173 | n.a. | 0.1043 | **0.0020** | 0.6022 | 0.1968 |
| Aβ40 | evening | spindle (narrow) | F3 | -0.4388 | **0.0059** | spindle (narrow)_F3 | 38 | 4 | 33 | 4.78 | **0.0038** | 0.2898 | 4.2671 | 0.0589 | n.a. | 0.1180 | **0.0399** | 0.1030 | 0.4842 |
| Aβ40 | evening | spindle (narrow) | F4 | -0.4361 | **0.0062** | spindle (narrow)_F4 | 38 | 4 | 33 | 5.23 | **0.0022** | 0.3140 | 5.0926 | **0.0317** | n.a. | 0.1849 | 0.0562 | **0.0358** | 0.3996 |
| Aβ40 | evening | spindle (narrow) | F7 | -0.3902 | **0.0154** | spindle (narrow)_F7 | 38 | 4 | 33 | 3.76 | **0.0125** | 0.2299 | 2.1070 | 0.0579 | n.a. | 0.1564 | **0.0471** | 0.2040 | 0.9063 |
| Aβ40 | evening | spindle (narrow) | Fp1 | -0.3679 | **0.0230** | spindle (narrow)_Fp1 | 38 | 4 | 33 | 2.80 | **0.0415** | 0.1632 | 3.2027 | 0.0522 | n.a. | 0.3645 | 0.4063 | 0.0617 | 0.5621 |
| Aβ40 | evening | spindle (narrow) | Fp2 | -0.4326 | **0.0067** | spindle (narrow)_Fp2 | 38 | 4 | 33 | 4.43 | **0.0057** | 0.2703 | 3.0581 | **0.0325** | n.a. | 0.2051 | 0.1550 | **0.0384** | 0.4247 |
| Aβ40 | evening | spindle (narrow) | Fz | -0.4684 | **0.0030** | spindle (narrow)_Fz | 38 | 4 | 33 | 5.33 | **0.0020** | 0.3189 | 5.1774 | **0.0425** | n.a. | 0.0993 | 0.0571 | 0.0604 | 0.3655 |

**Table S32.** Significant Pearson correlations and adjusted linear regression results for sleep EEG power (Stage N3, morning AD biomarkers) and AD biomarkers. Regression models were adjusted for evening AD biomarker levels (n.a. for evening biomarker correlations), sex, age, and BMI.

| **AD biomarker** | **Sampling time** | **Power [Stage N3] Frequency band** | **loc-ation** | **Pearson r  [AD biomarker x power]** | **p** | **Dependent variable** | **n** | **DF model** | **DF error** | **F-value** | **model *p*** | **adjusted R-Square** | **Parameter estimate intercept** | **Intercept *p*** | **Parameter estimate EEG power parameter *p*** | **Parameter estimate AD-Biomarker (evening) *p*** | **Parameter estimate Sex *p*** | **Parameter estimate Age *p*** | **Parameter estimate BMI *p*** |
| --- | --- | --- | --- | --- | --- | --- | --- | --- | --- | --- | --- | --- | --- | --- | --- | --- | --- | --- | --- |
| Aβ42 | morning | alpha | Cz | -0.3458 | **0.0389** | alpha_Cz | 36 | 5 | 30 | 14.71 | **<0.0001** | 0.6620 | 0.7744 | 0.8148 | 0.4804 | **<0.0001** | 0.8612 | 0.5581 | 0.7631 |
| Aβ42 | morning | alpha | F3 | -0.4291 | **0.0090** | alpha_F3 | 36 | 5 | 30 | 14.79 | **<0.0001** | 0.6633 | 0.8138 | 0.8043 | 0.4340 | **<0.0001** | 0.7778 | 0.5334 | 0.7657 |
| Aβ42 | morning | alpha | F4 | -0.4021 | **0.0151** | alpha_F4 | 36 | 5 | 30 | 14.42 | **<0.0001** | 0.6572 | 0.2527 | 0.9379 | 0.7800 | **<0.0001** | 0.9807 | 0.4615 | 0.7345 |
| Aβ42 | morning | alpha | F7 | -0.4050 | **0.0143** | alpha_F7 | 36 | 5 | 30 | 15.96 | **<0.0001** | 0.6813 | 1.3457 | 0.6716 | 0.1353 | **<0.0001** | 0.5285 | 0.4859 | 0.6653 |
| Aβ42 | morning | alpha | F8 | -0.3626 | **0.0298** | alpha_F8 | 36 | 5 | 30 | 15.71 | **<0.0001** | 0.6775 | 0.0725 | 0.9812 | 0.1700 | **<0.0001** | 0.4715 | 0.4272 | 0.9835 |
| Aβ42 | morning | alpha | Fp1 | -0.3962 | **0.0168** | alpha_Fp1 | 36 | 5 | 30 | 14.79 | **<0.0001** | 0.6634 | 0.7072 | 0.8276 | 0.4325 | **<0.0001** | 0.7750 | 0.5416 | 0.7965 |
| Aβ42 | morning | alpha | Fp2 | -0.3722 | **0.0254** | alpha_Fp2 | 36 | 5 | 30 | 14.63 | **<0.0001** | 0.6607 | 0.5083 | 0.8751 | 0.5350 | **<0.0001** | 0.8313 | 0.5114 | 0.7820 |
| Aβ42 | morning | alpha | Fz | -0.4246 | **0.0098** | alpha_Fz | 36 | 5 | 30 | 14.55 | **<0.0001** | 0.6594 | 0.5206 | 0.8740 | 0.6043 | **<0.0001** | 0.9183 | 0.5076 | 0.7622 |
| Aβ42 | morning | beta1 | Cz | -0.3817 | **0.0216** | beta1_Cz | 36 | 5 | 30 | 15.99 | **<0.0001** | 0.6817 | 0.9211 | 0.7668 | 0.1318 | **<0.0001** | 0.4994 | 0.6689 | 0.9774 |
| Aβ42 | morning | beta1 | F3 | -0.3891 | **0.0190** | beta1_F3 | 36 | 5 | 30 | 15.21 | **<0.0001** | 0.6700 | 1.4048 | 0.6743 | 0.2732 | **<0.0001** | 0.7412 | 0.7444 | 0.8054 |
| Aβ42 | morning | beta1 | F4 | -0.4183 | **0.0111** | beta1_F4 | 36 | 5 | 30 | 15.11 | **<0.0001** | 0.6684 | 1.5734 | 0.6479 | 0.3025 | **<0.0001** | 0.7569 | 0.7992 | 0.8199 |
| Aβ42 | morning | beta1 | Fp2 | -0.3431 | **0.0405** | beta1_Fp2 | 36 | 5 | 30 | 14.85 | **<0.0001** | 0.6643 | 1.1911 | 0.7273 | 0.4028 | **<0.0001** | 0.8739 | 0.7179 | 0.7853 |
| Aβ42 | morning | beta1 | Fz | -0.4301 | **0.0088** | beta1_Fz | 36 | 5 | 30 | 15.43 | **<0.0001** | 0.6734 | 1.7156 | 0.6112 | 0.2195 | **<0.0001** | 0.7124 | 0.8483 | 0.8661 |
| Aβ42 | morning | beta1 | Pz | -0.3317 | **0.0481** | beta1_Pz | 36 | 5 | 30 | 17.17 | **<0.0001** | 0.6978 | -0.1681 | 0.9549 | 0.0511 | **<0.0001** | 0.3478 | 0.4722 | 0.8754 |
| Aβ42 | morning | spindle (wide) | C3 | -0.3458 | **0.0388** | spindle (wide)_C3 | 36 | 5 | 30 | 15.73 | **<0.0001** | 0.6778 | 0.8208 | 0.7925 | 0.1664 | **<0.0001** | 0.5842 | 0.5839 | 0.8535 |
| Aβ42 | morning | spindle (wide) | C4 | -0.3874 | **0.0196** | spindle (wide)_C4 | 36 | 5 | 30 | 15.92 | **<0.0001** | 0.6807 | 0.9180 | 0.7680 | 0.1400 | **<0.0001** | 0.4756 | 0.6585 | 0.9789 |
| Aβ42 | morning | spindle (wide) | Cz | -0.4544 | **0.0054** | spindle (wide)_Cz | 36 | 5 | 30 | 16.18 | **<0.0001** | 0.6845 | 1.1131 | 0.7205 | 0.1119 | **<0.0001** | 0.4751 | 0.6891 | 0.9778 |
| Aβ42 | morning | spindle (wide) | F3 | -0.5094 | **0.0015** | spindle (wide)_F3 | 36 | 5 | 30 | 15.74 | **<0.0001** | 0.6779 | 1.5710 | 0.6298 | 0.1654 | **<0.0001** | 0.6125 | 0.7752 | 0.9344 |
| Aβ42 | morning | spindle (wide) | F4 | -0.5276 | **0.0009** | spindle (wide)_F4 | 36 | 5 | 30 | 15.29 | **<0.0001** | 0.6712 | 1.4197 | 0.6692 | 0.2521 | **<0.0001** | 0.6780 | 0.7560 | 0.9379 |
| Aβ42 | morning | spindle (wide) | F7 | -0.3768 | **0.0235** | spindle (wide)_F7 | 36 | 5 | 30 | 15.34 | **<0.0001** | 0.6720 | 1.1344 | 0.7256 | 0.2399 | **<0.0001** | 0.7222 | 0.6156 | 0.7611 |
| Aβ42 | morning | spindle (wide) | F8 | -0.3355 | **0.0455** | spindle (wide)_F8 | 36 | 5 | 30 | 14.99 | **<0.0001** | 0.6665 | 0.3710 | 0.9060 | 0.3439 | **<0.0001** | 0.7180 | 0.6032 | 0.9796 |
| Aβ42 | morning | spindle (wide) | Fp1 | -0.4005 | **0.0155** | spindle (wide)_Fp1 | 36 | 5 | 30 | 15.22 | **<0.0001** | 0.6701 | 1.2619 | 0.7017 | 0.2701 | **<0.0001** | 0.8127 | 0.7573 | 0.8732 |
| Aβ42 | morning | spindle (wide) | Fp2 | -0.4217 | **0.0104** | spindle (wide)_Fp2 | 36 | 5 | 30 | 15.22 | **<0.0001** | 0.6701 | 1.2870 | 0.6968 | 0.2714 | **<0.0001** | 0.7328 | 0.7815 | 0.9152 |
| Aβ42 | morning | spindle (wide) | Fz | -0.5460 | **0.0006** | spindle (wide)_Fz | 36 | 5 | 30 | 15.79 | **<0.0001** | 0.6788 | 1.7212 | 0.6003 | 0.1573 | **<0.0001** | 0.6438 | 0.8359 | 0.9918 |
| Aβ42 | morning | spindle (wide) | Pz | -0.3556 | **0.0333** | spindle (wide)_Pz | 36 | 5 | 30 | 17.29 | **<0.0001** | 0.6995 | -0.0286 | 0.9923 | **0.0464** | **<0.0001** | 0.3279 | 0.4798 | 0.8743 |
| Aβ42 | morning | spindle (wide) | T3 | -0.3459 | **0.0388** | spindle (wide)_T3 | 36 | 5 | 30 | 16.33 | **<0.0001** | 0.6865 | 0.7866 | 0.7972 | 0.0991 | **<0.0001** | 0.6158 | 0.4963 | 0.8299 |
| Aβ42 | morning | spindle (wide) | T4 | -0.3348 | **0.0459** | spindle (wide)_T4 | 36 | 5 | 30 | 15.77 | **<0.0001** | 0.6785 | 0.8815 | 0.7778 | 0.1603 | **<0.0001** | 0.5925 | 0.5407 | 0.8538 |
| Aβ42 | morning | spindle (narrow) | C3 | -0.3355 | **0.0455** | spindle (narrow)_C3 | 36 | 5 | 30 | 16.15 | **<0.0001** | 0.6839 | 0.6985 | 0.8200 | 0.1156 | **<0.0001** | 0.5109 | 0.5758 | 0.8851 |
| Aβ42 | morning | spindle (narrow) | C4 | -0.3755 | **0.0240** | spindle (narrow)_C4 | 36 | 5 | 30 | 16.81 | **<0.0001** | 0.6931 | 0.9598 | 0.7520 | 0.0673 | **<0.0001** | 0.3578 | 0.7368 | 0.9350 |
| Aβ42 | morning | spindle (narrow) | Cz | -0.4277 | **0.0093** | spindle (narrow)_Cz | 36 | 5 | 30 | 16.92 | **<0.0001** | 0.6946 | 0.9639 | 0.7503 | 0.0618 | **<0.0001** | 0.3676 | 0.6962 | 0.9271 |
| Aβ42 | morning | spindle (narrow) | F3 | -0.4045 | **0.0144** | spindle (narrow)_F3 | 36 | 5 | 30 | 15.25 | **<0.0001** | 0.6706 | 1.5012 | 0.6553 | 0.2627 | **<0.0001** | 0.7515 | 0.7951 | 0.8301 |
| Aβ42 | morning | spindle (narrow) | F4 | -0.4260 | **0.0096** | spindle (narrow)_F4 | 36 | 5 | 30 | 15.15 | **<0.0001** | 0.6690 | 1.6199 | 0.6385 | 0.2916 | **<0.0001** | 0.7796 | 0.8416 | 0.8444 |
| Aβ42 | morning | spindle (narrow) | Fp2 | -0.3447 | **0.0395** | spindle (narrow)_Fp2 | 36 | 5 | 30 | 14.86 | **<0.0001** | 0.6644 | 1.2230 | 0.7211 | 0.3991 | **<0.0001** | 0.9060 | 0.7637 | 0.8094 |
| Aβ42 | morning | spindle (narrow) | Fz | -0.4466 | **0.0063** | spindle (narrow)_Fz | 36 | 5 | 30 | 15.57 | **<0.0001** | 0.6755 | 1.8844 | 0.5779 | 0.1926 | **<0.0001** | 0.7175 | 0.9164 | 0.8957 |
| Aβ42 | morning | spindle (narrow) | P4 | -0.3449 | **0.0394** | spindle (narrow)_P4 | 36 | 5 | 30 | 19.23 | **<0.0001** | 0.7225 | -0.4603 | 0.8720 | **0.0119** | **<0.0001** | 0.1715 | 0.5230 | 0.6432 |
| Aβ42 | morning | spindle (narrow) | Pz | -0.3758 | **0.0239** | spindle (narrow)_Pz | 36 | 5 | 30 | 18.77 | **<0.0001** | 0.7173 | -0.7129 | 0.8054 | **0.0162** | **<0.0001** | 0.2207 | 0.4189 | 0.6818 |
| Aβ42 | morning | spindle (narrow) | T3 | -0.3414 | **0.0416** | spindle (narrow)_T3 | 36 | 5 | 30 | 16.85 | **<0.0001** | 0.6936 | 0.9205 | 0.7614 | 0.0653 | **<0.0001** | 0.5802 | 0.5639 | 0.8718 |

**Table S32**. (continued)

| **AD biomarker** | **Sampling time** | **Power [Stage N3] Frequency band** | **loc-ation** | **Pearson r  [AD biomarker x power]** | **p** | **Dependent variable** | **n** | **DF model** | **DF error** | **F-value** | **model *p*** | **adjusted R-Square** | **Parameter estimate intercept** | **Intercept *p*** | **Parameter estimate EEG power parameter *p*** | **Parameter estimate AD-Biomarker (evening) *p*** | **Parameter estimate Sex *p*** | **Parameter estimate Age *p*** | **Parameter estimate BMI *p*** |
| --- | --- | --- | --- | --- | --- | --- | --- | --- | --- | --- | --- | --- | --- | --- | --- | --- | --- | --- | --- |
| Aβ40 | morning | alpha | Fp1 | -0.3369 | **0.0445** | alpha_Fp1 | 36 | 5 | 30 | 8.58 | **<0.0001** | 0.5198 | 141.8796 | 0.1181 | 0.8568 | **<0.0001** | 0.4258 | 0.7786 | 0.0775 |
| Aβ40 | morning | alpha | Fp2 | -0.3328 | **0.0473** | alpha_Fp2 | 36 | 5 | 30 | 8.56 | **<0.0001** | 0.5193 | 139.6521 | 0.1222 | 0.9684 | **<0.0001** | 0.3963 | 0.7699 | 0.0751 |
| Aβ40 | morning | beta1 | C4 | -0.3537 | **0.0343** | beta1_C4 | 36 | 5 | 30 | 8.69 | **<0.0001** | 0.5236 | 144.0179 | 0.1085 | 0.6049 | **<0.0001** | 0.5608 | 0.8040 | 0.0923 |
| Aβ40 | morning | beta1 | Cz | -0.3674 | **0.0275** | beta1_Cz | 36 | 5 | 30 | 8.70 | **<0.0001** | 0.5239 | 144.7228 | 0.1072 | 0.5922 | **<0.0001** | 0.5468 | 0.8133 | 0.0958 |
| Aβ40 | morning | beta1 | F3 | -0.3951 | **0.0171** | beta1_F3 | 36 | 5 | 30 | 8.69 | **<0.0001** | 0.5235 | 153.5573 | 0.1016 | 0.6088 | **<0.0001** | 0.4671 | 0.8662 | 0.0804 |
| Aβ40 | morning | beta1 | F4 | -0.4043 | **0.0145** | beta1_F4 | 36 | 5 | 30 | 8.69 | **<0.0001** | 0.5236 | 156.6426 | 0.1015 | 0.6030 | **<0.0001** | 0.4646 | 0.9065 | 0.0837 |
| Aβ40 | morning | beta1 | Fp2 | -0.3872 | **0.0196** | beta1_Fp2 | 36 | 5 | 30 | 8.64 | **<0.0001** | 0.5218 | 152.1860 | 0.1108 | 0.6915 | **<0.0001** | 0.4124 | 0.8662 | 0.0797 |
| Aβ40 | morning | beta1 | Fz | -0.4451 | **0.0065** | beta1_Fz | 36 | 5 | 30 | 8.83 | **<0.0001** | 0.5279 | 161.6752 | 0.0869 | 0.4630 | **<0.0001** | 0.5034 | 0.9389 | 0.0904 |
| Aβ40 | morning | spindle (wide) | C3 | -0.3523 | **0.0351** | spindle (wide)_C3 | 36 | 5 | 30 | 8.66 | **<0.0001** | 0.5227 | 143.7251 | 0.1097 | 0.6460 | **<0.0001** | 0.5037 | 0.7746 | 0.0813 |
| Aβ40 | morning | spindle (wide) | C4 | -0.3939 | **0.0174** | spindle (wide)_C4 | 36 | 5 | 30 | 8.75 | **<0.0001** | 0.5254 | 145.2770 | 0.1051 | 0.5370 | **0.0001** | 0.6023 | 0.8058 | 0.0988 |
| Aβ40 | morning | spindle (wide) | Cz | -0.4028 | **0.0149** | spindle (wide)_Cz | 36 | 5 | 30 | 8.79 | **<0.0001** | 0.5267 | 146.7915 | 0.1018 | 0.4954 | **<0.0001** | 0.5760 | 0.8260 | 0.1039 |
| Aβ40 | morning | spindle (wide) | F3 | -0.4666 | **0.0041** | spindle (wide)_F3 | 36 | 5 | 30 | 8.91 | **<0.0001** | 0.5306 | 156.5571 | 0.0864 | 0.3997 | **0.0001** | 0.5510 | 0.8819 | 0.0987 |
| Aβ40 | morning | spindle (wide) | F4 | -0.4503 | **0.0059** | spindle (wide)_F4 | 36 | 5 | 30 | 8.75 | **<0.0001** | 0.5253 | 152.4099 | 0.0971 | 0.5397 | **0.0001** | 0.4937 | 0.8743 | 0.0997 |
| Aβ40 | morning | spindle (wide) | F7 | -0.3557 | **0.0333** | spindle (wide)_F7 | 36 | 5 | 30 | 8.68 | **<0.0001** | 0.5232 | 148.7917 | 0.1039 | 0.6217 | **<0.0001** | 0.4625 | 0.8013 | 0.0745 |
| Aβ40 | morning | spindle (wide) | F8 | -0.3321 | **0.0479** | spindle (wide)_F8 | 36 | 5 | 30 | 8.57 | **<0.0001** | 0.5194 | 139.6262 | 0.1192 | 0.9148 | **<0.0001** | 0.4123 | 0.7838 | 0.0952 |
| Aβ40 | morning | spindle (wide) | Fp1 | -0.4158 | **0.0117** | spindle (wide)_Fp1 | 36 | 5 | 30 | 8.75 | **<0.0001** | 0.5253 | 154.3176 | 0.0959 | 0.5388 | **<0.0001** | 0.4407 | 0.8948 | 0.0928 |
| Aβ40 | morning | spindle (wide) | Fp2 | -0.4482 | **0.0061** | spindle (wide)_Fp2 | 36 | 5 | 30 | 8.71 | **<0.0001** | 0.5243 | 152.6603 | 0.0993 | 0.5766 | **<0.0001** | 0.4800 | 0.8821 | 0.0979 |
| Aβ40 | morning | spindle (wide) | Fz | -0.5008 | **0.0019** | spindle (wide)_Fz | 36 | 5 | 30 | 9.05 | **<0.0001** | 0.5348 | 160.6541 | 0.0785 | 0.3246 | **0.0002** | 0.5414 | 0.9290 | 0.1127 |
| Aβ40 | morning | spindle (narrow) | C3 | -0.3480 | **0.0375** | spindle (narrow)_C3 | 36 | 5 | 30 | 8.75 | **<0.0001** | 0.5255 | 143.1564 | 0.1088 | 0.5342 | **<0.0001** | 0.5597 | 0.7718 | 0.0851 |
| Aβ40 | morning | spindle (narrow) | C4 | -0.4023 | **0.0150** | spindle (narrow)_C4 | 36 | 5 | 30 | 8.97 | **<0.0001** | 0.5324 | 147.0178 | 0.0983 | 0.3646 | **<0.0001** | 0.7170 | 0.8473 | 0.1133 |
| Aβ40 | morning | spindle (narrow) | Cz | -0.4022 | **0.0150** | spindle (narrow)_Cz | 36 | 5 | 30 | 8.96 | **<0.0001** | 0.5320 | 145.7968 | 0.1008 | 0.3737 | **<0.0001** | 0.6734 | 0.8309 | 0.1119 |
| Aβ40 | morning | spindle (narrow) | F3 | -0.4130 | **0.0123** | spindle (narrow)_F3 | 36 | 5 | 30 | 8.73 | **<0.0001** | 0.5248 | 156.5280 | 0.0966 | 0.5573 | **<0.0001** | 0.4696 | 0.9028 | 0.0846 |
| Aβ40 | morning | spindle (narrow) | F4 | -0.4137 | **0.0121** | spindle (narrow)_F4 | 36 | 5 | 30 | 8.73 | **<0.0001** | 0.5248 | 159.0715 | 0.0968 | 0.5590 | **<0.0001** | 0.4572 | 0.9437 | 0.0886 |
| Aβ40 | morning | spindle (narrow) | F7 | -0.3389 | **0.0432** | spindle (narrow)_F7 | 36 | 5 | 30 | 8.63 | **<0.0001** | 0.5214 | 149.9047 | 0.1118 | 0.7143 | **<0.0001** | 0.4195 | 0.8283 | 0.0720 |
| Aβ40 | morning | spindle (narrow) | Fp1 | -0.3293 | **0.0499** | spindle (narrow)_Fp1 | 36 | 5 | 30 | 8.62 | **<0.0001** | 0.5212 | 149.6760 | 0.1139 | 0.7309 | **<0.0001** | 0.3595 | 0.8632 | 0.0786 |
| Aβ40 | morning | spindle (narrow) | Fp2 | -0.3951 | **0.0171** | spindle (narrow)_Fp2 | 36 | 5 | 30 | 8.66 | **<0.0001** | 0.5225 | 154.3338 | 0.1073 | 0.6552 | **<0.0001** | 0.4004 | 0.9036 | 0.0846 |
| Aβ40 | morning | spindle (narrow) | Fz | -0.4632 | **0.0044** | spindle (narrow)_Fz | 36 | 5 | 30 | 8.92 | **<0.0001** | 0.5309 | 166.4141 | 0.0789 | 0.3935 | **<0.0001** | 0.5058 | 0.9934 | 0.0966 |
| Aβ40 | morning | spindle (narrow) | P4 | -0.3547 | **0.0338** | spindle (narrow)_P4 | 36 | 5 | 30 | 9.79 | **<0.0001** | 0.5567 | 125.6791 | 0.1447 | 0.1218 | **<0.0001** | 0.9954 | 0.8133 | 0.1900 |
| Aβ40 | morning | spindle (narrow) | Pz | -0.3450 | **0.0394** | spindle (narrow)_Pz | 36 | 5 | 30 | 9.21 | **<0.0001** | 0.5397 | 123.8940 | 0.1605 | 0.2568 | **<0.0001** | 0.7866 | 0.7178 | 0.1523 |

**Table S33.** Significant Pearson correlations and adjusted linear regression results for sleep EEG power (Stage N3, overnight change of AD biomarkers) and AD biomarkers. Regression models were adjusted for evening AD biomarker levels (n.a. for evening biomarker correlations), sex, age, and BMI. Statistically significant results are shown in bold.

| **AD biomarker** | **Sampling time** | **Power [Stage N3] Frequency band** | **location** | **Pearson r  [AD biomarker x power]** | **p** | **Dependent variable** | **n** | **DF model** | **DF error** | **F-value** | **model *p*** | **adjusted R-Square** | **Parameter estimate intercept** | **Intercept *p*** | **Parameter estimate EEG power parameter *p*** | **Parameter estimate AD-Biomarker (evening) *p*** | **Parameter estimate Sex *p*** | **Parameter estimate Age *p*** | **Parameter estimate BMI *p*** |
| --- | --- | --- | --- | --- | --- | --- | --- | --- | --- | --- | --- | --- | --- | --- | --- | --- | --- | --- | --- |
| t-tau | overnight change | beta2 | Fp2 | -0.3461 | **0.0387** | beta2_Fp2 | 36 | 5 | 30 | 6.24 | **0.0004** | 0.4280 | -2.4691 | 0.2888 | 0.3784 | **<0.0001** | 0.3390 | 0.4892 | 0.6762 |
| t-tau | overnight change | spindle (wide) | F3 | -0.3401 | **0.0424** | spindle (wide)_F3 | 36 | 5 | 30 | 7.31 | **0.0001** | 0.4741 | -1.8477 | 0.4104 | 0.0712 | **<0.0001** | 0.4110 | 0.8969 | 0.3427 |
| Aβ42 | overnight change | alpha | O1 | 0.3317 | **0.0481** | alpha_O1 | 36 | 5 | 30 | 2.41 | 0.0595 | 0.1678 | -1.9563 | 0.5104 | **0.0166** | **0.0212** | 0.2863 | 0.4773 | 0.3979 |
| Aβ42 | overnight change | beta1 | O1 | 0.3404 | **0.0422** | beta1_O1 | 36 | 5 | 30 | 2.39 | 0.0613 | 0.1658 | -0.9777 | 0.7365 | **0.0173** | **0.0173** | 0.3076 | 0.5304 | 0.7486 |
| Aβ42 | overnight change | delta | O2 | 0.3347 | **0.0460** | delta_O2 | 36 | 5 | 30 | 1.83 | 0.1365 | 0.1063 | -2.1908 | 0.4877 | 0.0566 | 0.1095 | 0.3887 | 0.6239 | 0.3746 |
| Aβ42 | overnight change | theta | O1 | 0.3338 | **0.0466** | theta_O1 | 36 | 5 | 30 | 2.26 | 0.0739 | 0.1525 | -0.3455 | 0.9056 | **0.0226** | **0.0285** | 0.2942 | 0.2765 | 0.5761 |
| Aβ42 | overnight change | theta | O2 | 0.3379 | **0.0438** | theta_O2 | 36 | 5 | 30 | 2.22 | 0.0781 | 0.1486 | -0.7052 | 0.8095 | **0.0244** | **0.0375** | 0.2830 | 0.3144 | 0.5167 |
| Aβ42 | overnight change | spindle (wide) | O1 | 0.3471 | **0.0381** | spindle (wide)_O1 | 36 | 5 | 30 | 2.52 | 0.0512 | 0.1781 | -1.1559 | 0.6893 | **0.0135** | **0.0160** | 0.2730 | 0.5189 | 0.6922 |
| Aβ42 | overnight change | spindle (wide) | O2 | 0.3311 | **0.0486** | spindle (wide)_O2 | 36 | 5 | 30 | 2.14 | 0.0875 | 0.1403 | -0.6283 | 0.8307 | **0.0288** | **0.0257** | 0.3678 | 0.4357 | 0.7071 |
| Aβ42 | overnight change | spindle (narrow) | O1 | 0.3803 | **0.0221** | spindle (narrow)_O1 | 36 | 5 | 30 | 2.97 | **0.0270** | 0.2196 | -0.7597 | 0.7862 | **0.0057** | **0.0109** | 0.2082 | 0.5141 | 0.8685 |
| Aβ42 | overnight change | spindle (narrow) | O2 | 0.3535 | **0.0344** | spindle (narrow)_O2 | 36 | 5 | 30 | 2.37 | 0.0630 | 0.1638 | -0.1487 | 0.9590 | **0.0180** | **0.0211** | 0.3220 | 0.4159 | 0.8720 |
| Aβ40 | overnight change | theta | O1 | 0.3446 | **0.0396** | theta_O1 | 36 | 5 | 30 | 2.55 | **0.0490** | 0.1811 | -143.6550 | 0.0899 | 0.0614 | 0.1069 | 0.9177 | 0.7834 | 0.0573 |
| Aβ40 | overnight change | theta | O2 | 0.3645 | **0.0288** | theta_O2 | 36 | 5 | 30 | 2.83 | **0.0330** | 0.2070 | -155.8041 | 0.0639 | **0.0349** | 0.1412 | 0.7708 | 0.8696 | **0.0454** |

**Table S34.** Significant Pearson correlations and adjusted linear regression results for sleep EEG power (NREM, evening AD biomarkers) and AD biomarkers. Regression models were adjusted for evening AD biomarker levels (n.a. for evening biomarker correlations), sex, age, and BMI. Statistically significant results are shown in bold.

| **AD biomarker** | **Sampling time** | **Power [NREM] Frequency band** | **loc-ation** | **Pearson r  [AD biomarker x power]** | **p** | **Dependent variable** | **n** | **DF model** | **DF error** | **F-value** | **model *p*** | **adjusted R-Square** | **Parameter estimate intercept** | **Intercept *p*** | **Parameter estimate EEG power parameter *p*** | **Parameter estimate AD-Biomarker (evening) *p*** | **Parameter estimate Sex *p*** | **Parameter estimate Age *p*** | **Parameter estimate BMI *p*** |
| --- | --- | --- | --- | --- | --- | --- | --- | --- | --- | --- | --- | --- | --- | --- | --- | --- | --- | --- | --- |
| p-tau181 | evening | delta | Pz | -0.3378 | **0.0354** | delta_Pz | 39 | 4 | 34 | 6.95 | **0.0003** | 0.3853 | 109.4698 | **0.0499** | n.a. | 0.3049 | **0.0006** | 0.6265 | 0.2912 |
| Aβ42 | evening | alpha | F3 | -0.3424 | **0.0329** | alpha_F3 | 39 | 4 | 34 | 5.66 | **0.0013** | 0.3290 | 5.8664 | 0.1185 | n.a. | **0.0084** | **0.0010** | 0.7369 | 0.9790 |
| Aβ42 | evening | alpha | F4 | -0.3763 | **0.0182** | alpha_F4 | 39 | 4 | 34 | 5.42 | **0.0017** | 0.3176 | 6.1856 | 0.1302 | n.a. | **0.0051** | **0.0018** | 0.7340 | 0.8832 |
| Aβ42 | evening | alpha | Fz | -0.3781 | **0.0176** | alpha_Fz | 39 | 4 | 34 | 5.39 | **0.0018** | 0.3162 | 7.5351 | 0.0936 | n.a. | **0.0055** | **0.0022** | 0.6098 | 0.8799 |
| Aβ42 | evening | beta1 | F4 | -0.3200 | **0.0470** | beta1_F4 | 39 | 4 | 34 | 7.19 | **0.0003** | 0.3944 | 3.9271 | **0.0175** | n.a. | **0.0317** | **0.0027** | **0.0115** | 0.3304 |
| Aβ42 | evening | spindle (wide) | F3 | -0.3478 | **0.0300** | spindle (wide)_F3 | 39 | 4 | 34 | 6.20 | **0.0007** | 0.3538 | 3.1501 | 0.1050 | n.a. | **0.0147** | **0.0015** | 0.1116 | 0.2588 |
| Aβ42 | evening | spindle (wide) | F4 | -0.3894 | **0.0143** | spindle (wide)_F4 | 39 | 4 | 34 | 8.13 | **0.0001** | 0.4288 | 3.9318 | **0.0345** | n.a. | **0.0054** | **0.0009** | **0.0332** | 0.2185 |
| Aβ42 | evening | spindle (wide) | Fp2 | -0.3170 | **0.0493** | spindle (wide)_Fp2 | 39 | 4 | 34 | 5.56 | **0.0015** | 0.3242 | 2.6254 | **0.0347** | n.a. | **0.0338** | **0.0048** | 0.0560 | 0.5182 |
| Aβ42 | evening | spindle (wide) | Fz | -0.3771 | **0.0179** | spindle (wide)_Fz | 39 | 4 | 34 | 6.65 | **0.0005** | 0.3729 | 4.0485 | 0.0573 | n.a. | **0.0098** | **0.0027** | 0.0527 | 0.2187 |
| Aβ42 | evening | spindle (narrow) | F4 | -0.3369 | **0.0360** | spindle (narrow)_F4 | 39 | 4 | 34 | 7.87 | **0.0001** | 0.4197 | 5.6647 | **0.0150** | n.a. | **0.0239** | **0.0028** | **0.0058** | 0.2380 |
| Aβ42 | evening | spindle (narrow) | Fz | -0.3326 | **0.0385** | spindle (narrow)_Fz | 39 | 4 | 34 | 6.64 | **0.0005** | 0.3724 | 5.4371 | **0.0330** | n.a. | **0.0290** | **0.0045** | 0.0151 | 0.2232 |
| Aβ40 | evening | alpha | Fp2 | -0.3327 | **0.0385** | alpha_Fp2 | 39 | 4 | 34 | 4.66 | **0.0042** | 0.2780 | 2.8071 | 0.2980 | n.a. | **0.0296** | **0.0023** | 0.6164 | 0.7935 |
| Aβ40 | evening | alpha | Fz | -0.3215 | **0.0460** | alpha_Fz | 39 | 4 | 34 | 3.73 | **0.0127** | 0.2235 | 5.3887 | 0.2530 | n.a. | 0.0632 | **0.0060** | 0.8929 | 0.9933 |
| Aβ40 | evening | beta1 | C4 | -0.3301 | **0.0401** | beta1_C4 | 39 | 4 | 34 | 3.96 | **0.0096** | 0.2376 | 2.5496 | 0.3489 | n.a. | 0.9289 | **0.0042** | 0.1023 | 0.3325 |
| Aβ40 | evening | beta1 | Cz | -0.3226 | **0.0452** | beta1_Cz | 39 | 4 | 34 | 3.78 | **0.0120** | 0.2265 | 3.5366 | 0.3256 | n.a. | 0.6383 | **0.0045** | 0.0728 | 0.1631 |
| Aβ40 | evening | beta1 | F3 | -0.4116 | **0.0092** | beta1_F3 | 39 | 4 | 34 | 3.97 | **0.0095** | 0.2383 | 3.1261 | 0.1299 | n.a. | 0.6751 | **0.0087** | **0.0304** | 0.3207 |
| Aβ40 | evening | beta1 | F4 | -0.4225 | **0.0074** | beta1_F4 | 39 | 4 | 34 | 5.43 | **0.0017** | 0.3180 | 4.2899 | **0.0318** | n.a. | 0.4246 | **0.0066** | **0.0040** | 0.2792 |
| Aβ40 | evening | beta1 | F7 | -0.3512 | **0.0283** | beta1_F7 | 39 | 4 | 34 | 3.24 | **0.0236** | 0.1906 | 1.5477 | 0.1615 | n.a. | 0.8571 | **0.0155** | 0.1362 | 0.9816 |
| Aβ40 | evening | beta1 | Fp1 | -0.3990 | **0.0119** | beta1_Fp1 | 39 | 4 | 34 | 3.37 | **0.0200** | 0.1995 | 2.2306 | 0.0592 | n.a. | 0.8858 | **0.0406** | **0.0258** | 0.6900 |
| Aβ40 | evening | beta1 | Fp2 | -0.4376 | **0.0053** | beta1_Fp2 | 39 | 4 | 34 | 3.88 | **0.0106** | 0.2327 | 2.3426 | 0.0523 | n.a. | 0.8827 | **0.0280** | **0.0172** | 0.6172 |
| Aβ40 | evening | beta1 | Fz | -0.4360 | **0.0055** | beta1_Fz | 39 | 4 | 34 | 4.29 | **0.0064** | 0.2573 | 3.9163 | 0.0886 | n.a. | 0.7014 | **0.0129** | **0.0137** | 0.2813 |
| Aβ40 | evening | beta2 | Fp2 | -0.3471 | **0.0304** | beta2_Fp2 | 39 | 4 | 34 | 4.64 | **0.0043** | 0.2769 | 0.3171 | 0.2845 | n.a. | **0.0146** | **0.0028** | 0.3733 | 0.7570 |
| Aβ40 | evening | beta2 | Fz | -0.3214 | **0.0460** | beta2_Fz | 39 | 4 | 34 | 4.92 | **0.0031** | 0.2921 | 0.3170 | 0.5372 | n.a. | **0.0382** | **0.0008** | 0.6285 | 0.6553 |

**Table S34.** (continued)

| **AD biomarker** | **Sampling time** | **Power [NREM] Frequency band** | **loc-ation** | **Pearson r  [AD biomarker x power]** | **p** | **Dependent variable** | **n** | **DF model** | **DF error** | **F-value** | **model *p*** | **adjusted R-Square** | **Parameter estimate intercept** | **Intercept *p*** | **Parameter estimate EEG power parameter *p*** | **Parameter estimate AD-Biomarker (evening) *p*** | **Parameter estimate Sex *p*** | **Parameter estimate Age *p*** | **Parameter estimate BMI *p*** |
| --- | --- | --- | --- | --- | --- | --- | --- | --- | --- | --- | --- | --- | --- | --- | --- | --- | --- | --- | --- |
| Aβ40 | evening | spindle (wide) | C4 | -0.3350 | **0.0371** | spindle (wide)_C4 | 39 | 4 | 34 | 4.87 | **0.0032** | 0.2896 | 2.4317 | 0.3060 | n.a. | 0.1183 | **0.0018** | 0.5172 | 0.4298 |
| Aβ40 | evening | spindle (wide) | Cz | -0.3239 | **0.0443** | spindle (wide)_Cz | 39 | 4 | 34 | 4.28 | **0.0065** | 0.2566 | 2.4721 | 0.4197 | n.a. | 0.1594 | **0.0034** | 0.4834 | 0.2553 |
| Aβ40 | evening | spindle (wide) | F3 | -0.4180 | **0.0081** | spindle (wide)_F3 | 39 | 4 | 34 | 5.53 | **0.0015** | 0.3227 | 2.2847 | 0.2441 | n.a. | **0.0364** | **0.0026** | 0.5320 | 0.3651 |
| Aβ40 | evening | spindle (wide) | F4 | -0.4327 | **0.0059** | spindle (wide)_F4 | 39 | 4 | 34 | 6.32 | **0.0006** | 0.3591 | 3.0394 | 0.1156 | n.a. | **0.0490** | **0.0025** | 0.2437 | 0.3203 |
| Aβ40 | evening | spindle (wide) | F7 | -0.3471 | **0.0304** | spindle (wide)_F7 | 39 | 4 | 34 | 4.22 | **0.0070** | 0.2534 | 1.5296 | 0.1926 | n.a. | 0.0665 | **0.0056** | 0.7932 | 0.9970 |
| Aβ40 | evening | spindle (wide) | Fp1 | -0.4097 | **0.0096** | spindle (wide)_Fp1 | 39 | 4 | 34 | 4.76 | **0.0037** | 0.2837 | 2.0280 | 0.0854 | n.a. | 0.0619 | **0.0102** | 0.3534 | 0.6721 |
| Aβ40 | evening | spindle (wide) | Fp2 | -0.4407 | **0.0050** | spindle (wide)_Fp2 | 39 | 4 | 34 | 5.58 | **0.0014** | 0.3255 | 2.1299 | 0.0816 | n.a. | **0.0325** | **0.0059** | 0.3838 | 0.6658 |
| Aβ40 | evening | spindle (wide) | Fz | -0.4426 | **0.0048** | spindle (wide)_Fz | 39 | 4 | 34 | 5.62 | **0.0014** | 0.3273 | 3.0652 | 0.1577 | n.a. | **0.0377** | **0.0052** | 0.3452 | 0.3184 |
| Aβ40 | evening | spindle (narrow) | C4 | -0.3268 | **0.0423** | spindle (narrow)_C4 | 39 | 4 | 34 | 5.23 | **0.0022** | 0.3079 | 2.7966 | 0.3178 | n.a. | 0.1584 | **0.0013** | 0.3846 | 0.3308 |
| Aβ40 | evening | spindle (narrow) | F3 | -0.4184 | **0.0080** | spindle (narrow)_F3 | 39 | 4 | 34 | 5.74 | **0.0012** | 0.3331 | 3.4441 | 0.1346 | n.a. | 0.0670 | **0.0040** | 0.2367 | 0.3434 |
| Aβ40 | evening | spindle (narrow) | F4 | -0.4256 | **0.0069** | spindle (narrow)_F4 | 39 | 4 | 34 | 6.64 | **0.0005** | 0.3724 | 4.7919 | **0.0438** | n.a. | 0.1157 | **0.0055** | 0.0586 | 0.3163 |
| Aβ40 | evening | spindle (narrow) | F7 | -0.3701 | **0.0204** | spindle (narrow)_F7 | 39 | 4 | 34 | 4.63 | **0.0043** | 0.2767 | 2.1721 | 0.0851 | n.a. | 0.0895 | **0.0078** | 0.3974 | 0.9591 |
| Aβ40 | evening | spindle (narrow) | Fp1 | -0.4102 | **0.0095** | spindle (narrow)_Fp1 | 39 | 4 | 34 | 4.46 | **0.0053** | 0.2668 | 3.0069 | **0.0349** | n.a. | 0.1301 | **0.0421** | 0.1158 | 0.6768 |
| Aβ40 | evening | spindle (narrow) | Fp2 | -0.4437 | **0.0047** | spindle (narrow)_Fp2 | 39 | 4 | 34 | 5.62 | **0.0014** | 0.3272 | 3.2507 | **0.0236** | n.a. | 0.0825 | **0.0220** | 0.0887 | 0.6440 |
| Aβ40 | evening | spindle (narrow) | Fz | -0.4396 | **0.0051** | spindle (narrow)_fz | 39 | 4 | 34 | 5.99 | **0.0009** | 0.3442 | 4.4554 | 0.0823 | n.a. | 0.0696 | **0.0072** | 0.1358 | 0.3061 |

**Table S35.** Significant Pearson correlations and adjusted linear regression results for sleep EEG power (NREM, morning AD biomarkers) and AD biomarkers. Regression models were adjusted for evening AD biomarker levels (n.a. for evening biomarker correlations), sex, age, and BMI. Statistically significant results are shown in bold.

| **AD biomarker** | **Sampling time** | **Power [NREM] Frequency band** | **Loc-ation** | **Pearson r  [AD biomarker x power]** | **p** | **Dependent variable** | **n** | **DF model** | **DF error** | **F-value** | **model *p*** | **adjusted R-Square** | **Parameter estimate intercept** | **Intercept *p*** | **Parameter estimate EEG power parameter *p*** | **Parameter estimate AD-Biomarker (evening) *p*** | **Parameter estimate Sex *p*** | **Parameter estimate Age *p*** | **Parameter estimate BMI *p*** |
| --- | --- | --- | --- | --- | --- | --- | --- | --- | --- | --- | --- | --- | --- | --- | --- | --- | --- | --- | --- |
| Aβ42 | morning | alpha | Fz | -0.3305 | **0.0457** | alpha_Fz | 37 | 5 | 31 | 12.87 | **<0.0001** | 0.6225 | 1.2171 | 0.7313 | 0.7951 | **<0.0001** | 0.8067 | 0.4919 | 0.4179 |
| Aβ42 | morning | spindle (wide) | F3 | -0.3628 | **0.0273** | spindle (wide)_F3 | 37 | 5 | 31 | 13.14 | **<0.0001** | 0.6278 | 1.6368 | 0.6408 | 0.4799 | **<0.0001** | 0.6350 | 0.6145 | 0.5061 |
| Aβ42 | morning | spindle (wide) | F4 | -0.3809 | **0.0200** | spindle (wide)_F4 | 37 | 5 | 31 | 12.96 | **<0.0001** | 0.6242 | 1.5432 | 0.6701 | 0.6454 | **<0.0001** | 0.7221 | 0.6090 | 0.4824 |
| Aβ42 | morning | spindle (wide) | Fp1 | -0.3256 | **0.0492** | spindle (wide)_Fp1 | 37 | 5 | 31 | 13.13 | **<0.0001** | 0.6276 | 1.8252 | 0.6122 | 0.4875 | **<0.0001** | 0.6710 | 0.6382 | 0.4586 |
| Aβ42 | morning | spindle (wide) | Fp2 | -0.3301 | **0.0460** | spindle (wide)_Fp2 | 37 | 5 | 31 | 13.08 | **<0.0001** | 0.6265 | 1.7558 | 0.6270 | 0.5309 | **<0.0001** | 0.6841 | 0.6288 | 0.4568 |
| Aβ42 | morning | spindle (wide) | Fz | -0.3818 | **0.0197** | spindle (wide)_Fz | 37 | 5 | 31 | 13.04 | **<0.0001** | 0.6258 | 1.6186 | 0.6504 | 0.5627 | **<0.0001** | 0.6901 | 0.6239 | 0.5005 |
| Aβ42 | morning | spindle (narrow) | Cz | -0.3337 | **0.0436** | spindle (narrow)_Cz | 37 | 5 | 31 | 13.95 | **<0.0001** | 0.6427 | 1.6613 | 0.6195 | 0.1861 | **<0.0001** | 0.4243 | 0.6807 | 0.6321 |
| Aβ42 | morning | spindle (narrow) | F3 | -0.3291 | **0.0467** | spindle (narrow)_F3 | 37 | 5 | 31 | 13.04 | **<0.0001** | 0.6257 | 1.6026 | 0.6530 | 0.5645 | **<0.0001** | 0.6923 | 0.6349 | 0.4902 |
| Aβ42 | morning | spindle (narrow) | F4 | -0.3428 | **0.0378** | spindle (narrow)_F4 | 37 | 5 | 31 | 12.94 | **<0.0001** | 0.6239 | 1.5929 | 0.6673 | 0.6690 | **<0.0001** | 0.7463 | 0.6504 | 0.4751 |
| Aβ42 | morning | spindle (narrow) | Fz | -0.3536 | **0.0318** | spindle (narrow)_Fz | 37 | 5 | 31 | 13.06 | **<0.0001** | 0.6262 | 1.7474 | 0.6293 | 0.5407 | **<0.0001** | 0.6874 | 0.6784 | 0.5033 |
| Aβ42 | morning | spindle (narrow) | Pz | -0.3323 | **0.0445** | spindle (narrow)_Pz | 37 | 5 | 31 | 15.46 | **<0.0001** | 0.6676 | 0.9314 | 0.7698 | 0.0468 | **<0.0001** | 0.2446 | 0.6041 | 0.8021 |
| Aβ40 | morning | spindle (wide) | Fz | -0.3394 | **0.0399** | spindle (wide)_Fz | 37 | 5 | 31 | 9.28 | **<0.0001** | 0.5349 | 164.2259 | 0.0927 | 0.9198 | **<0.0001** | 0.4939 | 0.9626 | **0.0414** |
| Aβ40 | morning | spindle (narrow) | Fz | -0.3393 | **0.0399** | spindle (narrow)_Fz | 37 | 5 | 31 | 9.28 | **<0.0001** | 0.5348 | 164.9007 | 0.0964 | 0.9537 | **<0.0001** | 0.5034 | 0.9653 | **0.0423** |
| Aβ42/40 | morning | theta | Cz | -0.3269 | **0.0483** | theta_Cz | 37 | 5 | 31 | 11.34 | **<0.0001** | 0.5895 | -0.0136 | 0.4769 | 0.0581 | **<0.0001** | 0.1558 | 0.9403 | **0.0137** |
| Aβ42/40 | morning | theta | F3 | -0.3270 | **0.0482** | theta_F3 | 37 | 5 | 31 | 11.10 | **<0.0001** | 0.5838 | -0.0148 | 0.4404 | 0.0752 | **<0.0001** | 0.1769 | 0.8146 | **0.0212** |
| Aβ42/40 | morning | theta | F4 | -0.3257 | **0.0492** | theta_F4 | 37 | 5 | 31 | 11.03 | **<0.0001** | 0.5822 | -0.0164 | 0.3874 | 0.0807 | **<0.0001** | 0.2095 | 0.8203 | **0.0169** |
| Aβ42/40 | morning | theta | Fz | -0.3286 | **0.0471** | theta_Fz | 37 | 5 | 31 | 11.10 | **<0.0001** | 0.5839 | -0.0152 | 0.4278 | 0.0748 | **<0.0001** | 0.1767 | 0.8436 | **0.0161** |
| Aβ42/40 | morning | theta | Pz | -0.3342 | **0.0432** | theta_Pz | 37 | 5 | 31 | 11.10 | **<0.0001** | 0.5839 | -0.0154 | 0.4217 | 0.0748 | **<0.0001** | 0.1921 | 0.8627 | **0.0176** |

**Table S36.** Significant Pearson correlations and adjusted linear regression results for sleep EEG power (Stage R, evening AD biomarkers) and AD biomarkers. Regression models were adjusted for evening AD biomarker levels (n.a. for evening biomarker correlations), sex, age, and BMI. Statistically significant results are shown in bold.

| **AD biomarker** | **Sampling time** | **Power [Stage R] Frequency band** | **loc-ation** | **Pearson r  [AD biomarker x power]** | **p** | **Dependent variable** | **n** | **DF model** | **DF error** | **F-value** | **model *p*** | **adjusted R-Square** | **Parameter estimate intercept** | **Intercept *p*** | **Parameter estimate EEG power parameter *p*** | **Parameter estimate AD-Biomarker (evening) *p*** | **Parameter estimate Sex *p*** | **Parameter estimate Age *p*** | **Parameter estimate BMI *p*** |
| --- | --- | --- | --- | --- | --- | --- | --- | --- | --- | --- | --- | --- | --- | --- | --- | --- | --- | --- | --- |
| p-tau217 | evening | theta | F4 | -0.3540 | **0.0271** | theta_F4 | 39 | 4 | 34 | 4.11 | **0.0080** | 0.2466 | 1.2688 | 0.8080 | n.a. | 0.0926 | **0.0035** | 0.9564 | 0.2939 |
| p-tau217 | evening | theta | Fz | -0.3496 | **0.0291** | theta_Fz | 39 | 4 | 34 | 4.56 | **0.0047** | 0.2725 | 3.8825 | 0.5368 | n.a. | 0.1422 | **0.0022** | 0.6961 | 0.3675 |
| p-tau181 | evening | theta | F3 | -0.3739 | **0.0190** | theta_F3 | 39 | 4 | 34 | 5.04 | **0.0027** | 0.2983 | 3.8901 | 0.3805 | n.a. | 0.1554 | **0.0017** | 0.6097 | 0.5428 |
| p-tau181 | evening | theta | F4 | -0.3750 | **0.0187** | theta_F4 | 39 | 4 | 34 | 3.93 | **0.0100** | 0.2358 | 2.7176 | 0.5898 | n.a. | 0.1252 | **0.0074** | 0.6384 | 0.3071 |
| p-tau181 | evening | theta | F7 | -0.3492 | **0.0293** | theta_F7 | 39 | 4 | 34 | 4.27 | **0.0066** | 0.2562 | 2.2165 | 0.3488 | n.a. | 0.1581 | **0.0041** | 0.9721 | 0.8882 |
| p-tau181 | evening | theta | Fp1 | -0.3445 | **0.0317** | theta_Fp1 | 39 | 4 | 34 | 3.98 | **0.0094** | 0.2387 | 2.4698 | 0.3907 | n.a. | 0.2151 | **0.0047** | 0.5738 | 0.4500 |
| p-tau181 | evening | theta | Fp2 | -0.3547 | **0.0267** | theta_Fp2 | 39 | 4 | 34 | 4.16 | **0.0075** | 0.2497 | 2.2663 | 0.4263 | n.a. | 0.1909 | **0.0043** | 0.5295 | 0.3387 |
| p-tau181 | evening | theta | Fz | -0.3780 | **0.0177** | theta_Fz | 39 | 4 | 34 | 4.49 | **0.0051** | 0.2688 | 5.2939 | 0.3813 | n.a. | 0.1586 | **0.0043** | 0.4499 | 0.3759 |
| Aβ40 | evening | beta2 | Fz | -0.3296 | **0.0405** | beta2_Fz | 39 | 4 | 34 | 4.03 | **0.0088** | 0.2421 | 0.6526 | 0.4902 | n.a. | 0.0620 | **0.0033** | 0.9288 | 0.6383 |
| Aβ42/40 | evening | theta | O2 | -0.3527 | **0.0256** | theta_02 | 40 | 4 | 35 | 7.54 | **0.0002** | 0.4014 | 7.1641 | 0.1170 | n.a. | **0.0031** | **0.0001** | 0.5415 | 0.8639 |

**Table S37.** Significant Pearson correlations and adjusted linear regression results for sleep EEG power (Stage R, morning AD biomarkers) and AD biomarkers. Regression models were adjusted for evening AD biomarker levels (n.a. for evening biomarker correlations), sex, age, and BMI. Statistically significant results are shown in bold.

| **AD biomarker** | **Sampling time** | **Power [Stage R] Frequency band** | **location** | **Pearson r  [AD biomarker x power]** | **p** | **Dependent variable** | **n** | **DF model** | **DF error** | **F-value** | **model *p*** | **adjusted R-Square** | **Parameter estimate intercept** | **Intercept *p*** | **Parameter estimate EEG power parameter *p*** | **Parameter estimate AD-Biomarker (evening) *p*** | **Parameter estimate Sex *p*** | **Parameter estimate Age *p*** | **Parameter estimate BMI *p*** |
| --- | --- | --- | --- | --- | --- | --- | --- | --- | --- | --- | --- | --- | --- | --- | --- | --- | --- | --- | --- |
| Aβ42/40 | morning | theta | Pz | -0.3612 | **0.0281** | Theta_Pz | 37 | 5 | 31 | 10.6 | **<0.0001** | 0.5704 | -0.0128 | 0.5304 | 0.1382 | **<0.0001** | 0.2536 | 0.9281 | **0.0257** |

**Table S38.** Significant Pearson correlations and adjusted linear regression results for sleep EEG power (Stage R, overnight change of AD biomarkers) and AD biomarkers. Regression models were adjusted for evening AD biomarker levels (n.a. for evening biomarker correlations), sex, age, and BMI. Statistically significant results are shown in bold.

| **AD biomarker** | **Sampling time** | **Power [Stage R] Frequency band** | **loc-ation** | **Pearson r  [AD biomarker x power]** | **p** | **Dependent variable** | **n** | **DF model** | **DF error** | **F-value** | **model *p*** | **adjusted R-Square** | **Parameter estimate intercept** | **Intercept *p*** | **Parameter estimate EEG power parameter *p*** | **Parameter estimate AD-Biomarker (evening) *p*** | **Parameter estimate Sex *p*** | **Parameter estimate Age *p*** | **Parameter estimate BMI *p*** |
| --- | --- | --- | --- | --- | --- | --- | --- | --- | --- | --- | --- | --- | --- | --- | --- | --- | --- | --- | --- |
| p-tau217 | overnight change | theta | O2 | -0.3435 | **0.0323** | p-tau217 | 39 | 5 | 33 | 2.18 | 0.0798 | 0.1347 | 0.2615 | 0.4678 | **0.0346** | **0.0267** | 0.5914 | 0.1988 | 0.8255 |
| Aβ42/40 | overnight change | spindle (narrow) | T6 | -0.3449 | **0.0366** | spindle (narrow)_T6 | 37 | 5 | 31 | 3.00 | **0.0254** | 0.2173 | -0.0325 | 0.5983 | **0.0104** | 0.2551 | 0.0655 | 0.1857 | 0.2219 |
